# Glycosylation of 3-Hydroxyflavone, 3-Methoxyflavone, Quercetin and Baicalein in Fungal Cultures of the Genus *Isaria*

**DOI:** 10.3390/molecules23102477

**Published:** 2018-09-27

**Authors:** Monika Dymarska, Tomasz Janeczko, Edyta Kostrzewa-Susłow

**Affiliations:** Department of Chemistry, Faculty of Biotechnology and Food Science, Wrocław University of Environmental and Life Sciences, 50-375 Wrocław, Poland; janeczko13@interia.pl (T.J.); ekostrzew@gmail.com (E.K.-S.)

**Keywords:** biotransformations, flavonoids, 3-hydroxyflavone, 3-methoxyflavone, quercetin, isoquercetin, baicalein, glycosylation, *O*-methylglucosides, *Isaria*

## Abstract

Flavonoids are plant secondary metabolites with a broad spectrum of biological activities. In nature, they occur mainly in the form of glycosides, but their extraction is often difficult and expensive, as is chemical synthesis. We have shown that biotransformations are an excellent method for obtaining flavonoid glycosides. We are the first team to describe the use of *Isaria* microorganisms in biotransformations of flavonoid compounds. In the present study as biocatalysts, we used one strain of *Isaria fumosorosea* KCH J2 isolated from a spider carcass in green areas of Wroclaw and two strains of *I. farinosa* (J1.4 and J1.6) isolated from insects found in already unused mines in Lower Silesia. The substrates were 3-hydroxyflavone, 3-methoxyflavone, quercetin (3,3′,4′,5,7-pentahydroxyflavone), and baicalein (5,6,7-trihydroxyflavone). For all the substrates that were used in this study, we obtained 4-*O*-methylglucopyranosides. In the case of substrates with a hydroxyl group in the third position, *O*-*β*-d-glucopyranosides were also formed. Isoquercetin that was obtained by biotransformation was used as a substrate to check the kinetics of the formation of flavonoid 4-*O*-methylglucopyranosides in *I. fumosorosea* KCH J2 culture. We did not observe the attachment of the methyl group to glucose unit in isoquercetin. Our finding suggest that the attachment of 4-*O*-methylglucopyranose occurs in one step.

## 1. Introduction

Flavonoids are a large group of plant secondary metabolites with diverse biological activities. Consumed with foods of plant origin, these compounds are an important factor in the prevention of many civilizational diseases (diabetes, heart diseases, and even depression) [1,2,3,4,5,6,7].

Quercetin (**3**) can be found in almost all vegetables and fruits [8] and it is one of the most widely described flavonoid compounds. Its consumption is high and it ranges from several to a dozen or so mg per day. Good sources of quercetin are onion, cranberry, apples, or blueberries [8,9]. This flavonol shows high and diverse biological activities: anticancer, antibacterial, antiviral, anti-obesity, antidiabetic, anti-inflammatory as well as cytoprotective, neuroprotective and hepatoprotective and it plays an important role in the prevention of coronary artery, asthma, and Alzheimer diseases. Quercetin is also considered to be an immune booster and a remedy for mood disorders [10,11,12,13,14,15,16,17,18]. 

Baicalein (**4**), which is another widely described flavonoid of natural origin, belongs to flavones and it has three hydroxyl groups in the A ring. The presence of these substituents results in strong antioxidant potential. The natural source of baicalein is the *Scutellaria baicalensis* root, which contains 5.41% of this flavonoid. A huge number of studies carried out both in vitro and in vivo (including humans) show biological activity of formulations containing *S. baicalensis* extracts, as well as flavonoids that were isolated mainly from its root [19,20,21].

A large part of the biological activity results from the antioxidant capacity of flavonoid compounds. Studies on the relationship between the structure of the flavonoid compound and its antioxidant potential show that the presence of a C2-C3 double bond, as well as a hydroxyl group at C-3, and a catechol group in the B ring are essential [22,23,24,25]. The high biological activity that is shown by the flavonoid compounds is also strongly associated with their ability to bind to biological membranes [26]. 3-Hydroxyflavone (**1**) has the potential to bind with biomacromolecules. This compound exhibits a strong antioxidant activity within the membrane, suggesting that it may thereby have therapeutic effects [27]. High and diverse biological activities of 3-hydroxyflavone were demonstrated in a number of studies. 3-Hydroxyflavone administered to diabetic rats at a dose of 50 mg/kg body weight showed strong hypoglycemic and antidiabetic effects [28]. In research conducted using cancer cell lines, 3-hydroxyflavone showed the strongest antitumor activity among the nine test compounds, relative to the tumor lines A549 (lung cancer), B16F10 (murine melanoma), and K562 (leukemia). 3-Hydroxyflavone was not cytotoxic to the two normal cell lines. Described flavonol administered with imatinib mesylate, an anticancer drug showed activity of inhibiting leukemia cell lines resistant to imatinib [29]. 3-Hydroxyflavone exhibits strong activity against trypanosomiasis and leishmaniasis, parasitic diseases that afflict millions of people in Africa, Asia and South America. It was one of the most active compounds of the polyphenol group tested against parasites *Leishmania donovani* and *Trypanosoma brucei rhodesiense*, while showing a killing effect on *Trypanosoma cruzi* [30].

Often, the conversion of a hydroxyl group to a methoxy group results in a decrease in the biological activity of the flavonoid compound in vitro [7,31]. On the other hand methoxylated flavonoids have the ability to easily penetrate into the cells, which is a very desirable feature for creating new therapeutic agents. After transferring lung cell lines to the medium containing dimethoxyflavones, within five minutes the cells accumulated 30–50 times more test compounds than were present in the surrounding buffer. Research shows that these compounds easily penetrate the heart, lung, breast, or brain cells. The intracellular transport of 5,7-dimethoxyflavone was approximately 10-fold higher than that of chrysin (5,7-dihydroxyflavone). This may be due to the fact that methoxy derivatives of flavonoids are not so rapidly metabolized as their hydroxyl counterparts. As a result, their oral bioavailability exceeds the bioavailability of hydroxylated flavonoids. 5,7,4′-Trimethoxyflavone was approximately eight times more effective in inhibiting the propagation of oral squamous cell carcinoma than apigenin (5,7,4′-trihydroxyflavone). For MCF-7 breast cancer cell lines, 7-methoxyflavone had higher cytotoxicity than 7-hydroxyflavone [32].

Glycosylation alters the pharmacokinetic properties and selectivities of compounds. Doxorubicin, vancomycin, avermectin, and nystatin are drugs used in medicine which are biologically potent because of their sugar moieties [33]. Flavonols with sugar unit attached to the third position of the A ring are pharmacologically active (they possess anticarcinogenic, antiplatelet, antioxidant, antithrombic, antimutagenic, antimicrobial properties, as well as cardioprotective and vasoprotective activities) [34].

It is proved that quercetin glycosides have neuroprotective, cardioprotective, antioxidative, chemopreventive, and antiallergic properties [35]. Isoquercetin (3′,4′,5,7-tetrahydroxyflavone 3-*O-β*-d-glucopyranoside (**3b**) is a natural flavonoid found in vegetables, herbs, and flowers and it exerts multiple pharmacological effects against various diseases, i.e., it possesses antiviral, antidiabetic and antioxidant activities [36,37]. In many studies this glycoside shows higher biological activities than its parent aglycone—quercetin (**3**). Isoquercetin (**3b**) shows stronger antiobesity effect than quercetin (**3**), which was proved in a study on mice fed with high-fat diet [36]. In a study on male albino Wistar rats with induced diabetes mellitus isoquercetin (**3b**) showed antidiabetic effect *inter alia* by normalizing blood sugar and insulin levels [35]. Isoquercetin (**3b**) showed neuroprotective effect in primary culture of rat cortical and hippocampal neuronal cells from oxygen–glucose deprivation, followed by reperfusion-induced cell damage [37,38], as well as from tunicamycin-induced apoptosis in rat dorsal root ganglion neurons [39]. Neuroprotective effect of isoquercetin (**3b**) was also proved in vivo in rats that were subjected to middle cerebral artery occlusion and reperfusion injury [38].

Most researchers are certain that flavonoid glycosides before absorption in the digestive tract must be hydrolyzed by the intestinal microflora to the corresponding aglycones [24,40]. However, it has been shown that partial absorption of sugar derivatives of flavonoids is also possible. The glucose molecule attached to C-3 of quercetin increased the absorption of this glucoside in the small intestine to 52%, as compared to the 24% absorption of quercetin aglycone and 17% quercetin rutinoside [41]. Quercetin-3-*O*-glucoside has the ability to pass the enterocyte by the sodium dependent SGLUT1 transporter in the small intestine. Subsequently, the sugar unit is detached by lactase phloridzin hydrolase and aglycone passes the enterocyte via passive diffusion [9].

The high bioactivity of flavonoids that is shown in in vitro tests is often not confirmed by mammalian studies. These compounds are relatively rarely becoming components of pharmaceutical formulations. This is due to the low bioavailability of flavonoid compounds, *inter alia* due to poor water solubility [40,42]. As we have previously written [43,44], the microbiological synthesis of flavonoid glycosides is an excellent way to improve the physical properties of flavonoid compounds that are relevant for their use in the pharmaceutical industry.

In the present study, we report on biotransformations of four flavones: 3-hydroxyflavone, 3-methoxyflavone, quercetin and baicalein. As a result of the use of entomopathogenic filamentous fungi of the genus *Isaria* as biocatalysts we obtained seven flavonoid glycosides. We also provided an insight into the course of the formation of 4-*O*-methylglucopyranosides, which is a characteristic feature of the catalytic capacity of the described strains.

## 2. Results and Discussion

To obtain flavonoid glycosides we used four flavonoid substrates and three strains of entomopathogenic filamentous fungi, which were isolated from the environment. Detailed characteristics of the strains that we used can be found in our previous publications [44,45].

Below we present the results of biotransformations using *I. fumosorosea* KCH J2 strain as a biocatalyst, because the enzymatic system of this microorganism produced the most products with the highest yields. Experiments conducted on a larger scale enabled us to determine the chemical structures of products and their isolated yields. Three of obtained by us products (**1c**, **2a**, **4a**) have not been previously described in the literature.

### 2.1. Biotransformations of 3-Hydroxyflavone *(**1**)*

**Scheme 1:**
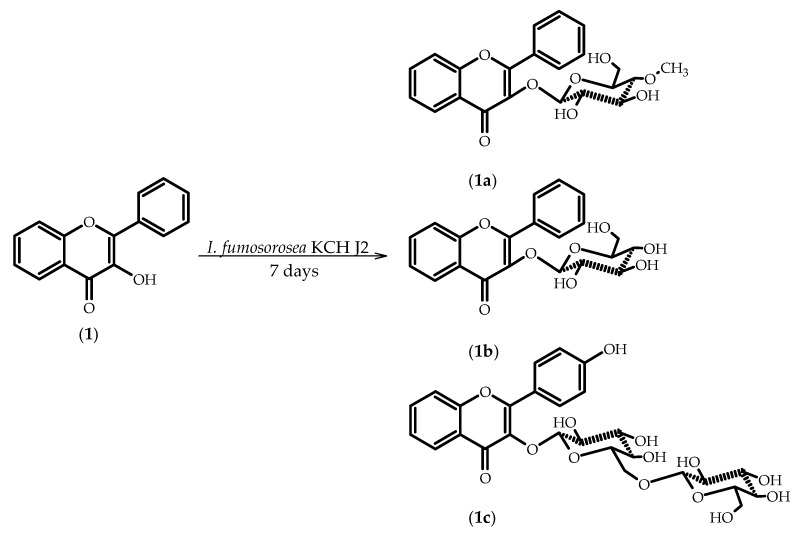
Microbial transformation of 3-hydroxyflavone (1) in *I. fumosorosea* KCH J2 culture.

As a result of 7-day biotransformation of 3-hydroxyflavone (**1**) in the culture of *I. fumosorosea* KCH J2 we obtained three products: flavone 3-*O*-*β*-d-(4′′-*O*-methyl)-glucopyranoside (**1a**), flavone 3-*O*-*β*-d-glucopyranoside (**1b**), and 3-*O*-[*β*-d-glucopyranosyl-(1→6)-*β*-d-glucopyranosyl]-4′-hydroxyflavone (**1c**) with the yields of 42.5%, 5%, and 3.5% respectively (Scheme 1). Compounds **1a** and **1b** were also formed when we used *I. farinosa* J1.4 and *I. farinosa* J1.6 as biocatalysts. In the culture of *I. farinosa* J1.4 (*I. farinosa* J1.6), we obtained **1a** with the yield of 18% (7.6%) and **1b** with the yield of 0.62% (1.2%).

The presence of a glucose unit in molecules **1a** (**1b**) is confirmed by five characteristic carbon signals observed in the region from about δ = 79.9 ppm (δ = 78.1 ppm) to about δ = 62 ppm in the ^13^C-NMR spectra, along with proton signals of δH ranging from about δ = 3.60 ppm to δ = 3.24 ppm (δ = 3.29 ppm) in the ^1^H-NMR spectra. Additionally, the attachment of a sugar unit to substrate **1** is confirmed by a one-proton doublet visible at δ = 5.28 ppm (δ = 5.29 ppm) in the ^1^H-NMR spectra, which are due to protons at the hemiacetal carbon atoms. The β-configuration of the glucose unit was proved by the coupling constant (*J* = 7.9 Hz/*J* = 7.7 Hz) for the anomeric proton. A three-proton singlet at δ = 3.55 ppm in the ^1^H-NMR of compound **1a** and the corresponding signal at about δ = 60.5 ppm in the ^13^C-NMR prove that one of the hydroxyl groups is methylated. *O*-methylation occurred in the C-4 hydroxyl group of the glucose moiety that was attached to compound **1a**, which was confirmed in the correlation spectra, where the proton signals due to -OCH_3_ are coupled with the signal of C-4 (about δ = 78.2 ppm) in the glucose unit. The sugar unit of compounds **1a** and **1b** is attached to C-3, because in the HMBC spectra the signal due to the proton at the hemiacetal carbon atom (δ = 5.28 ppm/δ = 5.29 ppm) is coupled with the C-3 signal (δ = 138.4 ppm/δ = 138.5 ppm). Chemical shifts of the other signals in the ^1^H and ^13^C-NMR spectra have only slightly changed, which indicates that the flavone skeleton remained intact. In the case of compound **1c** in the ^1^H-NMR spectrum, the presence of two glucose units is proved by ten carbon signals that can be observed in the region from δ =78.3 ppm to δ = 62.9 ppm in the ^13^C-NMR spectrum, along with proton signals of δH ranging from about δ = 4.02 ppm to δ = 3.20 ppm in the ^1^H-NMR spectrum. One-proton doublet visible at δ = 5.21 ppm in the ^1^H-NMR spectrum comes from first glucose unit, and the second one-proton doublet that can be observed in slightly higher field values at δ = 4.34 ppm comes from second glucose unit, proving the attachment of an disaccharide to the flavonoid skeleton. The coupling constants of both anomeric protons (*J* = 7.1 Hz/*J* = 7.9 Hz) prove the β-configuration of the both glucose units. The signal from first hemiacetal proton is coupled with the C-3 signal at δ = 137.9 ppm in the HMBC spectrum confirming the position of glycosylation is C-3. Hydroxylation at C-4′ in the case of **1c** is proved by the shift of signal from C-4′ of the substrates (**1**) that was visible at δ = 130.8 ppm in the ^13^C-NMR spectrum to δ = 161.1 ppm in the ^13^C-NMR spectrum of **1c**, indicating the attachment of an electronegative atom. There is no signal from the proton at C-4′ in the ^1^H-NMR spectrum. Additionally, two doublets from the protons at C-2′ and C-6′ (δ = 8.27 ppm) and C-3′ and C-5′ (δ = 7.04 ppm) can be observed.

There are only few literature reports on the biotransformations of 3-hydroxyflavone (**1**) in the cultures of microorganisms. Herath et al. using a strain of *Beauveria bassiana* (ATCC 13144) obtained 3,4’-dihdroxyflavone, flavone 3-*O*-*β*-d-(4′′-*O*-methyl)-glucopyranoside (**1a**), and two other minor metabolites. The biotransformation yield leading to the sugar derivative was relatively low at 5% [46]. Flavone 3-*O*-*β*-d-glucopyranoside (**1b**) with the yield of 22.5% was formed from 3-hydroxyflavone (**1**) with the use of *Cunninghamella echinulata* as biocatalyst [47].

### 2.2. Biotransformations of 3-Methoxyflavone *(**2**)*

**Scheme 2:**
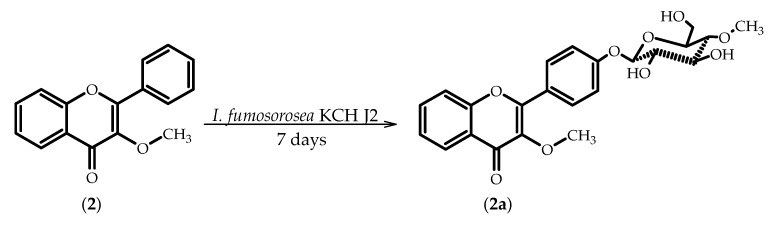
Microbial transformation of 3-methoxyflavone (2) in *I. fumosorosea* KCH J2 culture.

As a result of seven-day biotransformation of 3-methoxyflavone (**2**) in the culture of *I. fumosorosea* KCH J2 we obtained 3-methoxyflavone 4′-*O*-*β*-d-(4′′-*O*-methyl)-glucopyranoside (**2a**) with 29% yield (Scheme 2). Compound **2a** was also formed when we used *I. farinosa* J1.4 and *I. farinosa* J1.6 as biocatalysts with yields of 12.6% and 18.6%, respectively.

The presence of a glucose unit in molecule **2a** is confirmed by five characteristic carbon signals being observed in the region from δ = 80.1 ppm to δ = 62.1 ppm in the ^13^C-NMR spectrum, along with proton signals of δH ranging from about δ = 3.90 ppm to δ = 3.29 ppm in the ^1^H-NMR spectrum. The attachment of a sugar unit to substrate **2** is also confirmed by a one-proton doublet visible at δ = 5.13 ppm in the ^1^H-NMR spectrum of **2a,** which is due to protons at the hemiacetal carbon atoms. The β-configuration of the glucose unit was proved by the coupling constant (*J* = 7.4 Hz) for the anomeric proton. A three-proton singlet at δ = 3.62 ppm in the ^1^H-NMR and the corresponding signal at δ = 60.6 ppm in the ^13^C-NMR indicates the *O*-methylation of the hydroxyl group at C-4 of glucose, which is confirmed on the basis of the analysis of the correlation spectra of compound **2a**, where the proton signals that are due to -OCH_3_ (δ = 3.62 ppm) are coupled with the signal of C-4 (δ = 80.1 ppm) in the glucose unit. The sugar unit is attached to C-4′, because in the HMBC spectrum the signal due to the proton at the hemiacetal carbon atom (δ = 5.13 ppm) is coupled with the C-4′ signal (δ = 160.5 ppm). There is no signal from the proton at C-4′ in the ^1^H-NMR spectrum. Two two-proton doublets from the protons at C-2′ and C-6′ (δ = 8.16 ppm) and C-3′ and C-5′ (δ = 7.27 ppm) also indicate the substitution at C-4′.

The only example of biotransformation of 3-methoxyflavone (**2**) in the available literature is the transformation that we described in *Aspergillus niger* cultures. The first step was enzymatic oxygenation resulting in the formation of hemiacetal in position 3 of the A ring, which was then esterified with myristic acid. The final product has also an additional hydroxyl group in the 3’-position of the B-ring [48].

In biotransformations of flavonoid aglycons that do not contain hydroxyl groups performed in entomopathogenic filamentous fungi cultures, we often observe the attachment of the sugar unit to the 4′ position of the B ring. This was the case for the biotransformation of 6-methylflavone in *I. fumosorosea* KCH J2 culture. We obtained two products: 6-methylflavone 8-*O*-*β*-d-(4′′-*O*-methyl)-glucopyranoside and 6-methylflavone 4′-*O*-*β*-d-(4′′-*O*-methyl)-glucopyranoside [44].

### 2.3. Biotransformations of 3,3′,4′,5,7-Pentahydroxyflavone (Quercetin) *(**3**)*

**Scheme 3:**
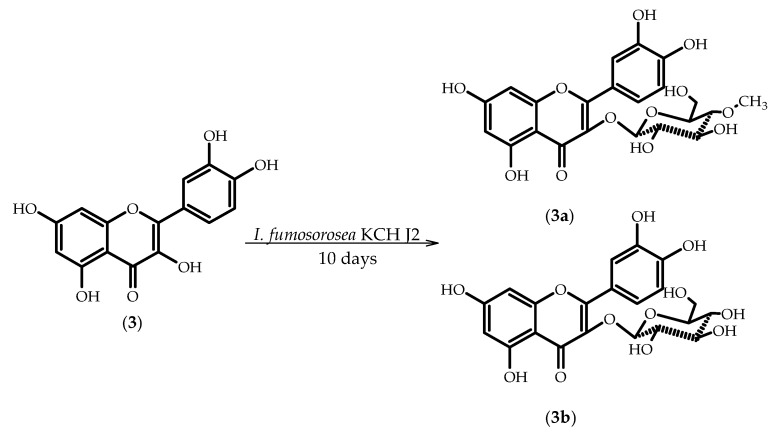
Microbial transformation 3,3′,4′,5,7-pentahydroxyflavone (quercetin) (3) in *I. fumosorosea* KCH J2 culture.

As a result of 10-day biotransformation of 3,3′,4′,5,7-pentahydroxyflavone (quercetin) (**3**) in the culture of *I. fumosorosea* KCH J2 we obtained 3′,4′,5,7-tetrahydroxyflavone 3-*O*-*β*-d-(4′′-*O*-methyl)-glucopyranoside (**3a**) and 3′,4′,5,7-tetrahydroxyflavone 3-*O*-*β*-d-glucopyranoside (isoquercetin) (**3b**), with the yields of 18.5% and 12%, respectively (Scheme 3).

The presence of a glucose unit in molecule **3a** (**3b**) is confirmed by five characteristic carbon signals that were observed in the region from δ = 79.8 ppm (δ = 78.1 ppm) to δ = 62.3 ppm (δ = 62.7 ppm) in the ^13^C-NMR spectra and proton signals from about δ = 3.73 ppm (δ = 3.78 ppm) to δ = 3.18 ppm (δ = 3.37 ppm) in the ^1^H-NMR spectra. Additionally, the attachment of a sugar unit to substrate **3** is confirmed by a one-proton doublet at δ = 5.27 ppm (δ = 5.30 ppm) in the ^1^H-NMR spectrum of **3a**, which is due to protons at the hemiacetal carbon atoms. The β-configuration of the glucose unit was proved by the coupling constant (*J* = 7.8 Hz/*J* = 7.4 Hz) for the anomeric proton. A three-proton singlet at δ = 3.57 ppm in the ^1^H-NMR and the corresponding signal at δ = 60.5 ppm in the ^13^C-NMR prove that one of the hydroxyl groups of glucose attached to **3a** has been methylated. *O*-methylation occurred in the C-4 hydroxyl group of the glucose moiety, which was confirmed in the correlation spectra of compound **3a**, where the proton signals due to -OCH_3_ (δ = 3.57 ppm) are coupled with the signal of C-4 (δ = 79.8 ppm) in the glucose unit. The sugar unit is attached to C-3, because, in the HMBC spectra, the signal due to the proton at the hemiacetal carbon atom (δ = 5.27 ppm/δ = 5.30 ppm) is coupled with the C-3 signal (δ = 135.6 ppm).

The formation of flavonoid derivatives with the attached 4-*O*-methylglucopyranose is a characteristic feature of entomopathogenic filamentous fungi of the genera *Beauveria* and *Isaria* [43,44,46,49,50,51]. Until recently, there was no research on the enzymes that are involved in this process. There was no clear answer to the question of whether the process is a one- or two-step. We wanted to check whether it is possible to attach the methyl group to glucose unit of isoquercetin (**3b**), therefore the obtained biotransformation product (**3b**) was used as a substrate for the *I. fumosorosea* KCH J2 strain. Samples in time were collected up to 14 days and were analyzed chromatographically. 3′,4′,5,7-Tetrahydroxyflavone 3-*O*-*β*-d-(4′′-*O*-methyl)-glucopyranoside (**3a**) did not form, therefore we believe that the synthesis of 4-*O*-methylglucopyranosides in cultures of the strains we describe occurs in one step and is competitive to glucosylation. This is consistent with the publication on enzymes that are involved in the formation of glycosides in *Beauveria bassiana* cultures. Chinese researchers used bioinformatic and genomic tools combined with heterologous expression to identify a glycosyltransferase–methyltransferase (GT–MT) gene pair that encodes a methylglucosylation functional module in described fungi [52].

In the literature, there are reports on obtaining quercetin (**3**) glycosides using biotransformations with whole microbial cells, enzymes, as well as via chemical synthesis.

Isoquercetin (**3b**) was obtained through biotransformation of quercetin (**3**) by *Gliocladium deliquescens* NRRL 1086. Transformation rapidly took place after quercetin (**3**) feeding; about 98% quercetin (**3**) were consumed within the initial 8 h. The yield of isoquercetin (**3b**) was 21.6% [53]. Sordon et al. in an extensive review on microbial glycosylation of flavonoids cited seven papers describing the microbiological transformations of quercetin (**3**) resulting in the formation of sugar derivatives [54]. In the culture of *Cunninghamella elegans* ATCC 9245 quercetin (**3**) was transformed into isoquercetin (**3b**), kaempferol 3-*O*-*β*-d-glucopyranoside, and isorhamnetin 3-*O*-*β*-d-glucopyranoside, with yields of 55.7%, 2.9%, and 4.9%, respectively. Biotransformation was performed for 4 days in 28 °C. Wild strain of *Streptomyces* M52104 isolated from soil gave quercetin monoglucuronides in positions 3, 7, 3′, and 4′. Quercetin 4′-O-β-d-glucuronide that was formed with the highest efficiency up to 50%. *Streptomyces rimosus* subsp. *rimosus* ATCC 10970 biotransformed quercetin (**3**) into quercetin 7-O-β-4’-deoxy-hex-4’-enopyranosiduronic acid within 96 h, at 28 °C. A wild strain of *Bacillus cereus* isolated from soil attached a glucose (unspecified α or β) unit into position 3 of quercetin. Biotransformation was performed for 24–36 h at 30 °C. The same product was formed with the use of genetically modified *Escherichia coli* with glucosyltransferase from *Oryza sativa*. Researchers observed the complete conversion of the substrate after 7 h of incubation at 30 °C. *Staphylococcus saprophyticus* CQ16 isolated from soil biotransformed quercetin (**3**) into 7-*O*-*β*-d-glucopyranoside with high yield of 96%. The most interesting from our point of view is biotransformation of quercetin (**3**) was performed with the use of entomopathogenic filamentous fungus *Beauveria bassiana* ATCC 7159. Process yielded in the formation of quercetin 7-*O*-*β*-d-(4′′-*O*-methyl)-glucopyranoside (87% yield) [54]. Interestingly, Penso et al. claims the formation of quercetin 7-*O*-*β*-d-glucopyranoside (without additional methyl group) with the use of the same strain (*Beauveria bassiana* ATCC 7159) [55]. There are no reports on the formation of quercetin 3-*O*-*β*-d-(4′′-*O*-methyl)-glucopyranoside (**3a**) in microbial cultures, however this compound was found in nature in an edible plant *Abelmoschus esculentus* L. [56].

In another research the litchi pericarp and its aqueous-organic extracted residues were fermented by *Aspergillus awamori*. The study identified that rutin, which is present in litchi pericarp, could be deglycosylated to form quercetin and quercetin-3-glucoside after the fermentation [57].

Quite a popular method of obtaining isoquercetin (**3b**) described in a number of publications is enzymatic deglycosylation of rutin. The purified α-L-rhamnosidase that was obtained from *Penicillium greoroseum* MTCC-9224 hydrolyzed rutin to isoquercetin (**3b**) and L-rhamnose. The pH and temperature optima of the enzyme were 6.5 and 57 °C, respectively. The release of isoquercetin (**3b**) was detected by thin-layer chromatography using starting compounds rutin and quercetin as reference compounds [58]. Brasilian researchers performed enzymatic hydrolysis of rutin while using α-L-rhamnosidases (hesperidinase from *Penicillium* sp. and naringinase from *Penicillium decumbens*) previously heated at 70 °C for 30 min to inactivate the undesirable *β*-d-glucosidase activity. Reaction mixtures were incubated for 2, 4, 8, and 12 h with shaking (130 rpm) at 40 °C. UPLC–MS analysis of hydrolyzed rutin after 2h-hesperidinase reaction confirmed a conversion of 48% of rutin into isoquercetin (**3b**), while after 4h-reaction, the conversion increased to 69.5% of isoquercetin (**3b**) and 7.5% of quercetin. More than 4 h hydrolysis did not increase isoquercetin (**3b**) production. Only 34.5% of isoquercetin (**3b**) was detected after 4 h of reaction using naringinase [59]. Wang et al. showed that the use of an ionic liquid (IL)-containing buffer system allows to increase the efficiency of the enzymatic conversion of rutin to isoquercetin (**3b**). The researchers used hesperidinase (containing both α-L-rhamnosidase and β-d-glucosidase activities, produced by *Aspergillus niger*) and a number of ionic liquid (IL)-containing buffer systems in various concentrations. With the use of [Bmim][BF_4_]: (1-Butyl-3-methylimidazolium tetrafluoroborate)- glycine-sodium hydroxide buffer (pH 9.0) (10:90, *v*/*v*) rutin conversion was 93.40% and the isoquercetin (**3b**) yield was 91.41% when compared to 55.80% and 39.23% obtained in aquenous buffer (pH 7.0), and there was no quercetin formation. The tests were performed with at 40 °C and 120 rpm [60].

It is also possible to obtain isoquercetin (**3b**) by chemical methods. Kajjout et al. described a procedure that allows for obtaining isoquercetin (**3b**) from quercetin (**3**) with 33% yield (calculated from the protected form of quercetin) [61].

### 2.4. Biotransformations of 5,6,7-Trihydroxyflavone (Baicalein) *(**4**)*

**Scheme 4:**
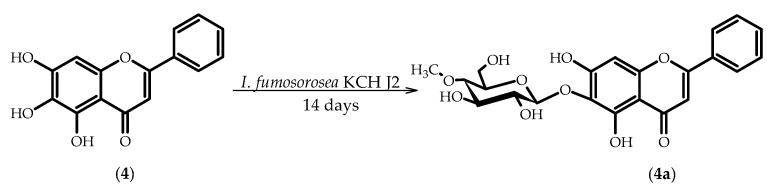
Microbial transformation of 5,6,7-Trihydroxyflavone (Baicalein) (4) in *I. fumosorosea* KCH J2 culture.

As a result of 14-day biotransformation of 5,6,7-Trihydroxyflavone (Baicalein) (**4**) in the culture of *I. fumosorosea* KCH J2, we obtained 5,7-dihydroxyflavone 6-*O*-*β*-d-(4′′-*O*-methyl)-glucopyranoside (**4a**) with 9.7% yield (Scheme 4).

The presence of a glucose unit in molecule **4a** is confirmed by five characteristic carbon signals observed in the region from δ = 61.8 ppm to δ = 79.7 ppm in the ^13^C-NMR spectra, along with proton signals of δH ranging from about δ = 3.74 ppm to δ = 3.20 ppm in the ^1^H-NMR spectra. Additionally, the attachment of a sugar unit to substrate **4** is confirmed by a one-proton doublet at δ = 4.63 ppm in the ^1^H-NMR spectrum of **4a** from protons at the hemiacetal carbon atoms. The β-configuration of the glucose unit was proved by the coupling constant (*J* = 7.5 Hz) for the anomeric proton. A three-proton singlet at δ = 3.54 ppm in the ^1^H-NMR and the corresponding signal at δ = 60.6 ppm in the ^13^C-NMR prove that one of the hydroxyl groups has been methylated. *O*-methylation occurred in the C-4 hydroxyl group of the glucose moiety, which was confirmed in the correlation spectra of compound **4a**, where the proton signals due to -OCH_3_ (δ = 3.54 ppm) are coupled with the signal of C-4 (δ = 79.7 ppm) in the glucose unit. The attachment of a sugar unit to C-6 is proved in the HMBC spectrum. The signal due to the proton at the hemiacetal carbon atom (δ = 4.63 ppm) is coupled with the C-6 signal (δ = 130.4 ppm).

Kostrzewa et. al carried out biotransformations of baicalein (**4**), resulting in the methylation of the hydroxyl group at the 6-position in the *Cryptosporiopsis radicicola* and *Chaetomium* sp. cultures, in the culture of *Penicillium chrysogenum*, another product was also observed with an additional methoxy group in the 4’ position of the B ring of baicalein (**4**) [62]. In a study conducted by another research team, baicalein (**4**) was also *O*-methylated to baicalein 6-methylether by *Streptomyces griseus* ATCC 13273 [63].

The flavonoid glycosides mainly occur as 3- or 7-*O*-glycosides [64] and microbial transformations lead most often to the formation of 7-*O*-glucosides and 3-*O*-glucosides, in the case of flavonols [54]. Baicalein 7-*O*-*β*-d-glucoside was formed in genetically modified culture of *Escherichia coli* with reconstructed uridine-diphosphate glucose (UDP-glucose) pathway cassette and a putative glycosyltransferase from *Arabidopsis thaliana* [65]. Baicalein 7-*O*-*β*-d-glucoside was also obtained with the use of UDP glucose: flavonoid 7-*O*-glucosyltransferase from hairy root cultures of *S. baicalensis* expressed and then isolated from *E. coli* [66]. Baicalein-6-α-glucoside was synthesized by using sucrose and the amylosucrase of *Deinococcus geothermalis* [67]. There are no reports on obtaining 5,7-dihydroxyflavone 6-*O*-*β*-d-(4′′-*O*-methyl)-glucopyranoside (baicalein 6-*O*-*β*-d-(4′′-*O*-methyl)-glucopyranoside **4a**).

## 3. Materials and Methods

### 3.1. Chemicals

The substrates for biotransformations, except for quercetin, were purchased from Sigma Chemical Company (St. Louis, MO, USA).

Quercetin was obtained via extraction with ethyl acetate from aqueous solution of effervescent tablets commercially available as a dietary supplement soothing allergy symptoms (Calcium DUO ALERGO + kwercetyna, Polski Lek, Wadowice, Poland).

#### 3.1.1. 3-Hydroxylavone (**1**)

C_15_H_10_O_3_, *t*_R_ 20.53; ^1^H-NMR, see Table 1; ^13^C-NMR, see Table 2.

#### 3.1.2. 3-Methoxyflavone (**2**)

C_16_H_12_O_3_, *t*_R_ 20.25; ^1^H-NMR, see Table 1; ^13^C-NMR, see Table 2.

#### 3.1.3. 3,3′,4′,5,7-Pentahydroxyflavone (Quercetin) (**3**)

C_15_H_10_O_7_, *t*_R_ 11.64; ^1^H-NMR, see Table 3; ^13^C-NMR, see Table 4.

#### 3.1.4. 5,6,7-Trihydroxyflavone (Baicalein) (**4**)

C_15_H_10_O_5_, *t*_R_ 13.46; ^1^H-NMR, see Table 3; ^13^C-NMR, see Table 4.

### 3.2. Microorganism

Full characteristics of fungal strains *I. fumosorosea* KCH J2 and *I. farinosa* J1.4 and J1.6 are included in our previous publications [44,45]. The microorganisms were maintained on potato slants at 4 °C and freshly subcultured before use in the experiments.

### 3.3. Analysis

The course of the biotransformation was evaluated by chromatographic methods (TLC, HPLC). TLC analysis was carried out while using TLC Silica gel 60/Kieselguhr F254 plates (Merck, Darmstadt, Germany). The developing system was a mixture of chloroform and methanol in the ratio of 9:1. Compounds were visualized using 5% aluminum chloride solution in ethanol. The plates were observed at two wavelengths: 254 and 365 nm.

HPLC analyses were performed with a Waters 2690 instrument equipped with a Waters 996 photodiode array detector, using an ODS 2 column (4.6 × 250 mm, Waters, Milford, MA, USA) and a Guard-Pak Inserts µBondapak C18 pre-column. Separation conditions were as follows: gradient elution, using 80% of acetonitrile in 4.5% formic acid solution (eluent A) and 4.5% formic acid (eluent B); flow, 1 mL/min; detection wavelength 280 nm; program: 0–7 min, 10% A 90% B; 7–10 min, 50% A 50% B; 10–13 min, 60% A 40% B; 13–15 min, 70% A 30% B; 15–20 min 80% A 20% B; 20–30 min 90% A 10% B; and, 30–40 min, 100% A.

Separation of the products obtained by the scale-up biotransformation was achieved while using 1000 μm preparative TLC silica gel plates (Analtech, Newark, DE, USA). After the development of the chromatograms in chloroform:methanol 9:1, compounds were extracted from the selected gel fragments using ethyl acetate (twice) and tetrahydrofuran (once). The extracts were combined and the solvents were removed while using a rotary evaporator.

NMR analysis was carried out using a Bruker Avance600 MHz NMR spectrometer (Bruker, Billerica, MA, USA) with an UltraShield Plusmagnet.

Mass spectra were obtained using high resolution electrospray ionization (ESI^+^-MS) (Waters LCT Premier XE mass spectrometer, Milford, MA, USA).

Optical rotation was measured while using digital polarimeter P-2000-Na (ABL&E-JASCO, Kraków, Poland).

### 3.4. Screening Procedure

In order to evaluate the biocatalytic capacity of the selected fungi strains small-scale biotransformations were conducted.

Experiments were carried out using Sabouraud medium (1% peptone, 3% glucose). The microorganism was transferred to a 300 mL flask containing 100 mL of the medium. Pre-incubation was carried out on a rotary shaker (140 rpm) at 25 °C for 72 h. Screening was carried out in 100 mL Erlenmeyer flasks containing 30 mL of Sabouraud liquid medium. The pre-grown culture (0.5 mL) was transferred to a flask and after 72-h incubation, 3 mg of the substrate dissolved in 0.5 mL of tetrahydrofuran was added. We used a separate flask for culture for each sample collection. The biotransformation was carried out under the same conditions as pre-incubation. After 4, 7, and 12 days of biotransformation the mixtures were extracted with 30 mL of ethyl acetate. The extracts were dried over MgSO_4_ (5 min), concentrated in vacuo, and analyzed by TLC and HPLC.

Stability of the substrate was evaluated under analogous conditions, without using a biocatalyst.

### 3.5. Scale-Up Biotransformations

In order to obtain biotransformation products in amounts that are sufficient to perform spectroscopic analyses scale-up biotransformations using *I. fumosorosea* KCH J2 as biocatalyst were performed.

Scale-up biotransformations were carried out in 2 L flasks containing 500 mL of the medium. The pre-incubation culture (1 mL) was transferred to the flask. After 72 h of incubation, 50 mg of the substrate dissolved in 1 mL of tetrahydrofuran was added. The scale-up biotransformation was carried out under the same conditions as the screening (140 rpm, 25 °C). After the complete consumption of the substrate different for each substrate used metabolites were extracted three times using each time 200 mL of ethyl acetate. The extracts were dried out while using MgSO_4_ and concentrated on a rotary evaporator. Product separation was carried out using preparative TLC plates. Product structure was determined by spectroscopic methods (^1^H-NMR, ^13^C-NMR, COSY, HMBC, HSQC).

The physical and spectral data of the products obtained are presented below (Table 1, Table 2, Table 3 and Table 4) (Supplementary Materials).

#### 3.5.1. Flavone 3-*O*-*β*-d-(4′′-*O*-Methyl)-glucopyranoside (**1a**)

C_22_H_22_O_8_, *t*_R_ 12.02, ${\left[\alpha \right]}_{D}^{20}=$ −30.8°, ^1^H-NMR, see Table 1; ^13^C-NMR, see Table 2.

HRESI-MS [M + H^+^] was calculated/found (*m*/*z* 414.4053/414.1307).

#### 3.5.2. Flavone 3-*O*-*β*-d-Glucopyranoside (**1b**)

C_21_H_20_O_8_, *t*_R_ 11.132, ${\left[\alpha \right]}_{D}^{20}=$ −49.1°, ^1^H-NMR, see Table 1; ^13^C-NMR, see Table 2.

HRESI-MS [M + H^+^] was calculated/found (*m*/*z* 400.3787/400.1117).

#### 3.5.3. 3-*O*-[*β*-d-Glucopyranosyl-(1→6)-*β*-d-glucopyranosyl]-4’-hydroxyflavone (**1c**)

C_27_H_30_O_14_, *t*_R_ 10.48, ${\left[\alpha \right]}_{D}^{20}=$ −80.7°, ^1^H-NMR, see Table 1; ^13^C-NMR, see Table 2.

HRESI-MS [M + H^+^] was calculated/found (*m*/*z* 578.5187/578.0050).

#### 3.5.4. 3-Methoxyflavone 4′-*O*-*β*-d-(4′′-*O*-methyl)-glucopyranoside (**2a**)

C_23_H_24_O_9_, *t*_R_ 10.89, ${\left[\alpha \right]}_{D}^{20}=$ −34.0°, ^1^H-NMR, see Table 1; ^13^C-NMR, see Table 2.

HRESI-MS [M + H^+^] was calculated/found (*m*/*z* 444.4313/444.1374).

#### 3.5.5. 3′,4′,5,7-Tetrahydroxyflavone 3-*O*-*β*-d-(4′′-*O*-methyl)-glucopyranoside (**3a**)

C_22_H_22_O_12_, *t*_R_ 11.10, ${\left[\alpha \right]}_{D}^{20}=$ −16.3°, ^1^H-NMR, see Table 3; ^13^C-NMR, see Table 4.

HRESI-MS [M + H^+^] was calculated/found (*m*/*z* 478.4029/478.1021).

#### 3.5.6. 3′,4′,5,7-Tetrahydroxyflavone 3-*O*-*β*-d-glucopyranoside (isoquercetin) (**3b**)

C_21_H_20_O_12_, *t*_R_ 10.68, ${\left[\alpha \right]}_{D}^{20}=$ −18.0°, ^1^H-NMR, see Table 3; ^13^C-NMR, see Table 4.

HRESI-MS [M + H^+^] was calculated/found (*m*/*z* 464.3763/464.0867).

#### 3.5.7. 5,7-Dihydroxyflavone 6-*O*-*β*-d-(4′′-*O*-methyl)-glucopyranoside (**4a**)

C_22_H_22_O_10_, *t*_R_ 10.00, ${\left[\alpha \right]}_{D}^{20}=$ −85.8°, ^1^H-NMR, see Table 3; ^13^C-NMR, see Table 4.

HRESI-MS [M + H^+^] was calculated/found (*m*/*z* 446.4041/446.1118).

## 4. Conclusions

We evaluated the biocatalytic capacity of three strains of entomopathogenic filamentous fungi of the genus *Isaria* towards substituted flavones. We received the following sugar derivatives: from 3-hydroxyflavone (**1**)-flavone 3-*O*-*β*-d-(4′′-*O*-methyl)-glucopyranoside (**1a**), flavone 3-*O*-*β*-d-glucopyranoside (**1b**), and 3-*O*-[*β*-d-glucopyranosyl-(1→6)-*β*-d-glucopyranosyl]-4′-hydroxyflavone (**1c**), from 3-methoxyflavone (**2**)-3-methoxyflavone 4′-*O*-*β*-d-(4′′-*O*-methyl)-glucopyranoside (**2a**), from quercetin (**3**)-3′,4′,5,7-tetrahydroxyflavone 3-*O*-*β*-d-(4′′-*O*-methyl)-glucopyranoside (**3a**) and 3′,4′,5,7-tetrahydroxyflavone 3-*O*-*β*-d-glucopyranoside (isoquercetin) (**3b**), and from baicalein (**4**)-5,7-dihydroxyflavone 6-*O*-*β*-d-(4′′-*O*-methyl)-glucopyranoside (**4a**). Our research has enabled the extension of the flavonoid glycoside library with compounds that are not found in nature. Three derivatives (**1c**, **2a**, **4a**) have not been described in the literature so far. In addition, we have shown that 4-*O*-methylglucopyranose is being attached to the flavonoid aglycon in a one-step process. The substrates and products that are presented in this paper can be used in biological assays to compare activity and bioavailability of aglycone/glycoside pairs of flavonoids, and in the future may become components of new pharmaceutical and cosmetic preparations or food additives.

## Figures and Tables

**Table 1:** ^1^H-NMR shifts (δ) of 3-hydroxyflavone (**1**) and 3-methoxyflavone (**2**) and products of their biotransformations (**1a**, **1b**, **1c**, **2a**) in Acetone-d_6_, 600 MHz (Supplementary Materials).

Proton	Compound					
	1	1a	1b	1c	2	2a
H-3	-	-	-	-	-	-
H-5	8.22 (dd) *J*_5,6_ = 7.9 Hz, *J*_5,7_ = 1.6 Hz	8.23 (d) *J*_5,6_ = 8.0 Hz	8.24 (d) *J*_5,6_ = 8.0 Hz	8.22 (d) *J*_5,6_ = 8.0 Hz	8.21 (dd) *J*_5,6_ = 8.1 Hz, *J*_5,7_ = 1.9 Hz	8.19 (d) *J*_5,6_ = 7.7 Hz
H-6	7.55 (m)	7.57 (t) *J* = 7.5 Hz	7.58 (m)	7.56 (t) *J* = 7.5 Hz	7.53 (ddd) *J*_6,5_ = 8.0 Hz, *J*_6,7_ = 7.1 Hz, *J* = 0.9 Hz	7.51 (t) *J* = 7.4 Hz
H-7	7.87 (ddd) *J*_7,5_ = 1.7 Hz, *J*_7,6_ = 7.0 Hz, *J*_7,8_ = 8.7 Hz	7.90 (t) *J*_7,8_ = 7.7 Hz	7.91 (t) *J*_7,8_ = 7.8 Hz	7.88 (t) *J*_7,8_ = 7.6 Hz	7.85 (ddd) *J*_7,5_ = 1.7 Hz, *J*_7,6_ = 7.1 Hz, *J*_7,8_ = 8.7 Hz	7.83 (t) *J* = 7.8 Hz
H-8	7.81 (d) *J*_8,7_ = 8.3 Hz	7.78 (d) *J*_8,7_ = 8.5 Hz	7.79 (d) *J*_8,7_ = 8.4 Hz	7.77 (d) *J*_8,7_ = 8.5 Hz	7.74 (d) *J*_8,7_ = 8.3 Hz	7.73 (d) *J*_8,7_ = 8.4 Hz
H-2′	8.37 (m)	8.29 (dd) *J* = 5.9 Hz, *J* = 2.4 Hz	8.29 (m)	8.27 (d) *J*_2’,3’_ = 8.4 Hz	8.17 (m)	8.16 (d) *J*_2’,3’_ = 8.4 Hz
H-3′	7.63 (m)	7.58 (m)	7.58 (m)	7.04 (d) *J*_3’,2’_ = 8.2 Hz	7.62 (m)	7.27 (d) *J*_3’,2’_ = 8.2 Hz
H-4′	7.55 (m)	7.58 (m)	7.58 (m)	-	7.62 (m)	-
H-5′	7.63 (m)	7.58 (m)	7.58 (m)	7.04 (d) *J*_5’,6’_ = 8.2 Hz	7.62 (m)	7.27 (d) *J*_5’,6’_ = 8.2 Hz
H-6′	8.37 (m)	8.29 (dd) *J* = 5.9 Hz, *J* = 2.4 Hz	8.29 (m)	8.27 (d) *J*_6’,5’_ = 8.4 Hz	8.17 (m)	8.16 (d) *J*_6’,5’_ = 8.4 Hz
H-1′′	-	5.28 (d) *J* = 7.9 Hz	5.29 (d) *J* = 7.7 Hz	5.21 (d) *J* = 7.1 Hz	-	5.13 (d) *J* = 7.4 Hz
H-2′′	-	3.44 (t) *J* = 8.5 Hz	3.44 (t) *J* = 8.4 Hz	3.48 (s)	-	3.56 (m)
H-3′′	-	3.63 (t) *J* = 8.9 Hz	3.50 (t) *J* = 9.1 Hz	3.51 (d) *J* = 8.7 Hz	-	3.71 (t)
H-4′′	-	3.15 (t) *J* = 9.3 Hz	3.38 (t) *J* = 9.2 Hz	3.41 (t) *J* = 9.6 Hz	-	3.29 (t)
H-5′′	-	3.24 (m)	3.29 (m)	3.33–3.38 (m)	-	3.56 (m)
H-6′′	-	3.58 (m) 3.52 (m)	3.65 (d) *J* = 12.1 Hz 3.52 (m)	3.81–3.85 (m) 3.98–4.02 (m)	-	3.90 (m) 3.76 (m)
H-1′′′	-	-	-	4.34 (d) *J* = 7.9 Hz	-	-
H-2′′′	-	-	-	3.20 (m)	-	-
H-3′′′	-	-	-	3.43 (d) *J* = 8.4 Hz	-	-
H-4′′′	-	-	-	3.33–3.38 (m)	-	-
H-5′′′	-	-	-	3.30 (m)	-	-
H-6′′′	-	-	-	3.69 (m) 3.86–3.91 (m)	-	-
C-4′′-OCH_3_	-	3.55 (s)	-	-	-	3.62 (s)
C3-OH	8.26 (s)	-	-	-	-	
C3-OCH_3_	-	-	-	-	3.95 (s)	3.93 (s)

**Table 2:** ^13^C-NMR shifts (δ) of 3-hydroxyflavone (**1**) and 3-methoxyflavone (**2**) and products of their biotransformations (**1a**, **1b**, **1c**, **2a**) in Acetone-d_6_, 151 MHz (Supplementary Materials).

Carbon	Compound					
	1	1a	1b	1c	2	2a
C-2	145.5	158.3	158.4	158.6	156.1	155.8
C-3	139.8	138.4	138.5	137.9	142.2	141.6
C-4	173.9	175.7	175.7	175.6	175.1	174.9
C-4a	122.0	124.5	124.6	126.2	125.1	125.1
C-5	125.9	126.2	126.3	125.9	126.1	126.0
C-6	125.4	126.0	126.1	124.5	125.7	125.6
C-7	134.7	135.1	135.1	134.9	134.6	134.4
C-8	119.3	119.3	119.3	119.1	119.2	119.1
C-8a	156.3	156.3	156.4	156.2	156.2	156.1
C-1′	132.5	131.9	132.0	122.8	132.0	125.5
C-2′	128.6	130.3	130.3	132.3	129.3	130.9
C-3′	129.4	129.0	129.0	115.9	129.4	117.2
C-4′	130.8	131.7	131.7	161.1	131.5	160.5
C-5′	129.4	129.0	129.0	115.9	129.4	117.2
C-6′	128.6	130.3	130.3	132.3	129.3	130.9
C-1′′	-	104.5	104.9	105.6	-	101.2
C-2′′	-	75.5	75.3	75.4	-	74.9
C-3′′	-	79.9	78.1	78.3	-	78.0
C-4′′	-	78.2	71.2	71.2	-	80.1
C-5′′	-	77.2	77.9	77.6	-	77.2
C-6′′	-	62.2	62.8	70.0	-	62.1
C-1′′′	-	-	-	104.8	-	-
C-2′′′	-	-	-	74.8	-	-
C-3′′′	-	-	-	77.9	-	-
C-4′′′	-	-	-	71.5	-	-
C-5′′′	-	-	-	77.9	-	-
C-6′′′	-	-	-	62.9	-	-
C-4′′-OCH_3_	-	60.5	-	-	-	60.6
C3-OCH_3_	-	-	-	-	60.2	60.0

**Table 3:** ^1^H-NMR shifts (δ) of quercetin (**3**)^1^ and baicalein (**4**)^2^ and products of their biotransformations (**3a**^1^, **3b**^1^, **4a**^2^) in Acetone-d_6_^1^/Tetrahydrofuran-d_8_^2^, 600 MHz (Supplementary Materials).

Proton	Compound				
	3	3a	3b	4	4a
H-3	-	-	-	6.74 (s)	6.79 (s)
H-5	-	-	-	-	-
H-6	6.30 (d) *J_6,8_* = 2.0 Hz	6.32 (d) *J*_6,8_ = 2.0 Hz	6.32 (d) *J*_6,8_ = 2.0 Hz	-	-
H-7	-			-	-
H-8	6.56 (d) *J*_8,6_ = 2.1 Hz	6.55 (d) *J*_8,6_ = 2.1 Hz	6.55 (d) *J*_8,6_ = 2.1 Hz	6.53 (s)	6.55 (s)
H-2′	7.86 (d) *J*_2’,6’_ = 2.2 Hz	8.04 (d) *J*_2’,6’_ =2.1 Hz	8.04 (d) *J*_2’,6’_ = 2.1 Hz	7.99 (m)	8.01 (m)
H-3′	-	-	-	7.52 (m)	7.53 (m)
H-4′	-	-	-	7.52 (m)	7.53 (m)
H-5′	7.03 (d) *J*_5’,6’_ = 8.5 Hz	7.00 (d) *J*_5’,6’_ = 8.4 Hz	6.99 (d) *J*_5’,6’_ = 8.4 Hz	7.52 (m)	7.53 (m)
H-6′	7.74 (dd) *J_6’,2’_* = 2.2 Hz, *J*_6’,5’_ = 8.5 Hz	7.64 (dd) *J_6’,2’_* = 2.2 Hz, *J*_6’,5’_ = 8.4 Hz	7.62 (dd) *J*_6’,2’_ = 2.2 Hz, *J*_6’,5’_ = 8.4 Hz	7.99 (m)	8.01 (m)
H-1′′	-	5.27 (d) *J* = 7.8 Hz	5.30 (d) *J* = 7.4 Hz	-	4.63 (d) *J* = 7.4 Hz
H-2′′	-	3.50 (t) *J* = 8.1 Hz	3.52 (m)	-	3.47 (m)
H-3′′	-	3.63 (m)	3.52 (m)	-	3.47 (m)
H-4′′	-	3.18 (m)	3.44 (t) *J* =8.8 Hz	-	3.25 (m)
H-5′′	-	3.34 (m)	3.37 (m)	-	3.20 (ddd) *J* = 9.6, 3.6, 2.0 Hz
H-6′′	-	3.73 (d) *J* = 12.0 Hz 3.64 (m)	3.78 (d) *J* = 10.7 Hz 3.64 (m)	-	3.74 (d) *J* = 9.8 Hz 3.62 (m)
C-4′′-OCH_3_	-	3.57 (s)	-	-	3.54 (s)
C3-OH	8.05 (s)	-	-	-	-
C5-OH	12.21 (s)	12.34	-	12.78 (s)	13.16 (s)
C6-OH			-	8.46 (s)	-
C7-OH	9.78 (s)	9.82 (s)	-	7.22 (s)	9.51 (s)
C3′-OH	8.38 (s)	8.48 (s)	-	-	-
C4′-OH	8.61 (s)	8.52 (s)	-	-	-

**Table 4:** ^13^C-NMR shifts (δ) of quercetin (**3**)^1^ and baicalein (**4**)^2^ and products of their biotransformations (**3a**^1^, **3b**^1^, **4a**^2^) in Acetone-d_6_^1^/Tetrahydrofuran-d_8_^2^, 151 MHz (Supplementary Materials).

Carbon	Compound				
	3	3a	3b	4	4a
C-2	146.9	158.8	158.8	164.3	164.7
C-3	136.8	135.6	135.6	105.6	105.8
C-4	176.6	179.2	179.2	183.4	183.5
C-4a	104.1	105.5	105.4	105.6	105.8
C-5	162.3	158.0	158.0	147.9	154.8
C-6	99.2	94.7	94.7	130.3	130.4
C-7	165.0	165.3	165.4	154.4	158.9	
C-8	94.5	99.8	99.7	94.6	95.0
C-8a	157.8	162.9	162.9	151.6	155.0
C-1′	123.8	122.7	122.7	132.9	132.6
C-2′	115.8	118.0	117.9	127.1	127.2
C-3′	145.8	145.3	145.3	129.8	129.9
C-4′	148.4	149.3	149.3	132.3	132.6
C-5′	116.2	115.9	115.8	129.8	129.9
C-6′	121.5	122.8	122.6	127.1	127.2
C-1′′	-	104.8	105.0	-	107.3
C-2′′	-	75.7	75.5	-	75.5
C-3′′	-	78.2	78.1	-	77.8
C-4′′	-	79.8	70.9	-	79.7
C-5′′	-	77.0	77.8	-	77.8
C-6′′	-	62.3	62.7	-	61.8
C-4′′-OCH_3_	-	60.5	-	-	60.6

## Supplementary Materials

The following are available online.
