# Supplementary material for: Glycosylation of 3-Hydroxyflavone, 3-Methoxyflavone, Quercetin and Baicalein in Fungal Cultures of the Genus Isaria
Source: Molecules. 2018 Sep 27;23(10):2477. doi: 10.3390/molecules23102477 (PMC6222337; doi:10.3390/molecules23102477)
Supplement: Supplementary file 1 [file molecules-23-02477-s001.pdf]

Supplementary data

Glycosylation of 3-hydroxyflavone, 3-methoxyflavone, quercetin and baicalein in fungal cultures of the genus *Isaria*

Monika Dymarska*, Tomasz Janeczko and Edyta Kostrzewa - Susłow
*Correspondence: monika.dymarska@gmail.com
Department of Chemistry, Faculty of Biotechnology and Food Science, Wrocław University of Environmental and Life Sciences, Wrocław, Poland

Content

Figure S1. ^{1}H NMR spectrum of 3-hydroxyflavone (1) (Acetone-d_6, 600 MHz)
Figure S2. ^{13}C NMR spectrum of 3-hydroxyflavone (1) (Acetone-d_6, 151 MHz)
Figure S3. HSQC NMR spectrum of 3-hydroxyflavone (1) (Acetone-d_6, 151 MHz)
Figure S4. HMBC NMR spectrum of 3-hydroxyflavone (1) (Acetone-d_6, 151 MHz)
Figure S5. ^{1}H NMR spectrum of flavone 3-*O*-*β*-D-(4″-*O*-methyl)-glucopyranoside (1a) (Acetone-d_6, 600 MHz)
Figure S6. ^{1}H NMR spectrum of flavone 3-*O*-*β*-D-(4″-*O*-methyl)-glucopyranoside (1a) (Acetone-d_6, 600 MHz)
Figure S7. ^{13}C NMR spectrum of flavone 3-*O*-*β*-D-(4″-*O*-methyl)-glucopyranoside (1a) (Acetone-d_6, 151 MHz)
Figure S8. HSQC NMR spectrum of flavone 3-*O*-*β*-D-(4″-*O*-methyl)-glucopyranoside (1a) (Acetone-d_6, 151 MHz)
Figure S9. HMBC NMR spectrum of flavone 3-*O*-*β*-D-(4″-*O*-methyl)-glucopyranoside (1a) (Acetone-d_6, 151 MHz)
Figure S10. ^{1}H NMR spectrum of flavone 3-*O*-*β*-D-glucopyranoside (1b) (Acetone-d_6, 600 MHz)
Figure S11. ^{1}H NMR spectrum of flavone 3-*O*-*β*-D-glucopyranoside (1b) (Acetone-d6, 600 MHz)
Figure S12. ^{13}C NMR spectrum of flavone 3-*O*-*β*-D-glucopyranoside (1b) (Acetone-d_6, 151 MHz)
Figure S13. HSQC NMR spectrum of flavone 3-*O*-*β*-D-glucopyranoside (1b) (Acetone-d_6, 151 MHz)
Figure S14. HMBC NMR spectrum of flavone 3-*O*-*β*-D-glucopyranoside (1b) (Acetone-d_6, 151 MHz)
Figure S15. ^{1}H NMR spectrum of 3-*O*-[*β*-D-glucopyranosyl-(1→6)-*β*-D-glucopyranosyl]-4′-hydroxyflavone (1c) (Acetone-d_6, 600 MHz)

Figure S16. ^{1}H NMR spectrum of 3-*O*-[*β*-D-glucopyranosyl-(1→6)-*β*-D-glucopyranosyl]-4′-hydroxyflavone (1c) (Acetone-d_6, 600 MHz)

Figure S17. ^{13}C NMR spectrum of 3-*O*-[*β*-D-glucopyranosyl-(1→6)-*β*-D-glucopyranosyl]-4′-hydroxyflavone (Acetone-d_6, 151 MHz)

Figure S18. HSQC NMR spectrum of 3-*O*-[*β*-D-glucopyranosyl-(1→6)-*β*-D-glucopyranosyl]-4′-hydroxyflavone (Acetone-d_6, 151 MHz)

Figure S19. HMBC NMR spectrum of 3-*O*-[*β*-D-glucopyranosyl-(1→6)-*β*-D-glucopyranosyl]-4′-hydroxyflavone (Acetone-d_6, 151 MHz)

Figure S20. ^{1}H NMR spectrum of 3-methoxyflavone (2) (Acetone-d_6, 600 MHz)

Figure S21. ^{13}C NMR spectrum of 3-methoxyflavone (2) (Acetone-d_6, 151 MHz)

Figure S22. HSQC NMR spectrum of 3-methoxyflavone (2) (Acetone-d_6, 151 MHz)

Figure S23. HMBC NMR spectrum of 3-methoxyflavone (2) (Acetone-d_6, 151 MHz)

Figure S24. ^{1}H NMR spectrum of 3-methoxyflavone 4′-*O*-*β*-D-(4″-*O*-methyl)-glucopyranoside (2a) (Acetone-d_6, 600 MHz)

Figure S25. ^{1}H NMR spectrum of 3-methoxyflavone 4′-*O*-*β*-D-(4″-*O*-methyl)-glucopyranoside (2a) (Acetone-d6, 600 MHz)

Figure S26. ^{13}C NMR spectrum of 3-methoxyflavone 4′-*O*-*β*-D-(4″-*O*-methyl)-glucopyranoside (2a) (Acetone-d_6, 151 MHz)

Figure S27. HSQC NMR spectrum of 3-methoxyflavone 4′-*O*-*β*-D-(4″-*O*-methyl)-glucopyranoside (2a) (Acetone-d_6, 151 MHz)

Figure S28. HMBC NMR spectrum of 3-methoxyflavone 4′-*O*-*β*-D-(4″-*O*-methyl)-glucopyranoside (2a) (Acetone-d_6, 151 MHz)

Figure S29. ^{1}H NMR spectrum of 3,3′,4′,5,7-Pentahydroxyflavone (Quercetin) (3) (Acetone-d_6, 600 MHz)

Figure S30. ^{1}H NMR spectrum of 3,3′,4′,5,7-Pentahydroxyflavone (Quercetin) (3) (Acetone-d_6, 600 MHz

Figure S31. ^{13}C NMR spectrum of 3,3′,4′,5,7-Pentahydroxyflavone (Quercetin) (3) (Acetone-d_6, 151 MHz)

Figure S32. HSQC NMR spectrum of 3,3′,4′,5,7-Pentahydroxyflavone (Quercetin) (3) (Acetone-d_6, 151 MHz)

Figure S33. HMBC NMR spectrum of 3,3′,4′,5,7-Pentahydroxyflavone (Quercetin) (3) (Acetone-d_6, 151 MHz)

Figure S34. ^{1}H NMR spectrum of 3′,4′,5,7-tetrahydroxyflavone 3-*O*-*β*-D-(4″-*O*-methyl)-glucopyranoside (3a) (Acetone-d_6, 600 MHz)

Figure S35. ^{1}H NMR spectrum of 3′,4′,5,7-tetrahydroxyflavone 3-*O*-*β*-D-(4″-*O*-methyl)-glucopyranoside (3a) (Acetone-d_6, 600 MHz)

Figure S36. ^{13}C NMR spectrum of 3′,4′,5,7-tetrahydroxyflavone 3-*O*-*β*-D-(4″-*O*-methyl)-glucopyranoside (3a) (Acetone-d_6, 151 MHz)

Figure S37. HSQC NMR spectrum of 3′,4′,5,7-tetrahydroxyflavone 3-*O*-*β*-D-(4″-*O*-methyl)-glucopyranoside (3a) (Acetone-d_6, 151 MHz)

Figure S38. HMBC NMR spectrum of 3′,4′,5,7-tetrahydroxyflavone 3-*O*-*β*-D-(4″-*O*-methyl)-glucopyranoside (3a) (Acetone-d_6, 151 MHz)

Figure S39. ^{1}H NMR spectrum of 3′,4′,5,7-tetrahydroxyflavone 3-*O*-*β*-D-glucopyranoside (isoquercetin) (3b) (Acetone-d_6, 600 MHz)

Figure S40. ^{1}H NMR spectrum of 3′,4′,5,7-tetrahydroxyflavone 3-*O*-*β*-D-glucopyranoside (isoquercetin) (3b) (Acetone-d_6, 600 MHz)

Figure S41. ^{13}C NMR spectrum of 3′,4′,5,7-tetrahydroxyflavone 3-*O*-*β*-D-glucopyranoside (isoquercetin) (3b) (Acetone-d_6, 151 MHz)

Figure S42. HSQC NMR spectrum of 3′,4′,5,7-tetrahydroxyflavone 3-*O*-*β*-D-glucopyranoside (isoquercetin) (3b) (Acetone-d_6, 151 MHz)

Figure S43. HMBC NMR spectrum of 3′,4′,5,7-tetrahydroxyflavone 3-*O*-*β*-D-glucopyranoside (isoquercetin) (3b) (Acetone-d_6, 151 MHz)

Figure S44. ^{1}H NMR spectrum of 5,6,7-Trihydroxyflavone (Baicalein) (4) (Tetrahydrofuran-d_8, 600 MHz)

Figure S45. ^{13}C NMR spectrum of 5,6,7-Trihydroxyflavone (Baicalein) (4) (Tetrahydrofuran-d_8, 151 MHz)

Figure S46. HSQC NMR spectrum of 5,6,7-Trihydroxyflavone (Baicalein) (4) (Tetrahydrofuran-d_8, 151 MHz)

Figure S47. HMBC NMR spectrum of 5,6,7-Trihydroxyflavone (Baicalein) (4) (Tetrahydrofuran-d_8, 151 MHz)

Figure S48. ^{1}H NMR spectrum of 5,7-dihydroxyflavone 6-*O*-*β*-D-(4″-*O*-methyl)-glucopyranoside (4a) (Tetrahydrofuran-d_8, 600 MHz)

Figure S49. ^{1}H NMR spectrum of 5,7-dihydroxyflavone 6-*O*-*β*-D-(4″-*O*-methyl)-glucopyranoside (4a) (Tetrahydrofuran-d_8, 600 MHz)

Figure S50. ^{13}C NMR spectrum of 5,7-dihydroxyflavone 6-*O*- *β* -D-(4″-*O*-methyl)-glucopyranoside (4a) (Tetrahydrofuran-d_8, 151 MHz)

Figure S51. HSQC NMR spectrum of 5,7-dihydroxyflavone 6-*O*- *β* -D-(4″-*O*-methyl)-glucopyranoside (4a) (Tetrahydrofuran-d_8, 151 MHz)

Figure S52. HMBC NMR spectrum of 5,7-dihydroxyflavone 6-*O*- *β* -D-(4″-*O*-methyl)-glucopyranoside (4a) (Tetrahydrofuran-d_8, 151 MHz)

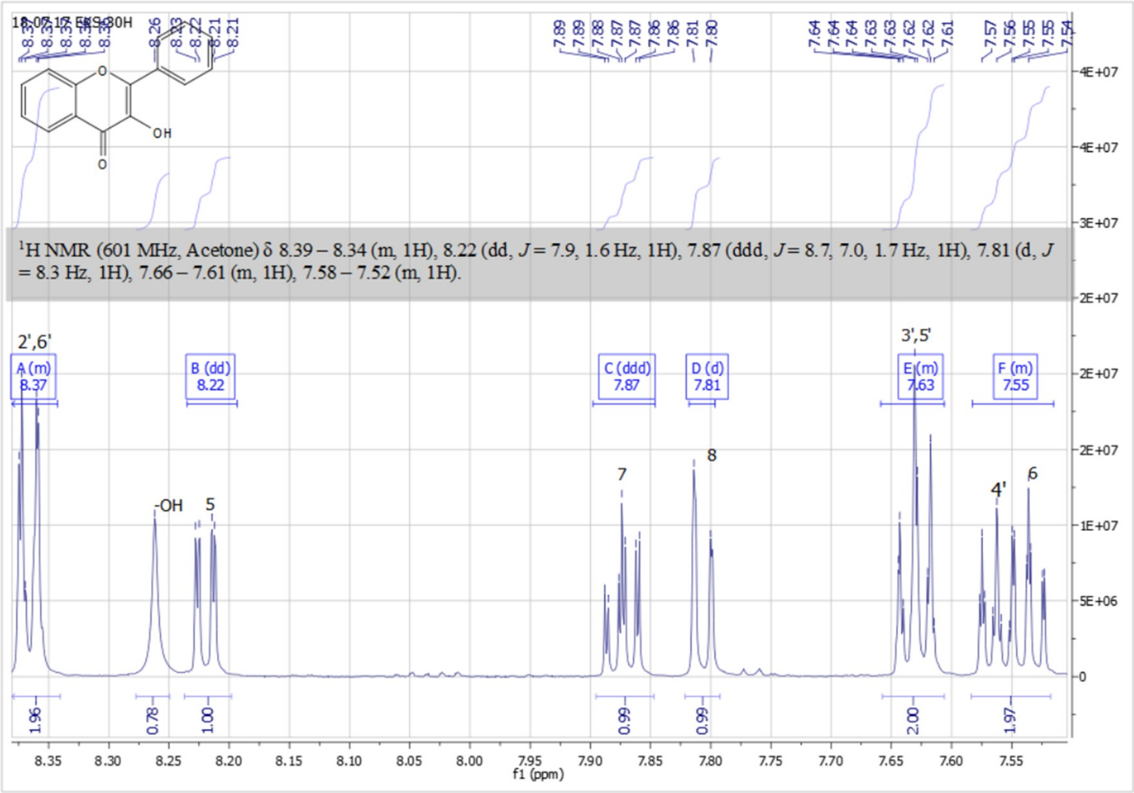

Figure S1. ^{1}H NMR spectrum of 3-hydroxyflavone (1) (Acetone-d_6, 600 MHz)

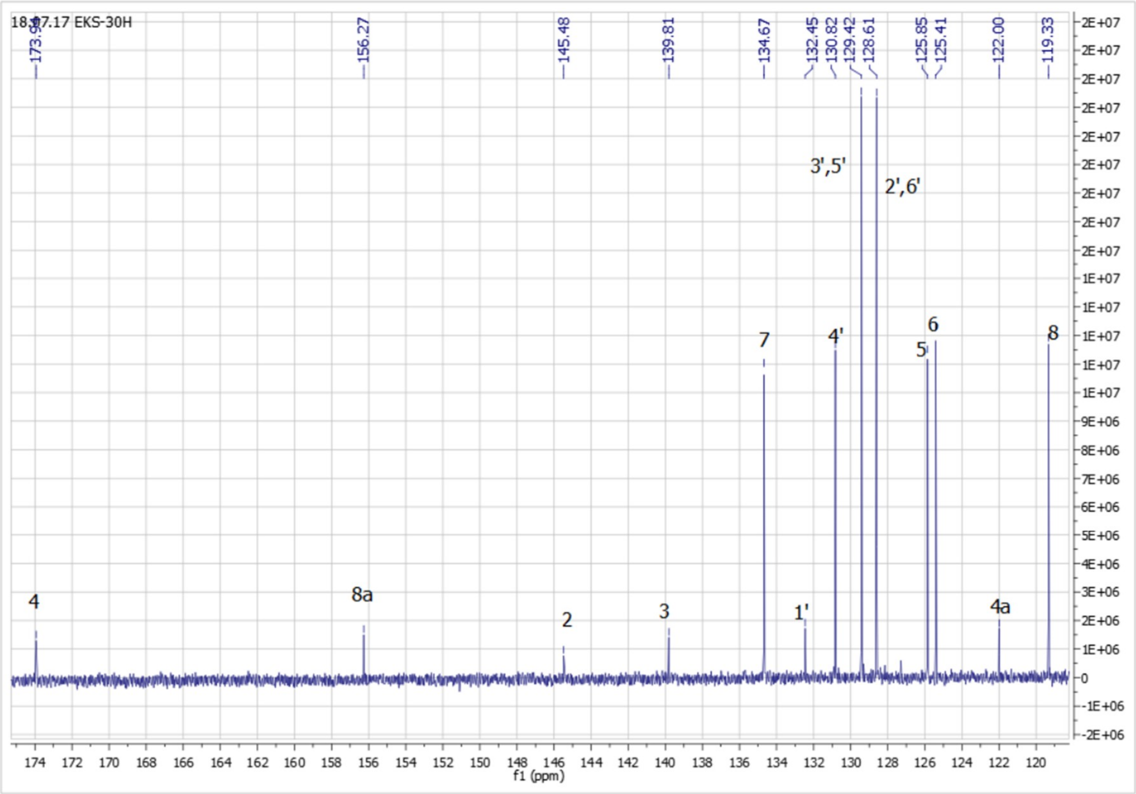

Figure S2. ^{13}C NMR spectrum of 3-hydroxyflavone (1) (Acetone-d_6, 151 MHz)

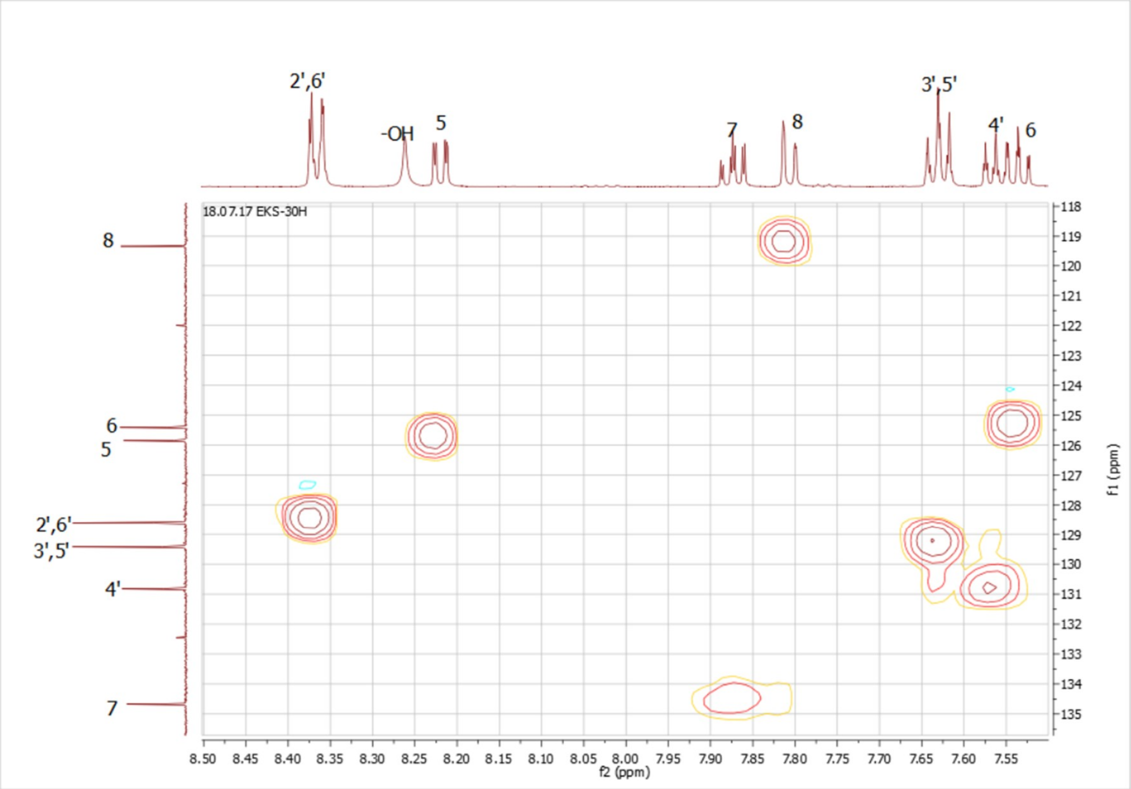

Figure S3. HSQC NMR spectrum of 3-hydroxyflavone (1) (Acetone-d_6, 151 MHz)

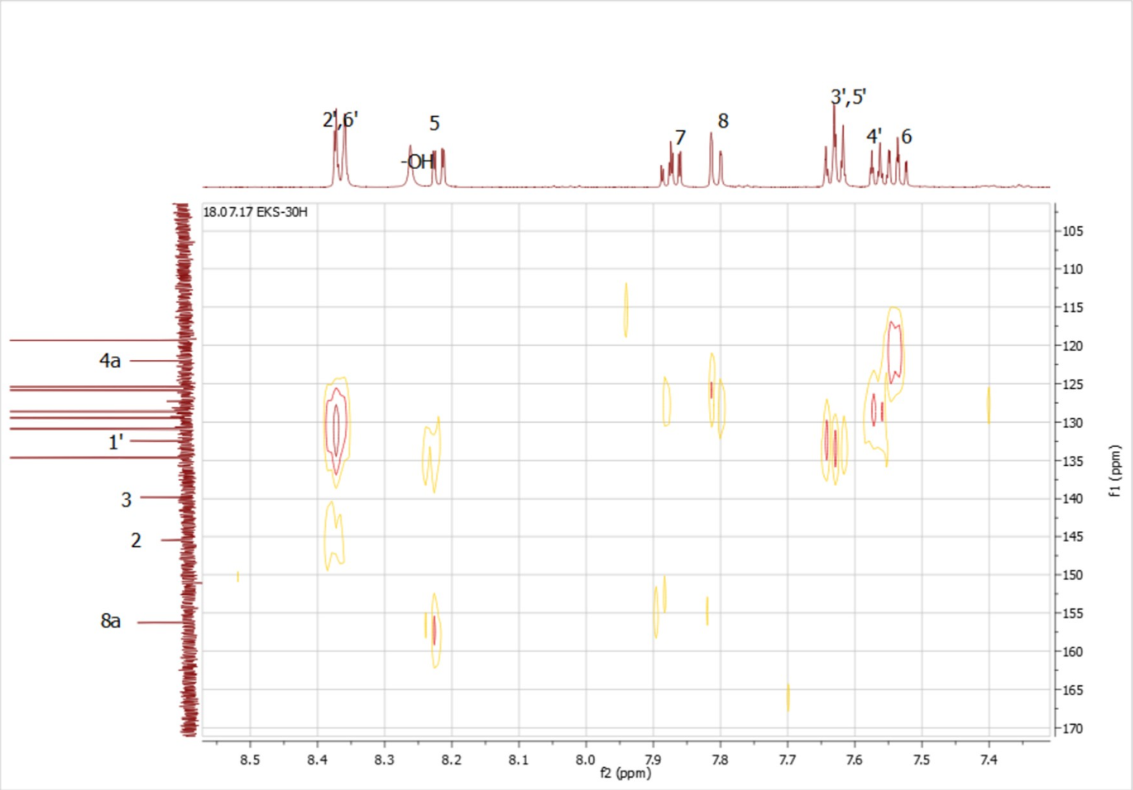

Figure S4. HMBC NMR spectrum of 3-hydroxyflavone (1) (Acetone-d_6, 151 MHz)

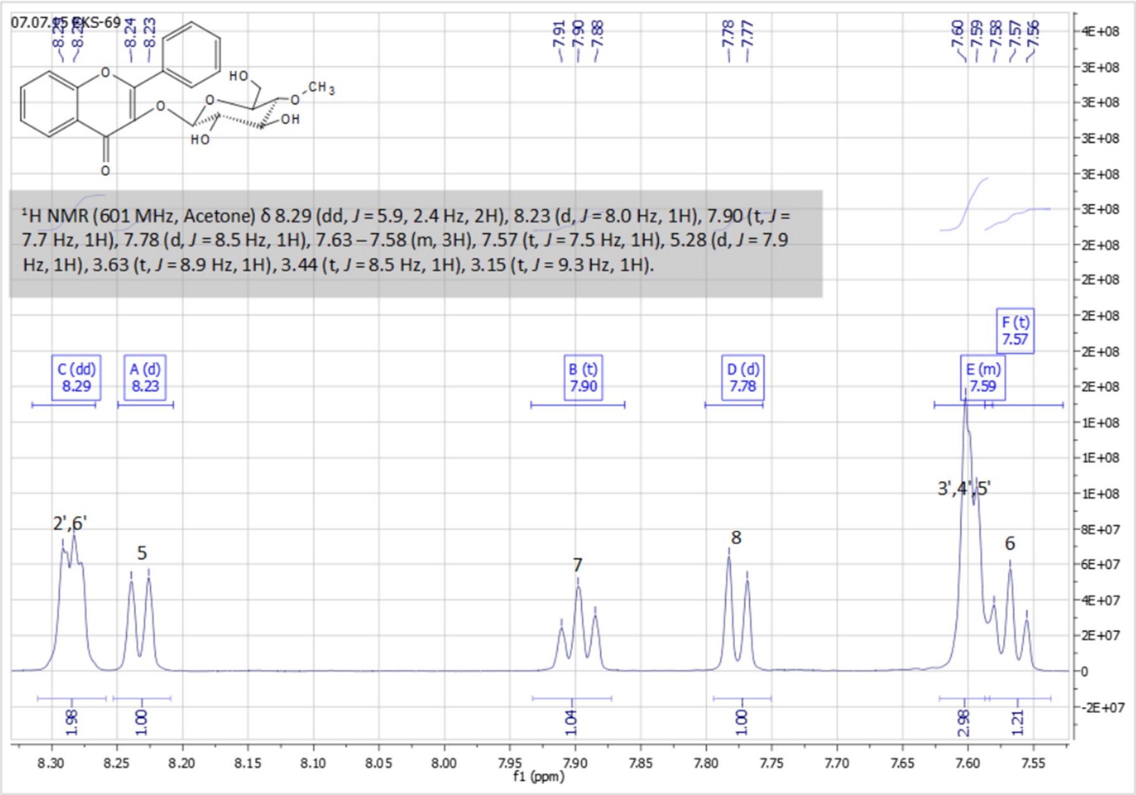

Figure S5. ¹H NMR spectrum of flavone 3-*O*-*β*-D-(4″-*O*-methyl)-glucopyranoside (1a) (Acetone-d6, 600 MHz)

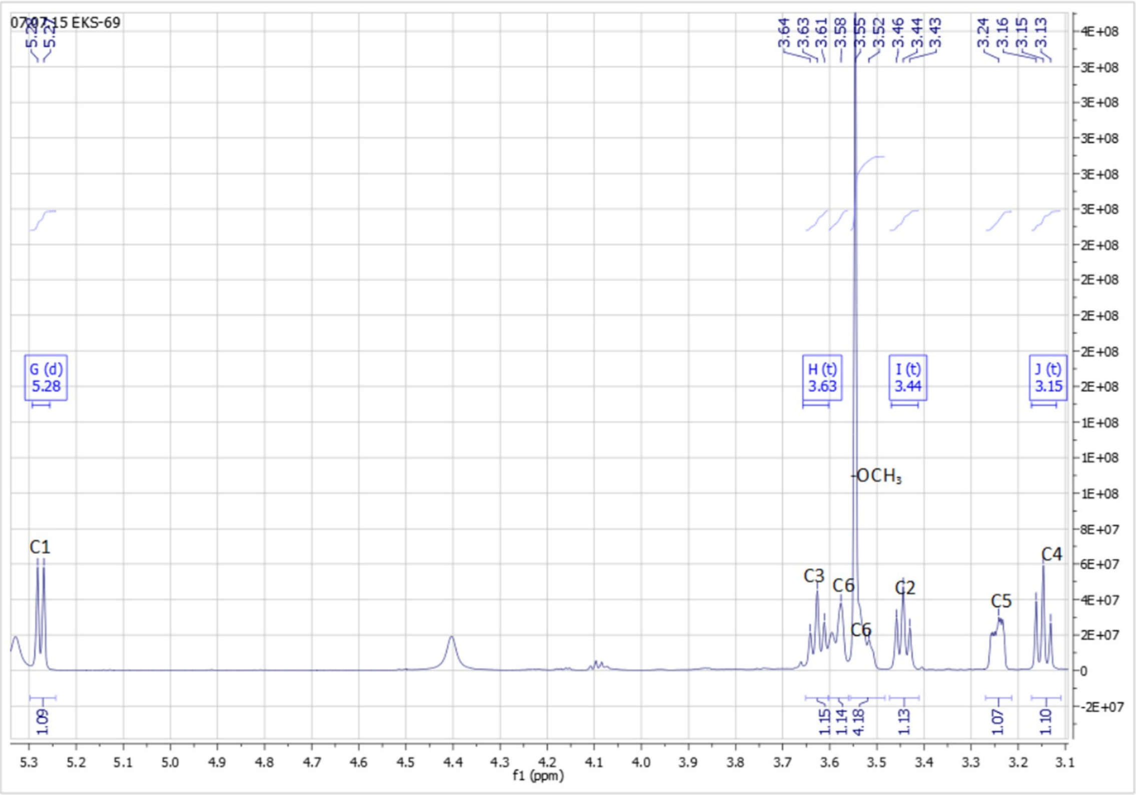

Figure S6. ¹H NMR spectrum of flavone 3-*O*-*β*-D-(4″-*O*-methyl)-glucopyranoside (1a) (Acetone-d6, 600 MHz)

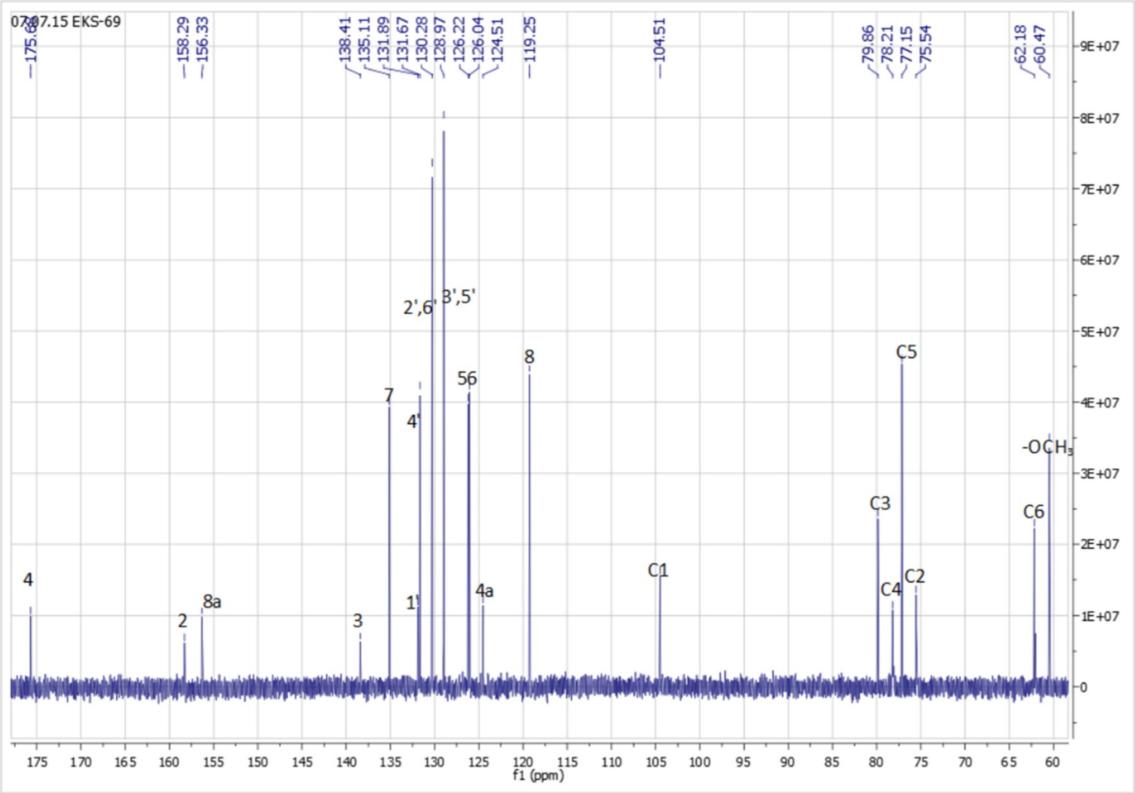

Figure S7. ^{13}C NMR spectrum of flavone 3-*O*-*β*-D-(4″-*O*-methyl)-glucopyranoside (1a) (Acetone-d6, 151 MHz)

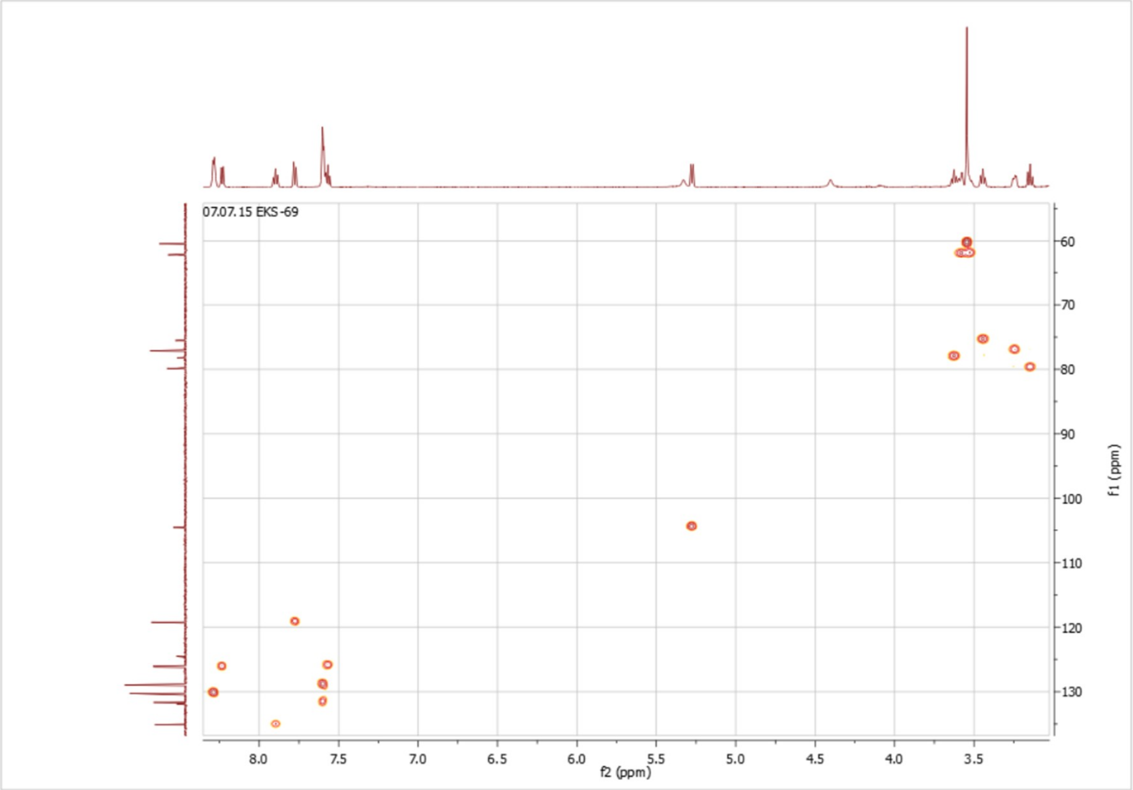

Figure S8. HSQC NMR spectrum of flavone 3-*O*-*β*-D-(4″-*O*-methyl)-glucopyranoside (1a) (Acetone-d6, 151 MHz)

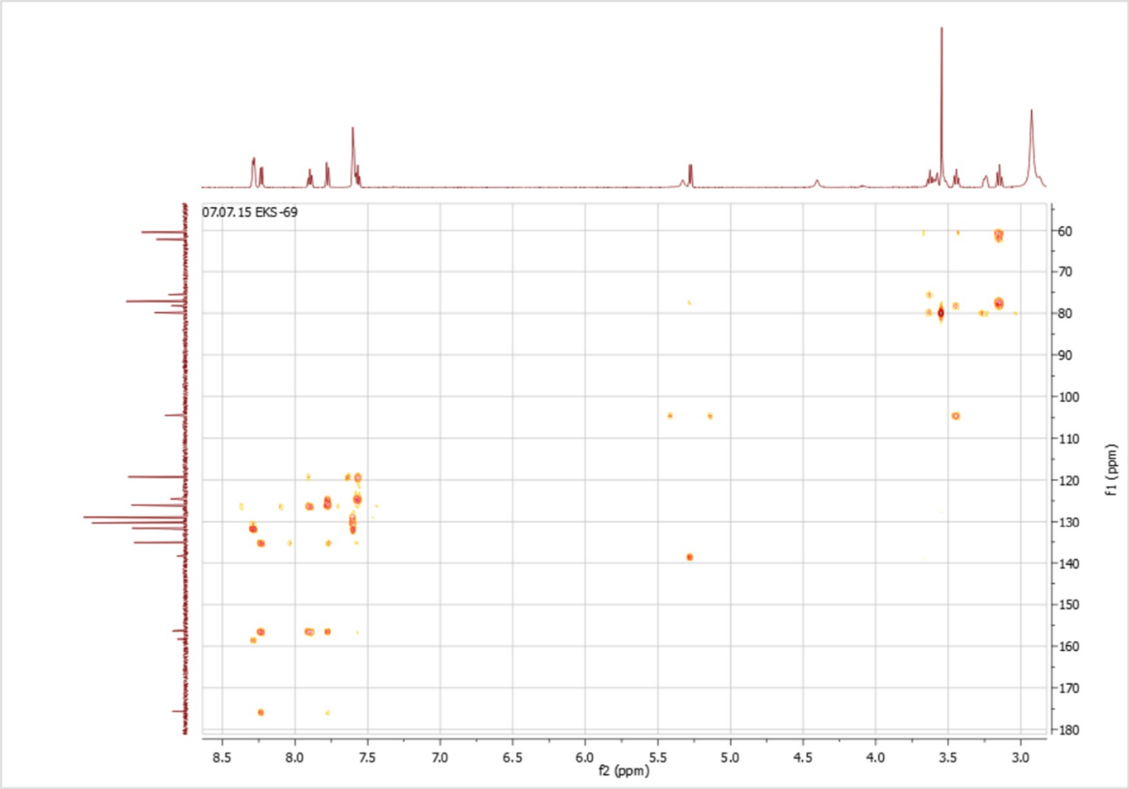

Figure S9. HMBC NMR spectrum of flavone 3-*O*-*β*-D-(4′′-*O*-methyl)-glucopyranoside (1a) (Acetone-d$_6$, 151 MHz)

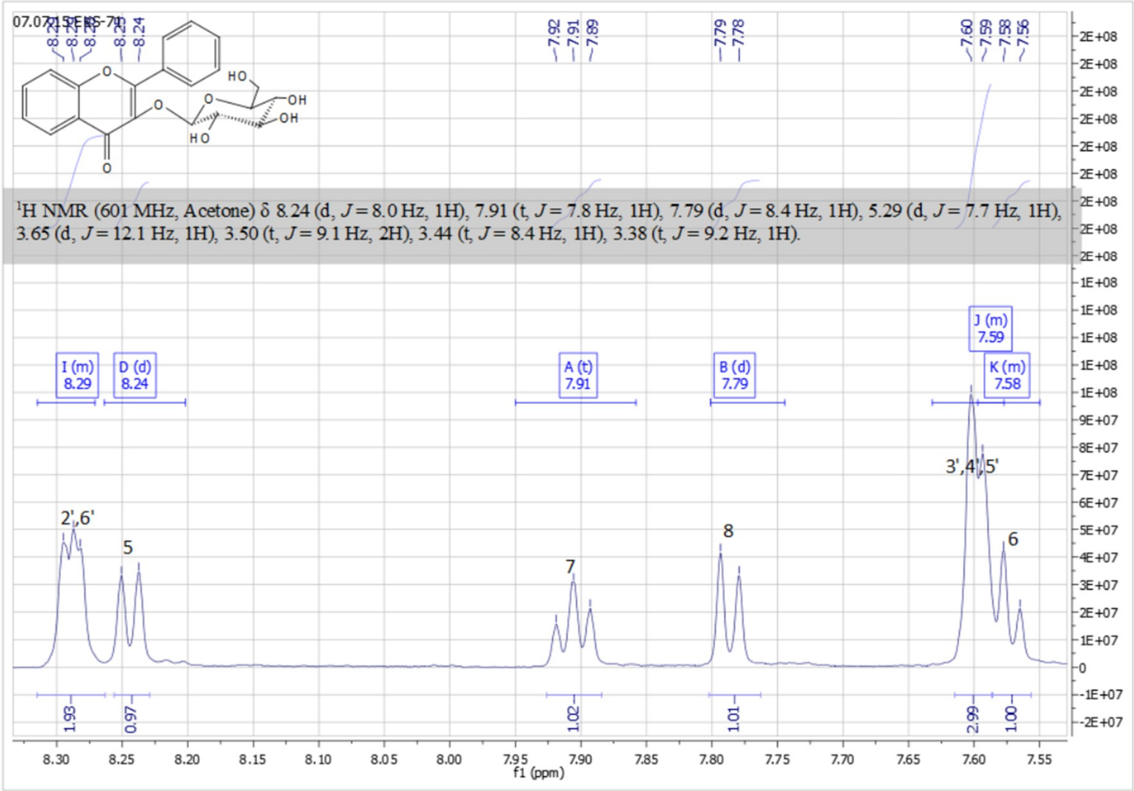

Figure S10. ^{1}H NMR spectrum of flavone 3-*O*-*β*-D-glucopyranoside (1b) (Acetone-d$_6$, 600 MHz)

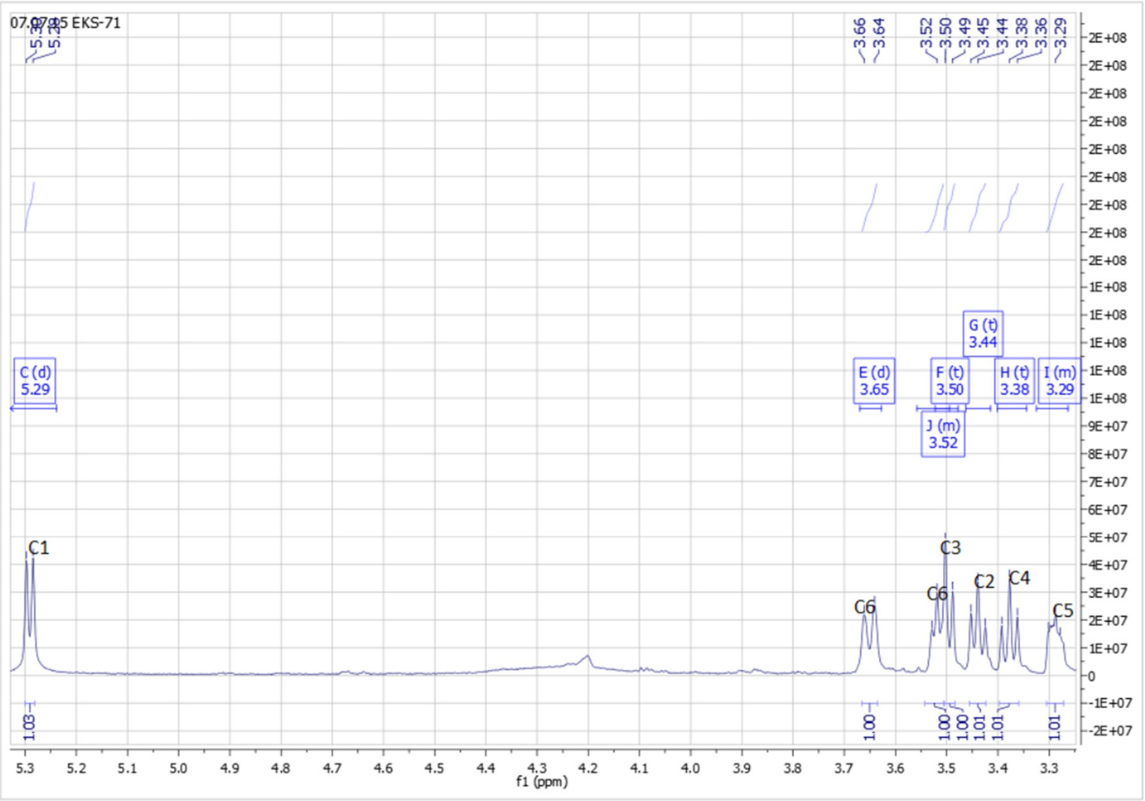

Figure S11. ^{1}H NMR spectrum of flavone 3-*O*-*β*-D-glucopyranoside (1b) (Acetone-d_6, 600 MHz)

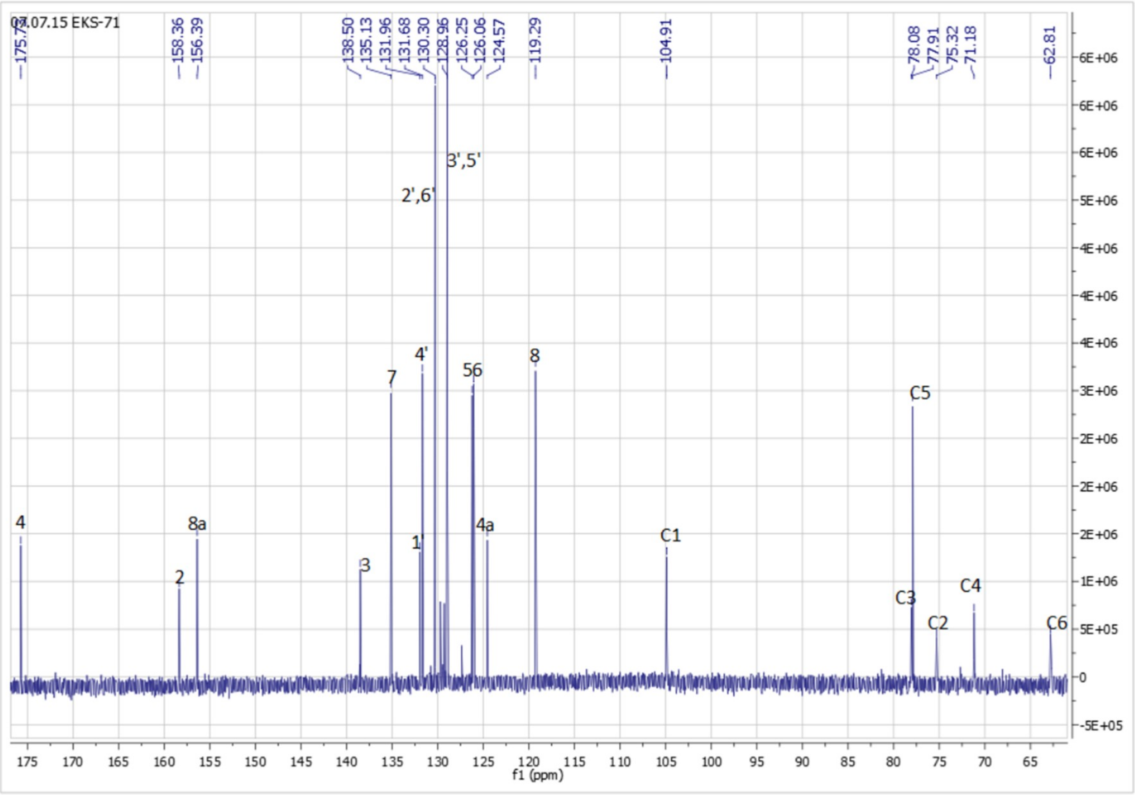

Figure S12. ^{13}C NMR spectrum of flavone 3-*O*-*β*-D-glucopyranoside (1b) (Acetone-d_6, 151 MHz)

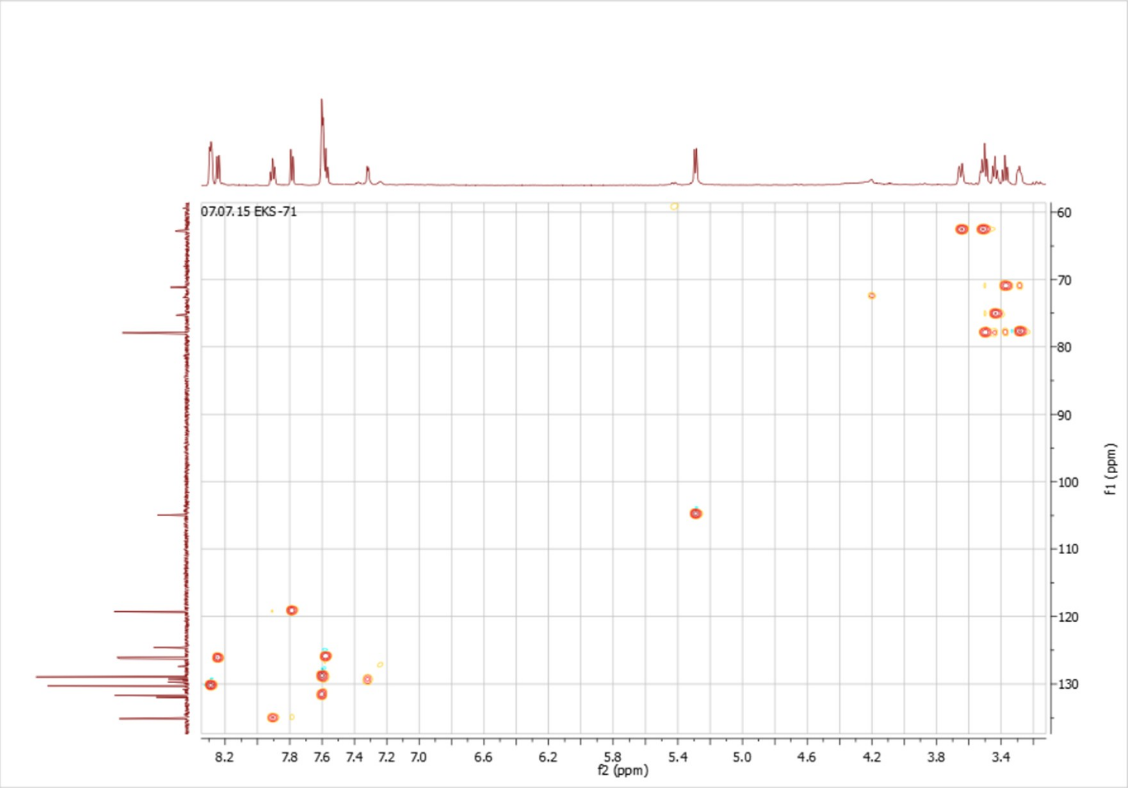

Figure S13. HSQC NMR spectrum of flavone 3-*O*-*β*-D-glucopyranoside (1b) (Acetone-d$_6$, 151 MHz)

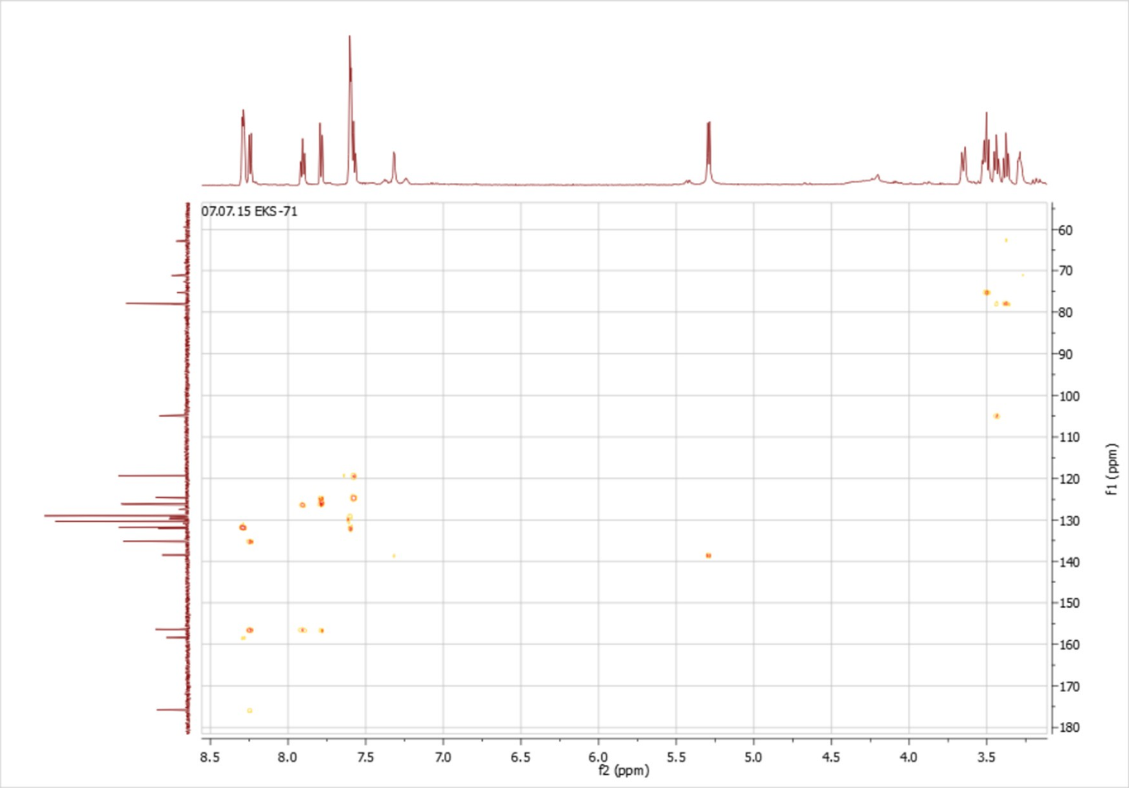

Figure S14. HMBC NMR spectrum of flavone 3-*O*-*β*-D-glucopyranoside (1b) (Acetone-d$_6$, 151 MHz)

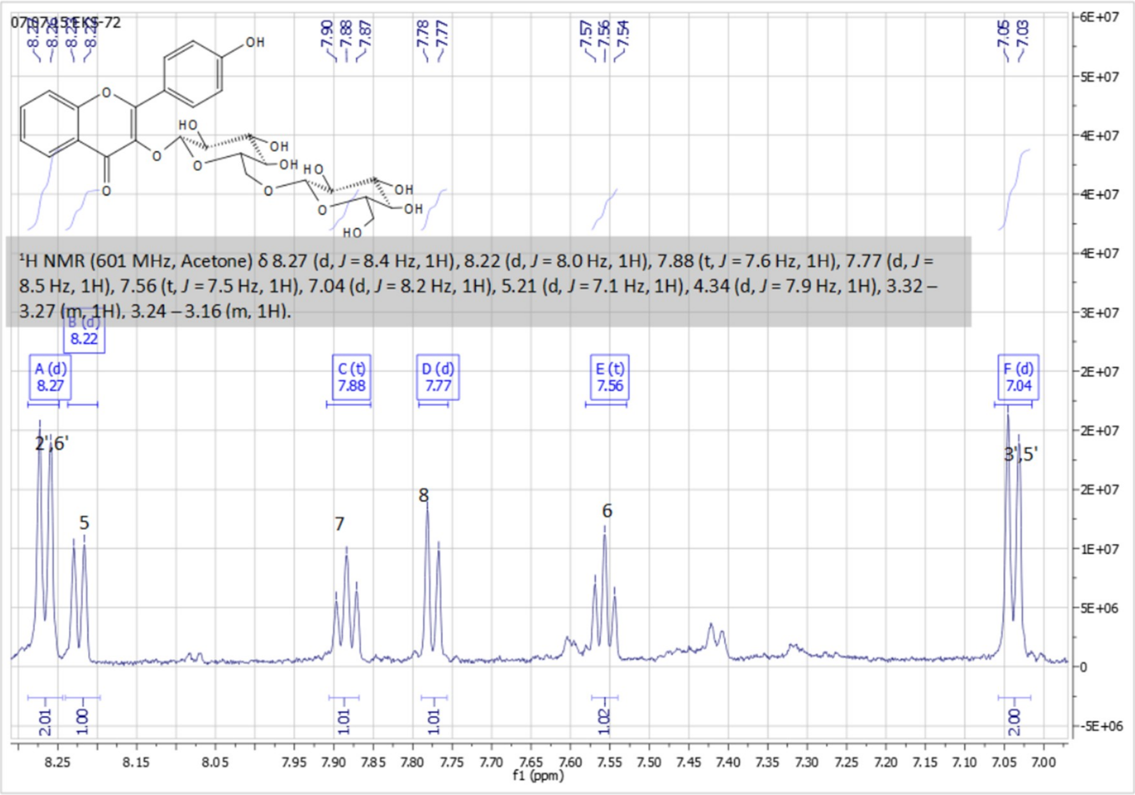

Figure S15. ¹H NMR spectrum of 3-*O*-[*β*-D-glucopyranosyl-(1→6)-*β*-D-glucopyranosyl]-4′-hydroxyflavone (1c) (Acetone-d₆, 600 MHz)

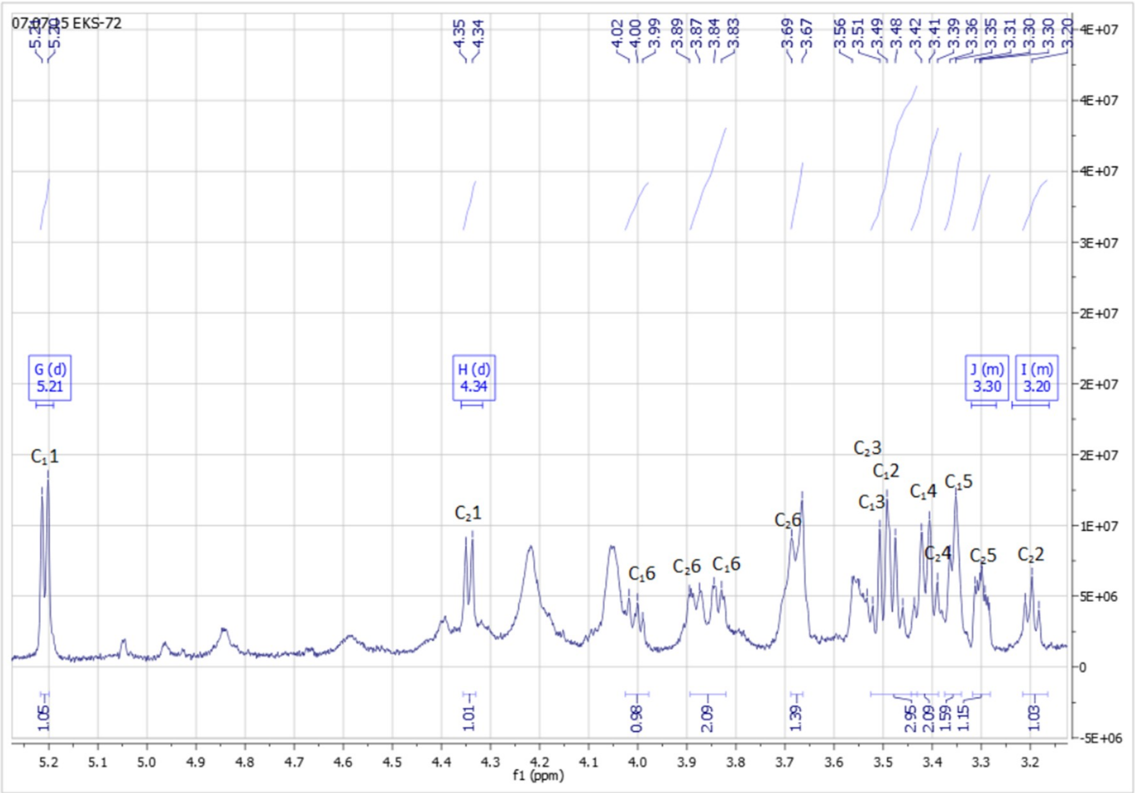

Figure S16. ¹H NMR spectrum of 3-*O*-[*β*-D-glucopyranosyl-(1→6)-*β*-D-glucopyranosyl]-4′-hydroxyflavone (Acetone-d₆, 600 MHz)

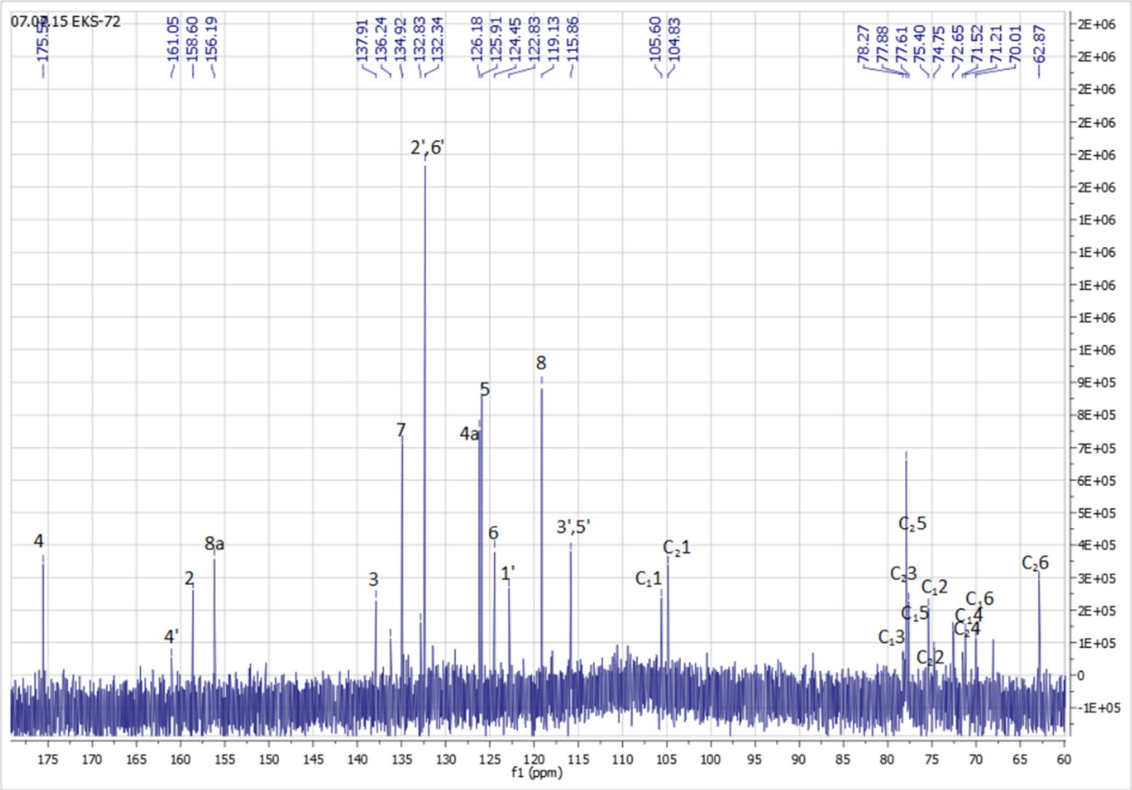

Figure S17. ^{13}C NMR spectrum of 3-*O*-[*β*-D-glucopyranosyl-(1→6)-*β*-D-glucopyranosyl]-4′-hydroxyflavone (Acetone-d$_6$, 151 MHz)

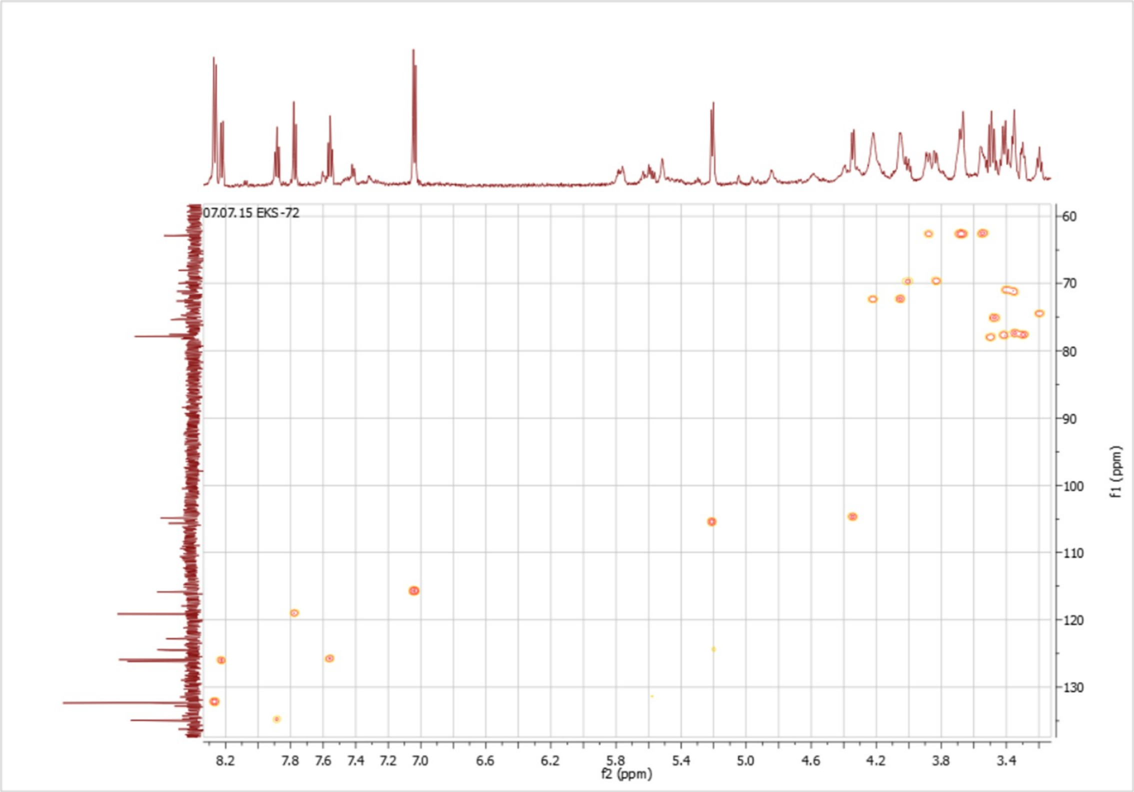

Figure S18. HSQC NMR spectrum of 3-*O*-[*β*-D-glucopyranosyl-(1→6)-*β*-D-glucopyranosyl]-4′-hydroxyflavone (Acetone-d$_6$, 151 MHz)

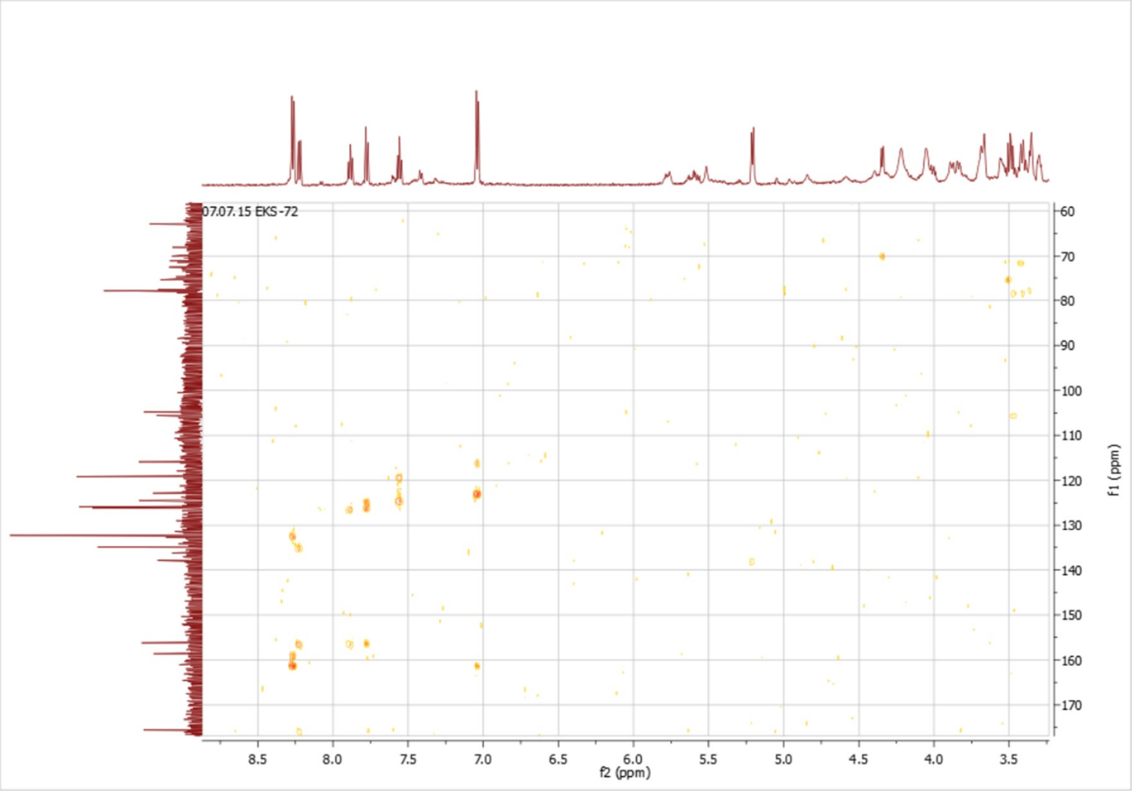

Figure S19. HMBC NMR spectrum of 3-*O*-[*β*-D-glucopyranosyl-(1→6)-*β*-D-glucopyranosyl]-4′-hydroxyflavone (Acetone-d6, 151 MHz)

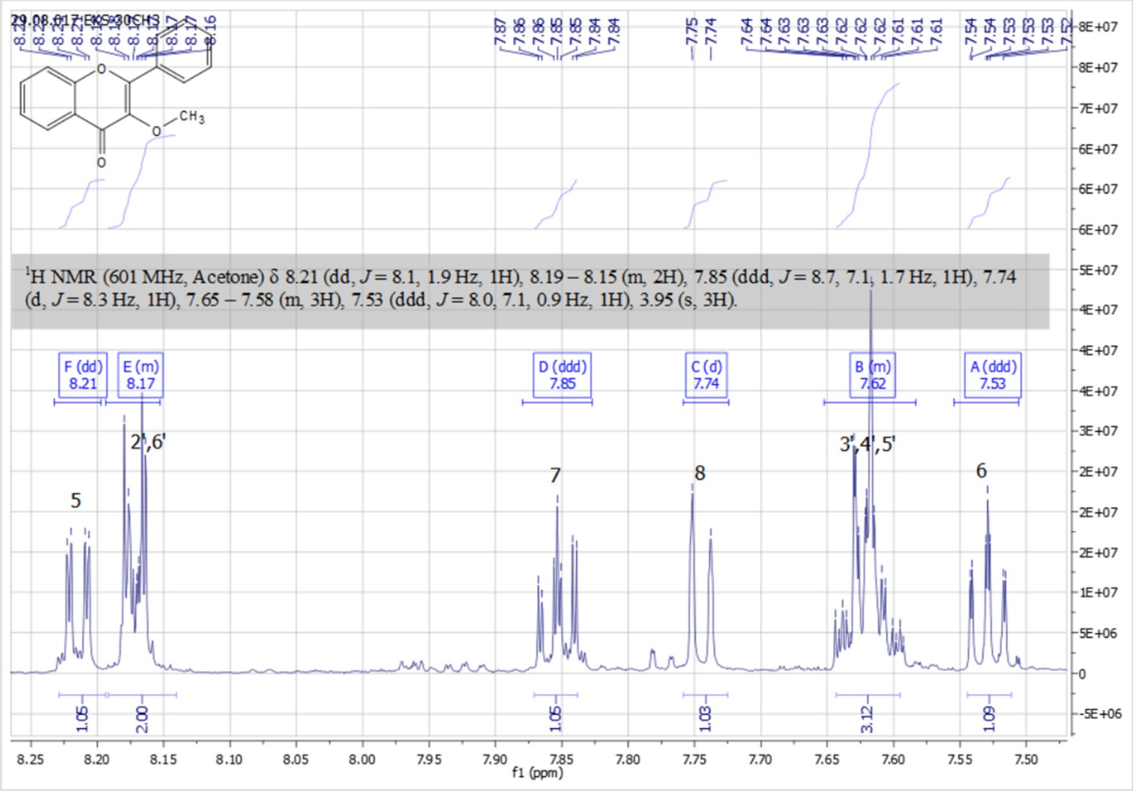

Figure S20. ^{1}H NMR spectrum of 3-methoxyflavone (2) (Acetone-d6, 600 MHz)

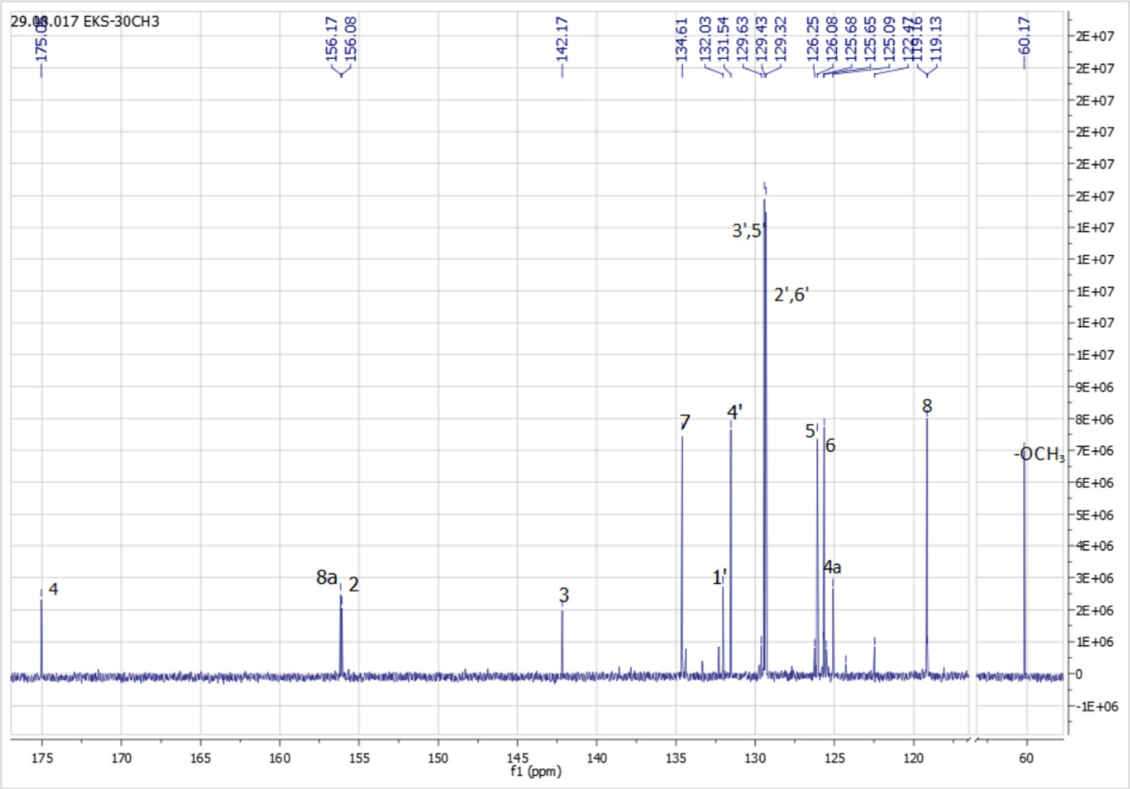

Figure S21. ^{13}C NMR spectrum of 3-methoxyflavone (2) (Acetone-d$_6$, 151 MHz)

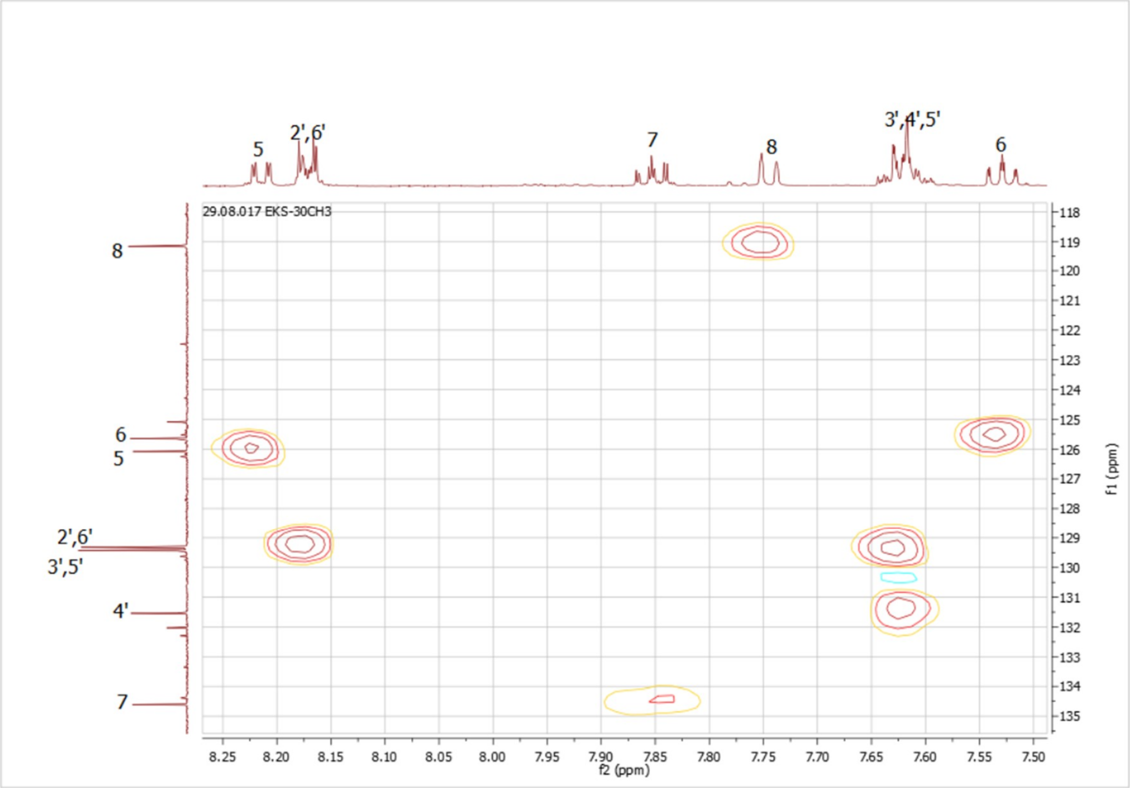

Figure S22. HSQC NMR spectrum of 3-methoxyflavone (2) (Acetone-d$_6$, 151 MHz)

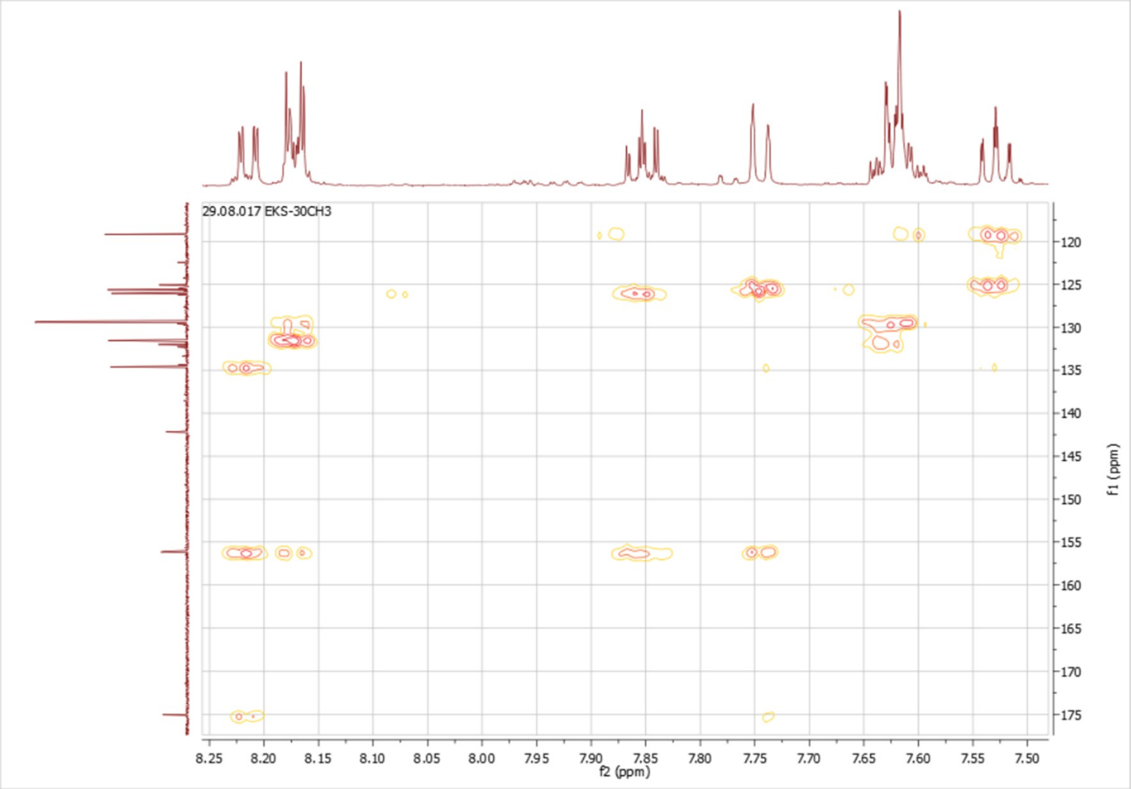

Figure S23. HMBC NMR spectrum of 3-methoxyflavone (2) (Acetone-d_6, 151 MHz)

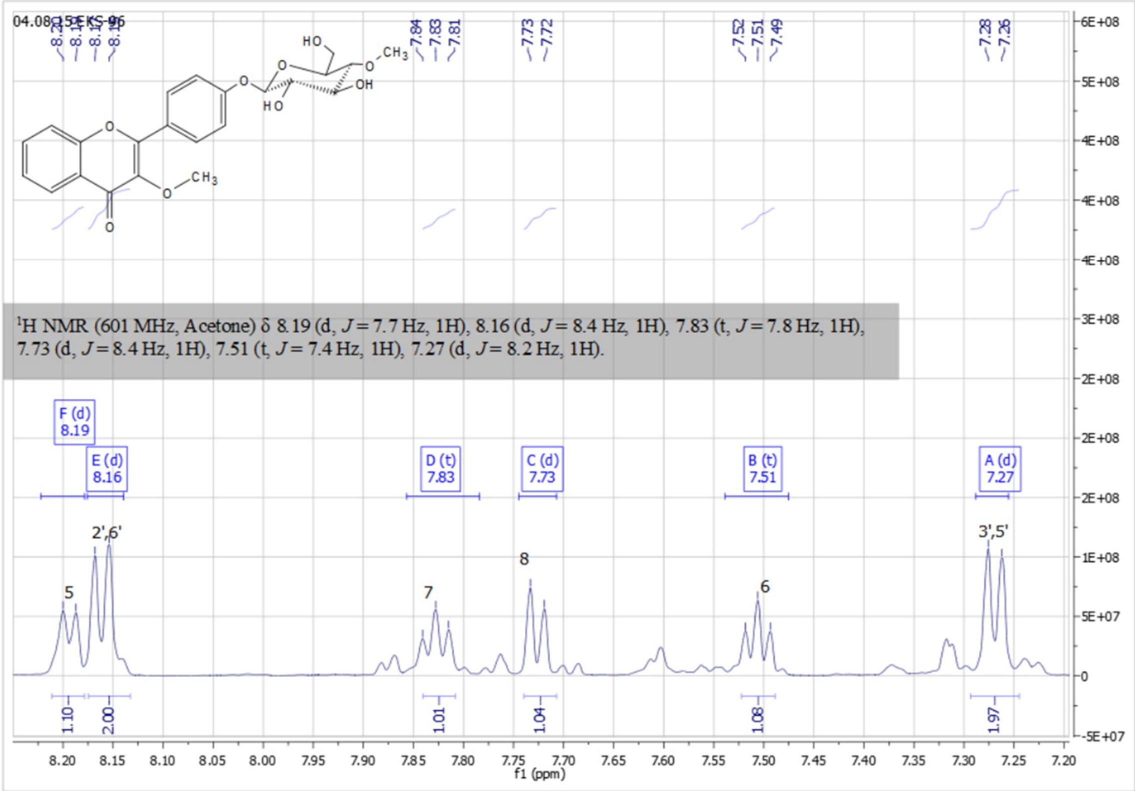

Figure S24. ^{1}H NMR spectrum of 3-methoxyflavone 4′-*O*-*β*-D-(4″-*O*-methyl)-glucopyranoside (2a) (Acetone-d_6, 600 MHz)

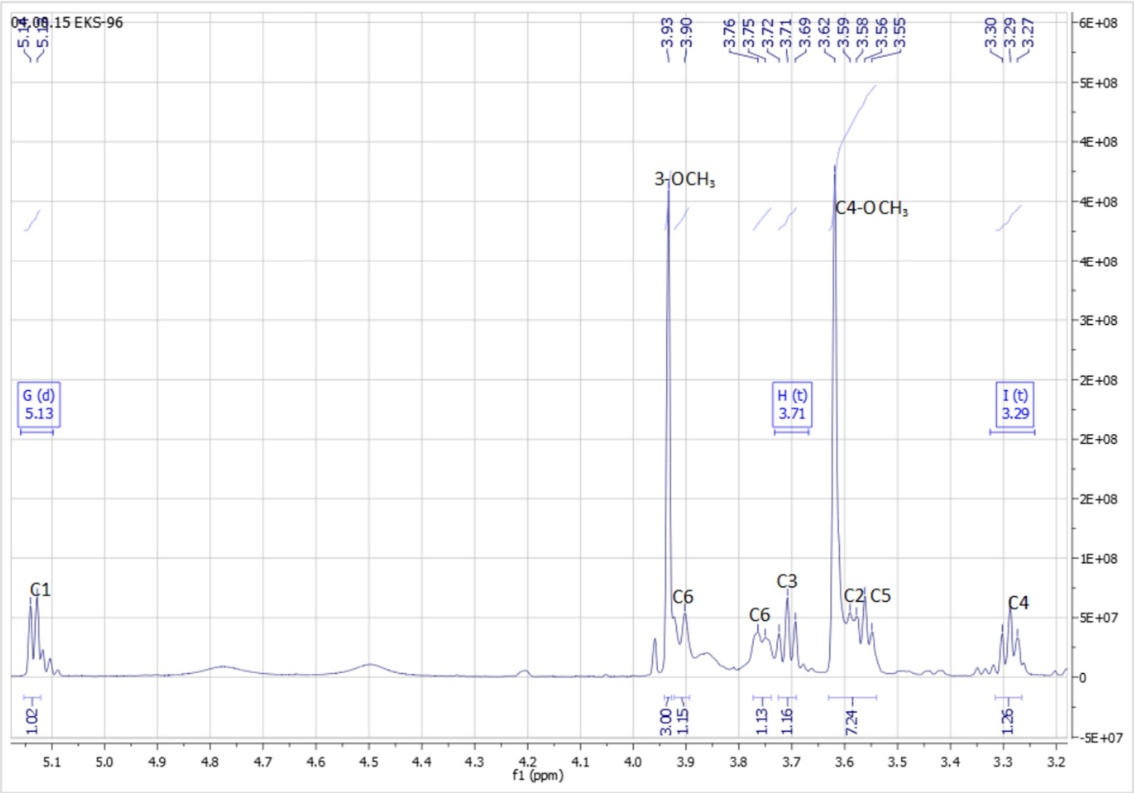

Figure S25. ^{1}H NMR spectrum of 3-methoxyflavone 4′-*O*-*β*-D-(4″-*O*-methyl)-glucopyranoside (2a) (Acetone-d6, 600 MHz)

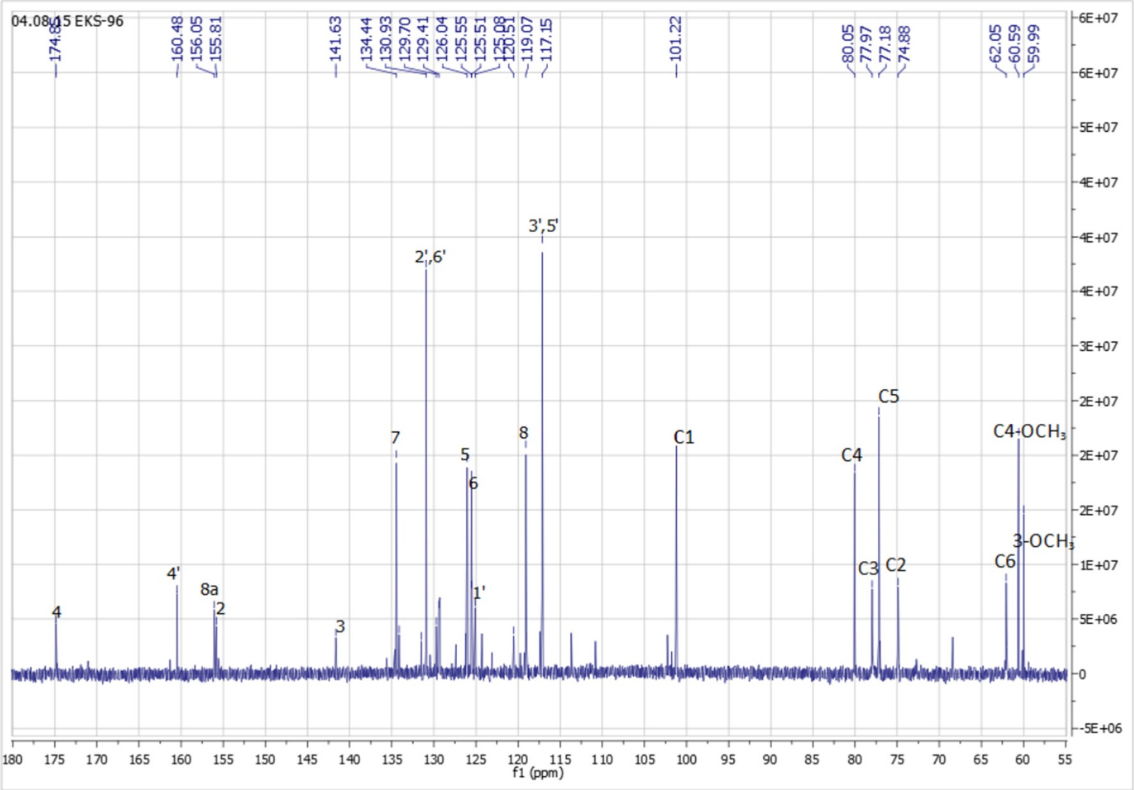

Figure S26. ^{13}C NMR spectrum of 3-methoxyflavone 4′-*O*-*β*-D-(4″-*O*-methyl)-glucopyranoside (2a) (Acetone-d6, 151 MHz)

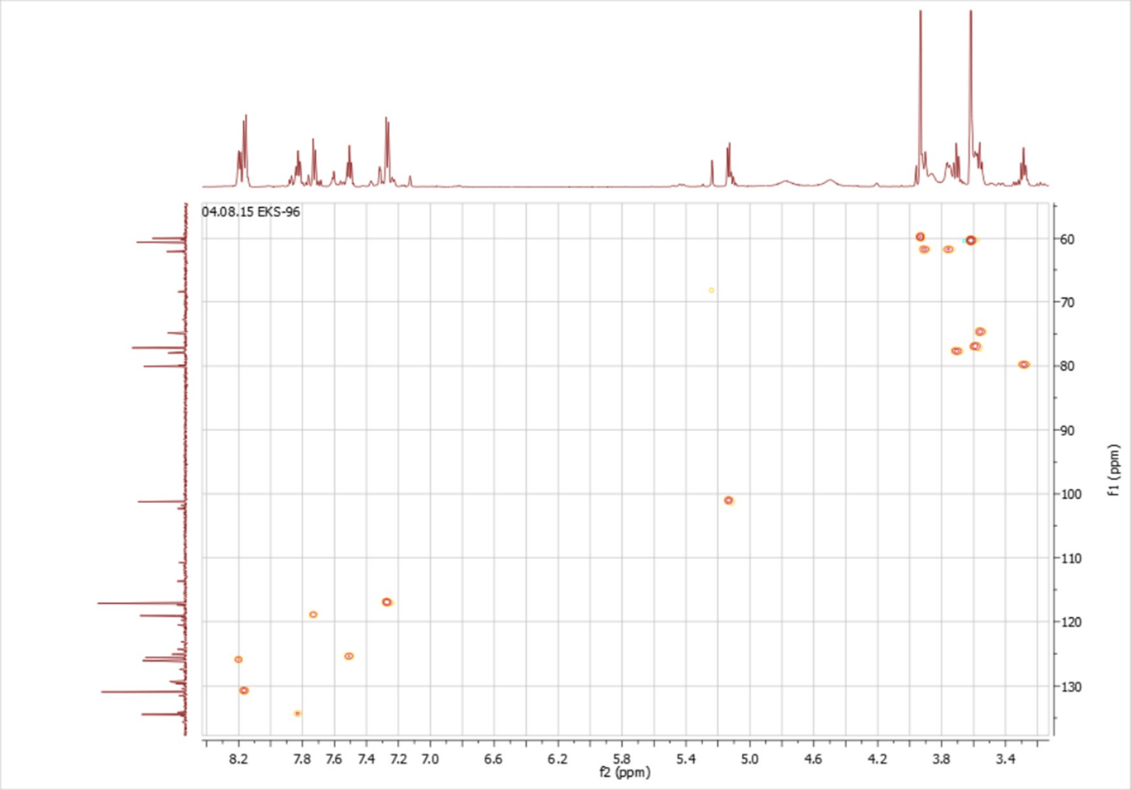

Figure S27. HSQC NMR spectrum of 3-methoxyflavone 4′-*O*-*β*-D-(4″-*O*-methyl)-glucopyranoside (2a) (Acetone-d_6, 151 MHz)

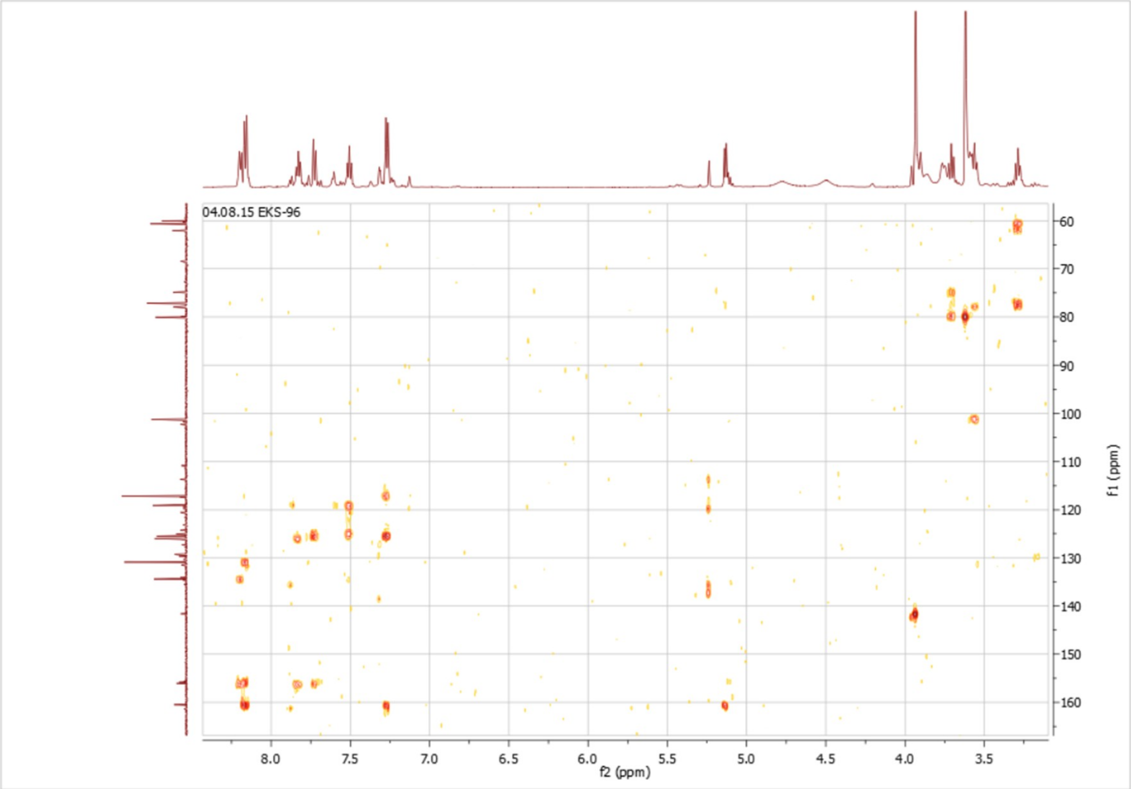

Figure S28. HMBC NMR spectrum of 3-methoxyflavone 4′-*O*-*β*-D-(4″-*O*-methyl)-glucopyranoside (2a) (Acetone-d_6, 151 MHz)

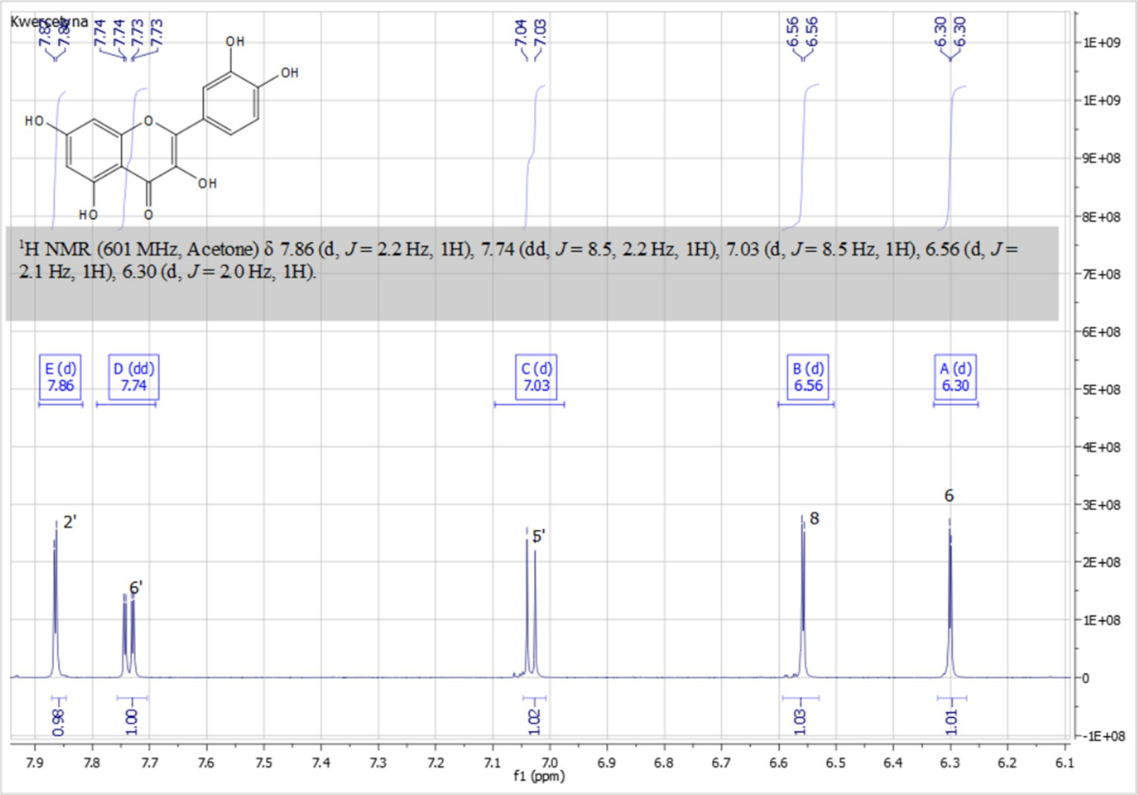

Figure S29. ^{1}H NMR spectrum of 3,3′,4′,5,7-Pentahydroxyflavone (Quercetin) (3) (Acetone-d$_6$, 600 MHz)

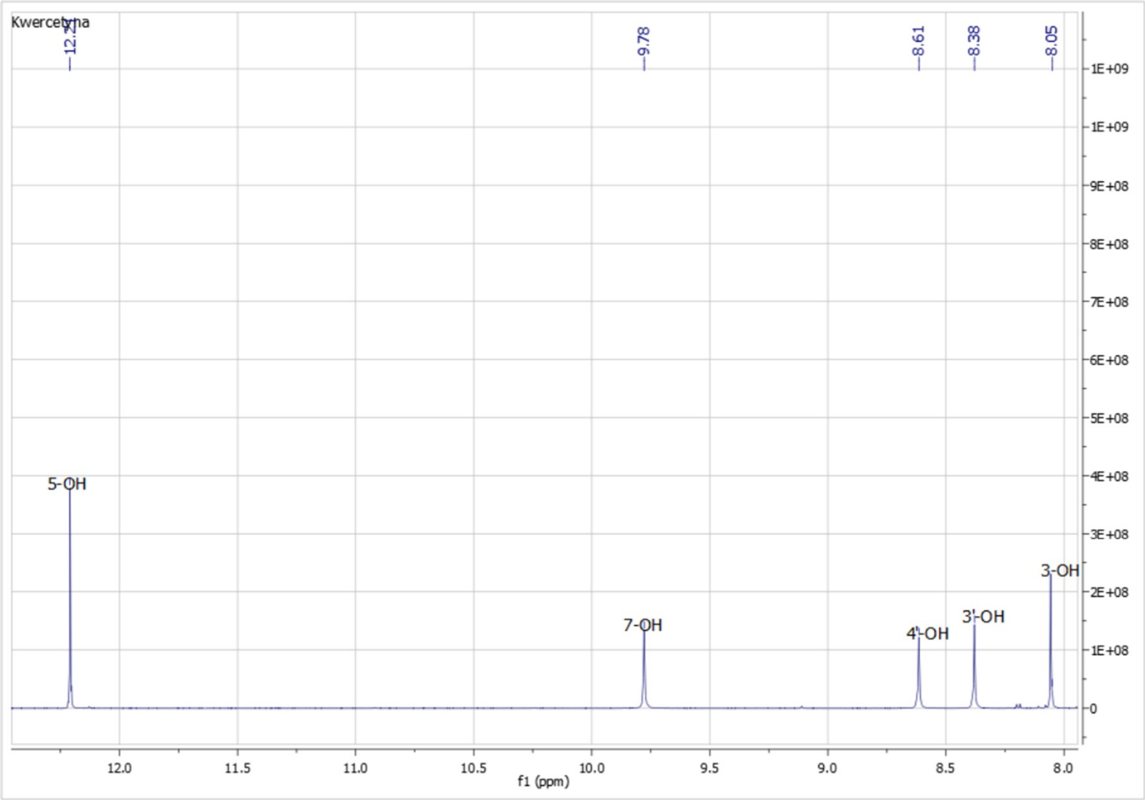

Figure S30. ^{1}H NMR spectrum of 3,3′,4′,5,7-Pentahydroxyflavone (Quercetin) (3) (Acetone-d$_6$, 600 MHz)

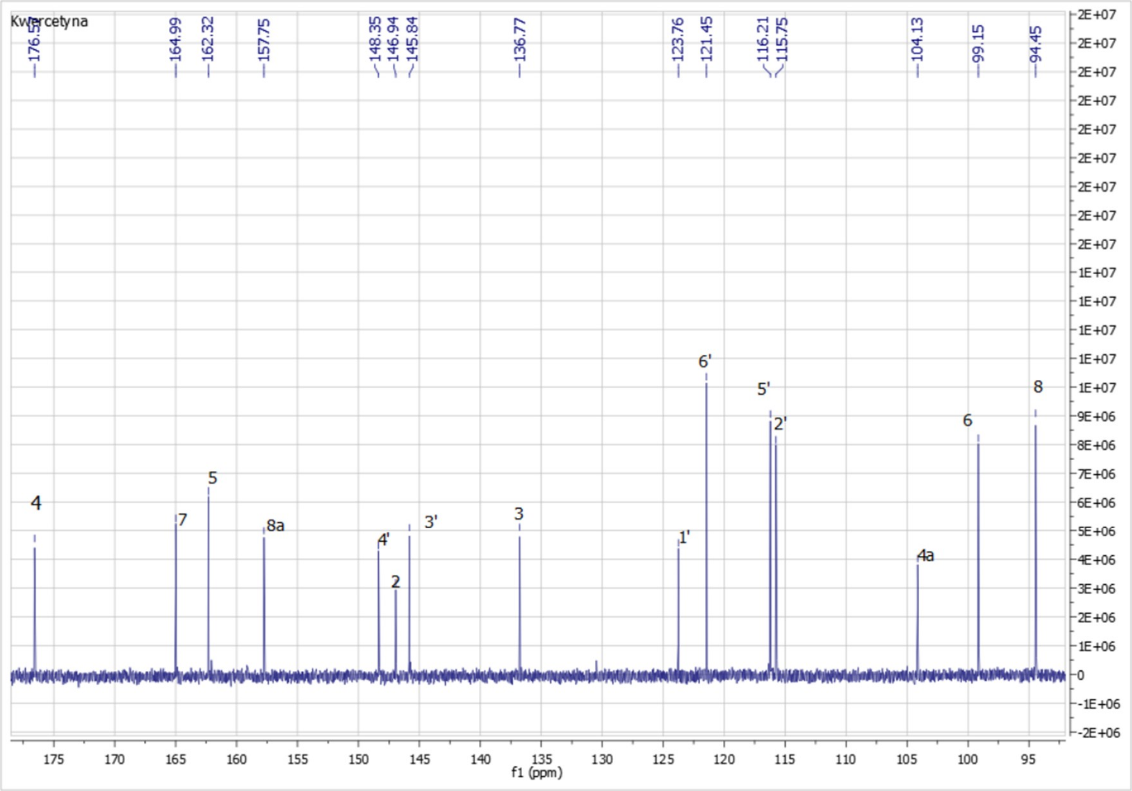

Figure S31. ^{13}C NMR spectrum of 3,3′,4′,5,7-Pentahydroxyflavone (Quercetin) (3) (Acetone-d_6, 151 MHz)

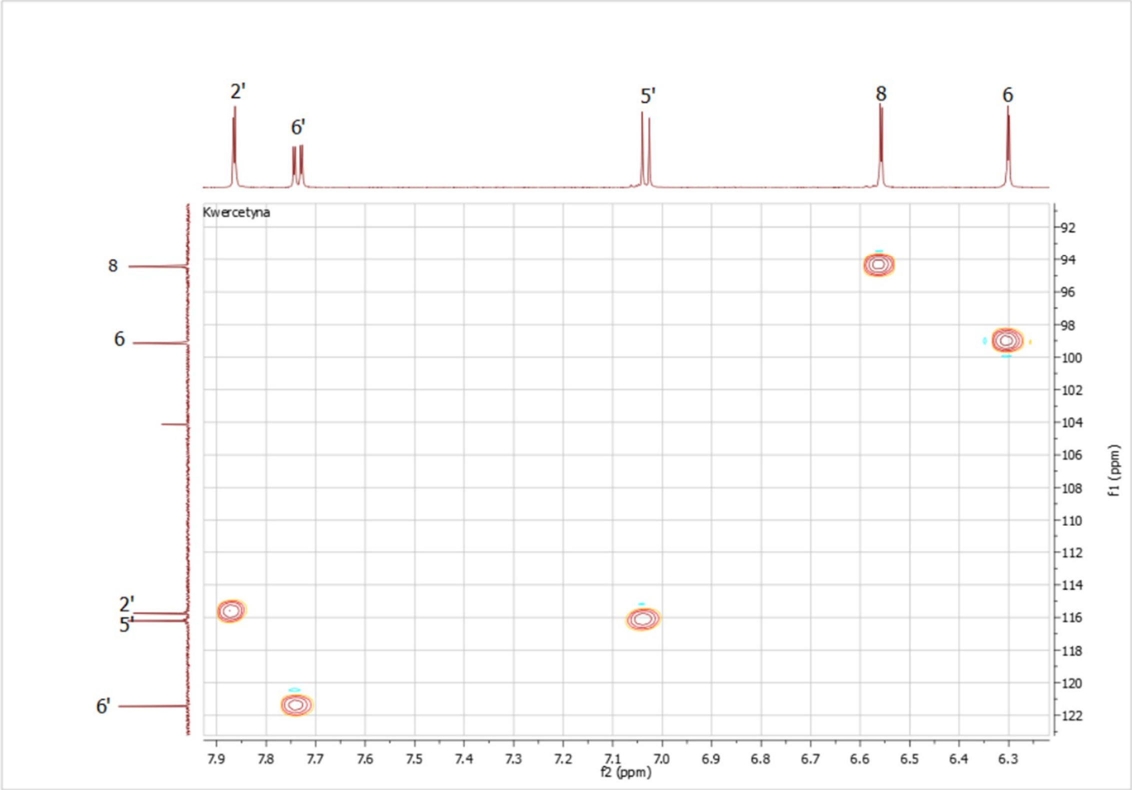

Figure S32. HSQC NMR spectrum of 3,3′,4′,5,7-Pentahydroxyflavone (Quercetin) (3) (Acetone-d_6, 151 MHz)

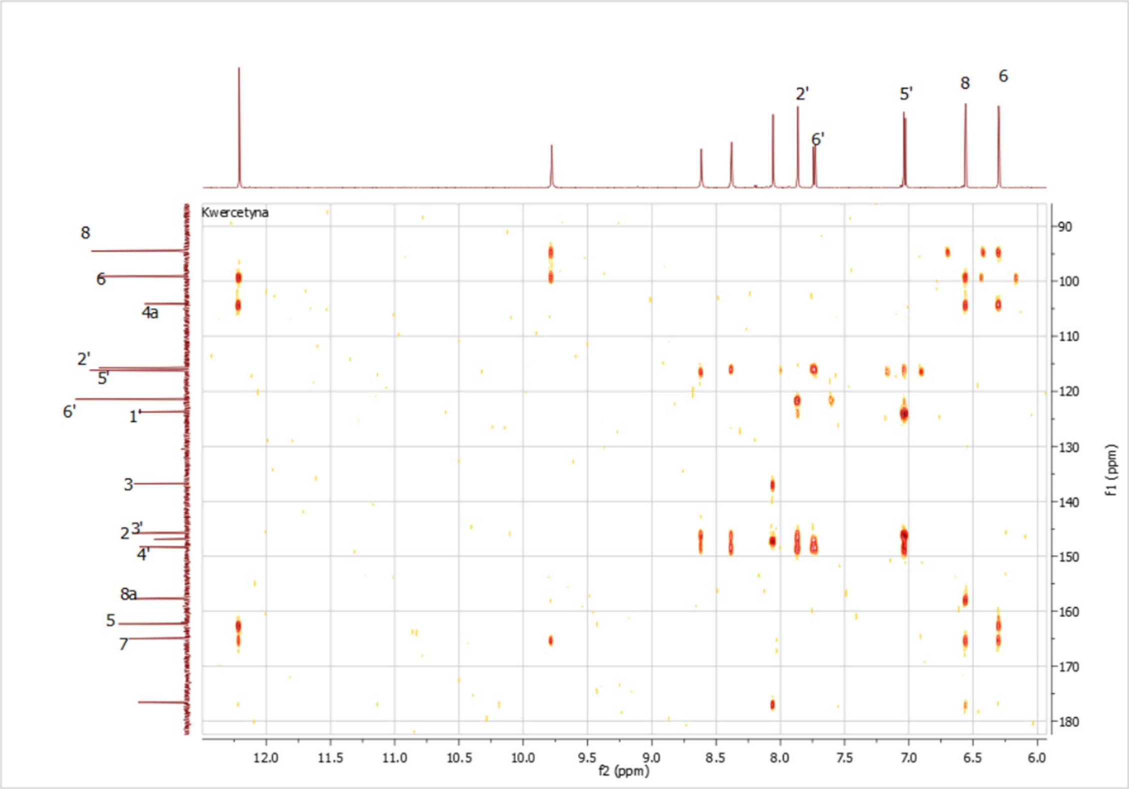

Figure S33. HMBC NMR spectrum of 3,3′,4′,5,7-Pentahydroxyflavone (Quercetin) (3) (Acetone-d₆, 151 MHz)

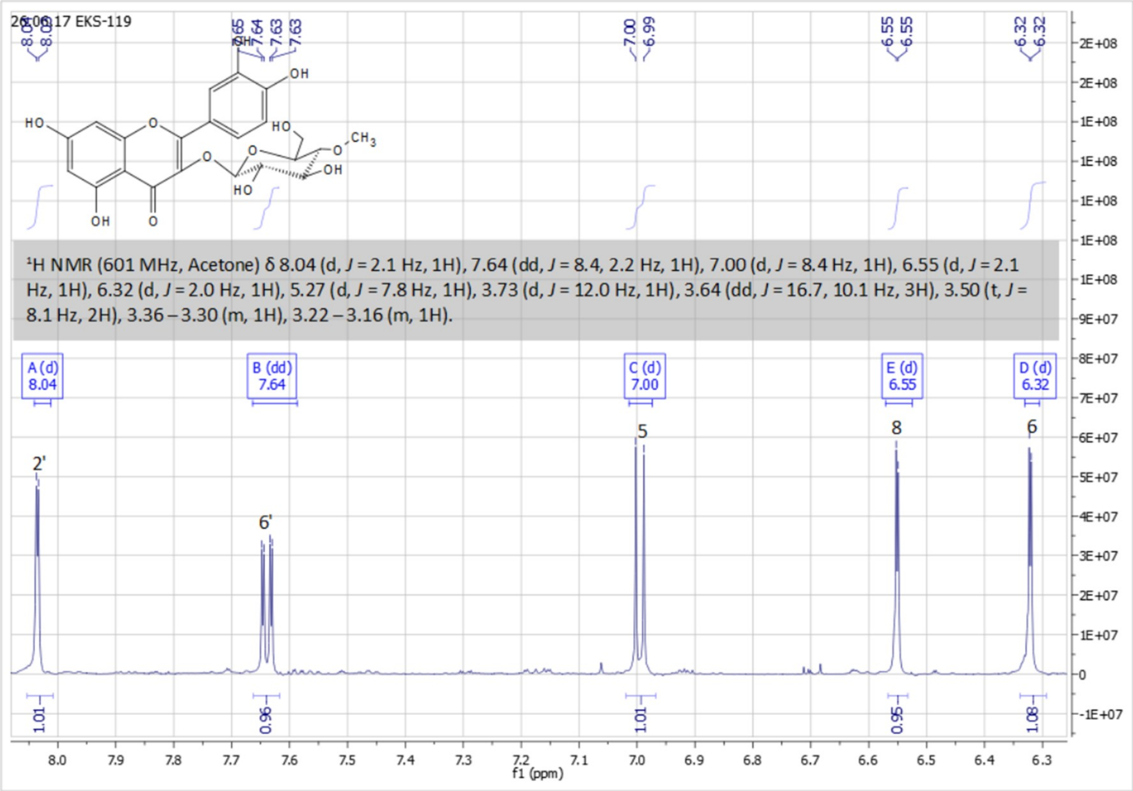

Figure S34. ¹H NMR spectrum of 3′,4′,5,7-tetrahydroxyflavone 3-*O*-*β*-D-(4′′-*O*-methyl)-glucopyranoside (3a) (Acetone-d₆, 600 MHz)

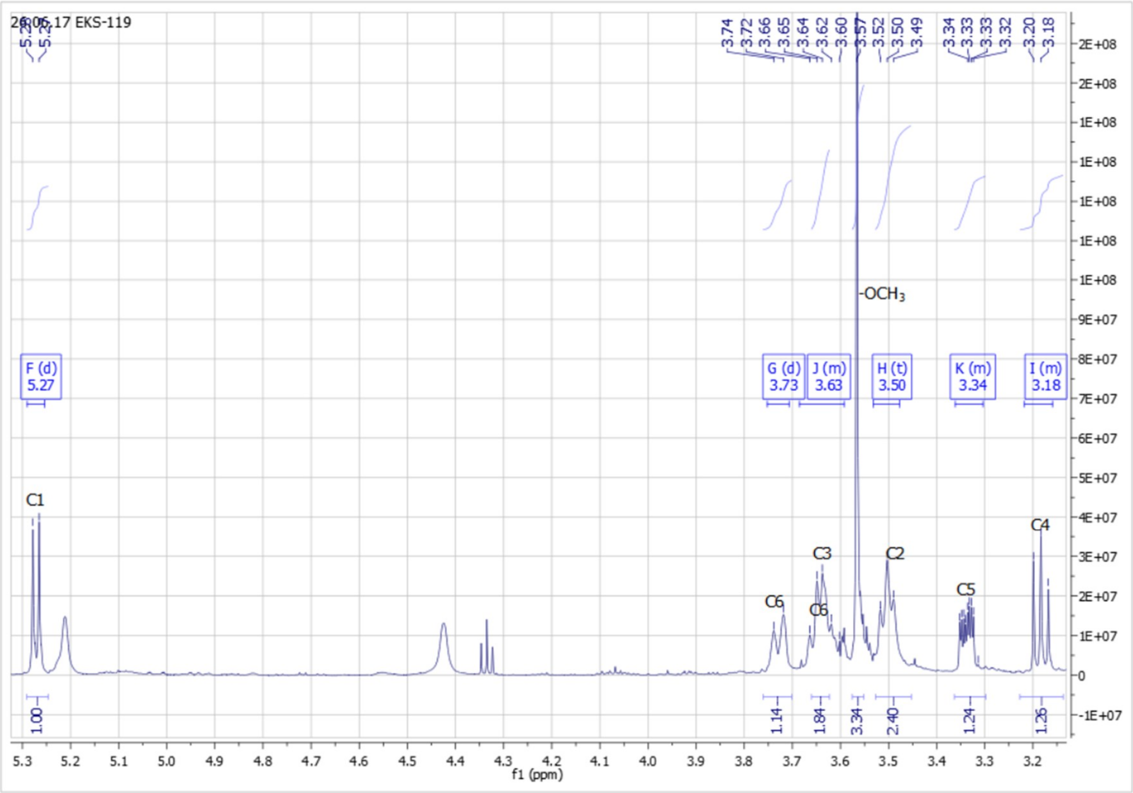

Figure S35. ^{1}H NMR spectrum of 3′,4′,5,7-tetrahydroxyflavone 3-*O*-*β*-D-(4′′-*O*-methyl)-glucopyranoside (3a) (Acetone-d_6, 600 MHz)

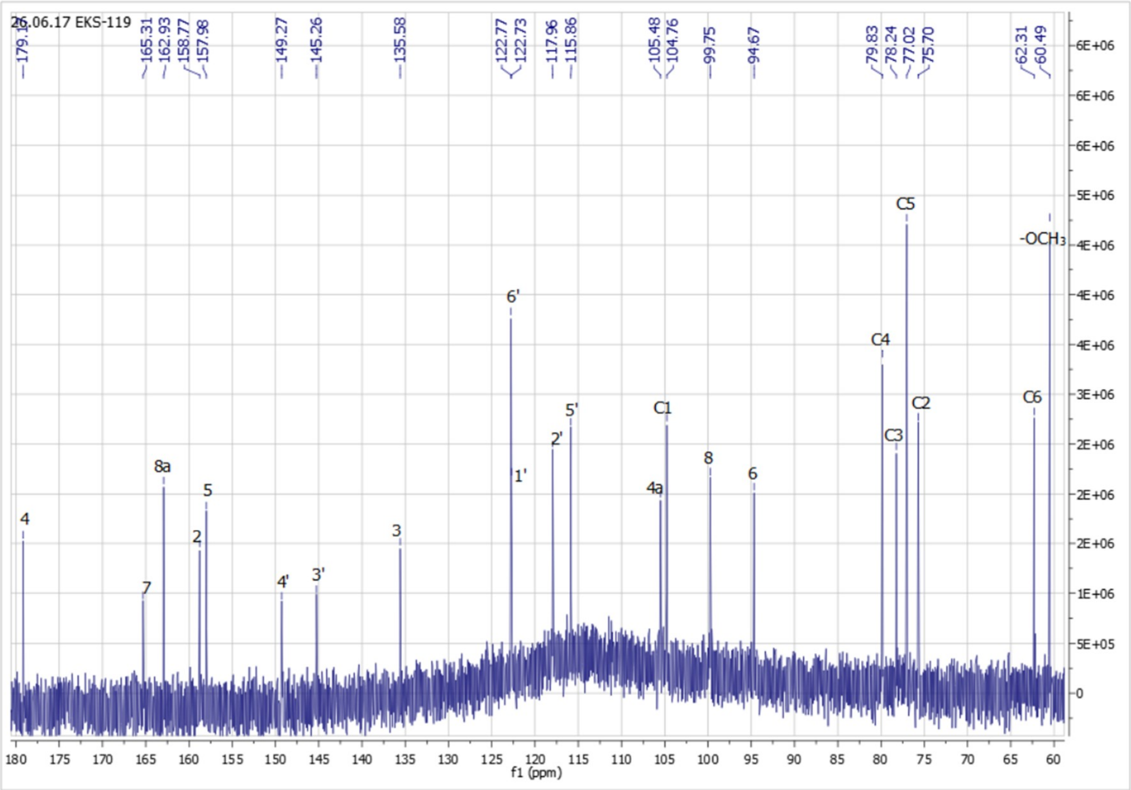

Figure S36. ^{13}C NMR spectrum of 3′,4′,5,7-tetrahydroxyflavone 3-*O*-*β*-D-(4′′-*O*-methyl)-glucopyranoside (3a) (Acetone-d_6, 151 MHz)

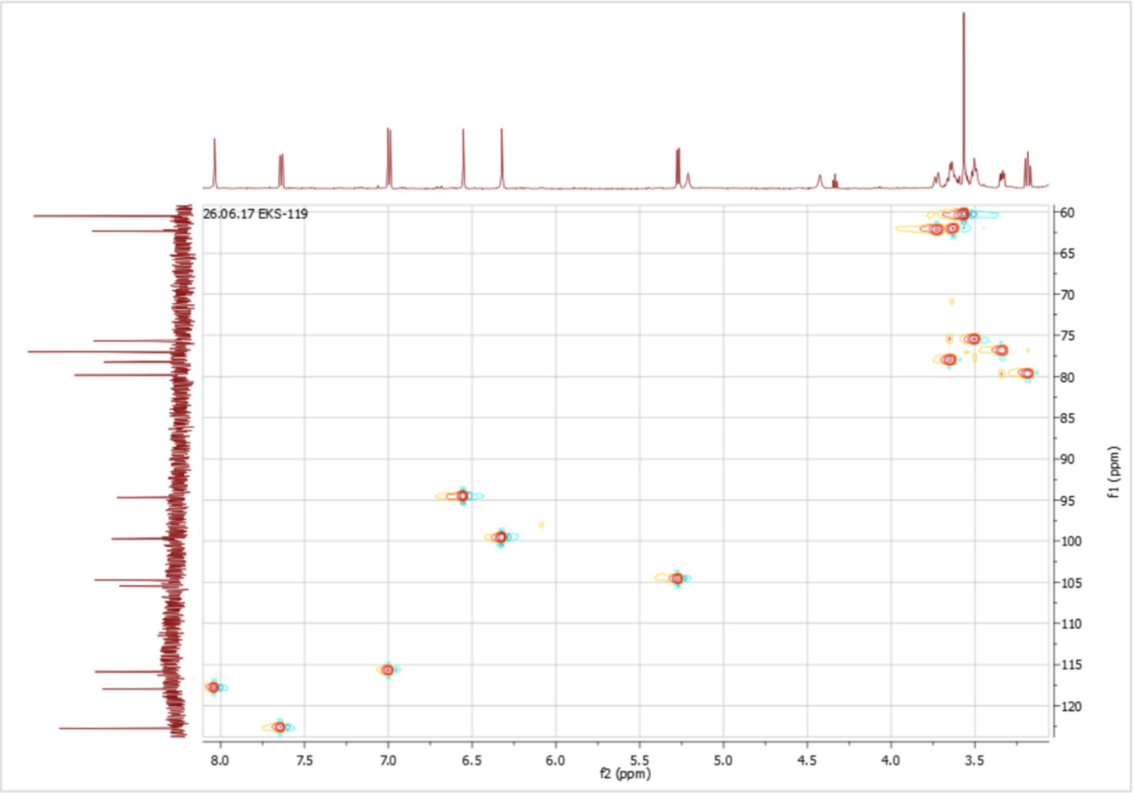

Figure S37. HSQC NMR spectrum of 3′,4′,5,7-tetrahydroxyflavone 3-*O*-*β*-D-(4″-*O*-methyl)-glucopyranoside (3a) (Acetone-d_6, 151 MHz)

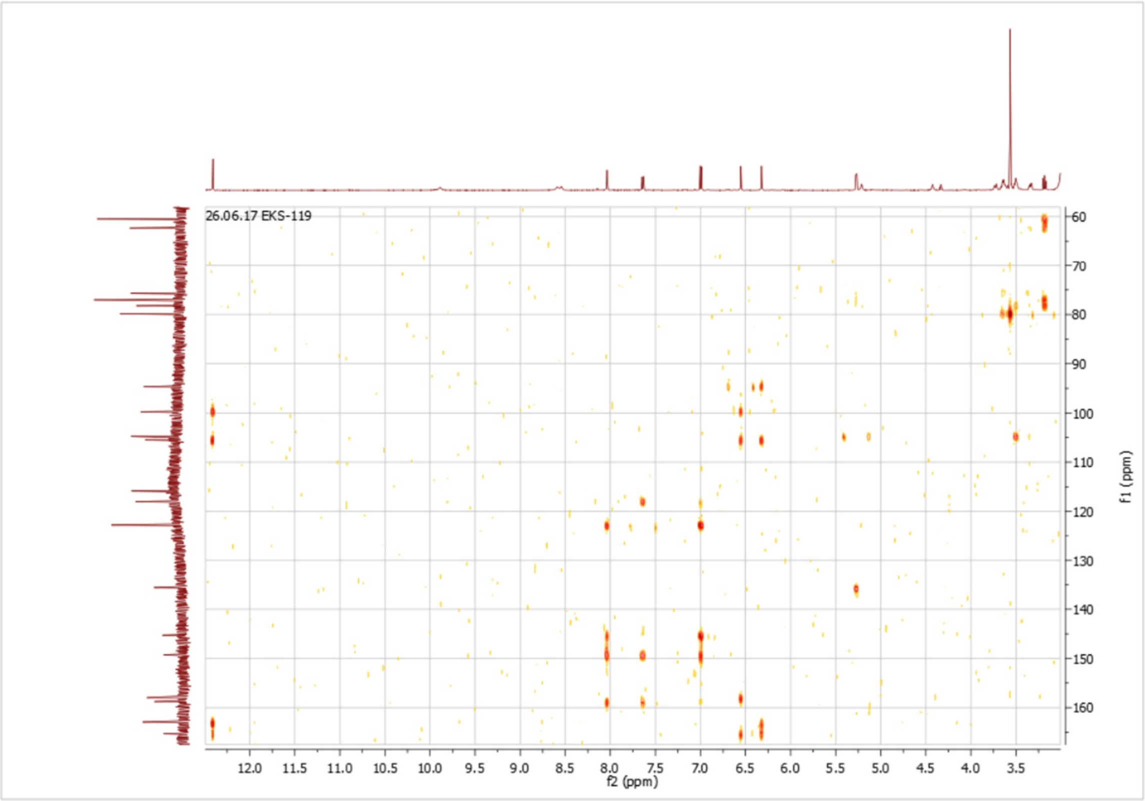

Figure S38. HMBC NMR spectrum of 3′,4′,5,7-tetrahydroxyflavone 3-*O*-*β*-D-(4″-*O*-methyl)-glucopyranoside (3a) (Acetone-d_6, 151 MHz)

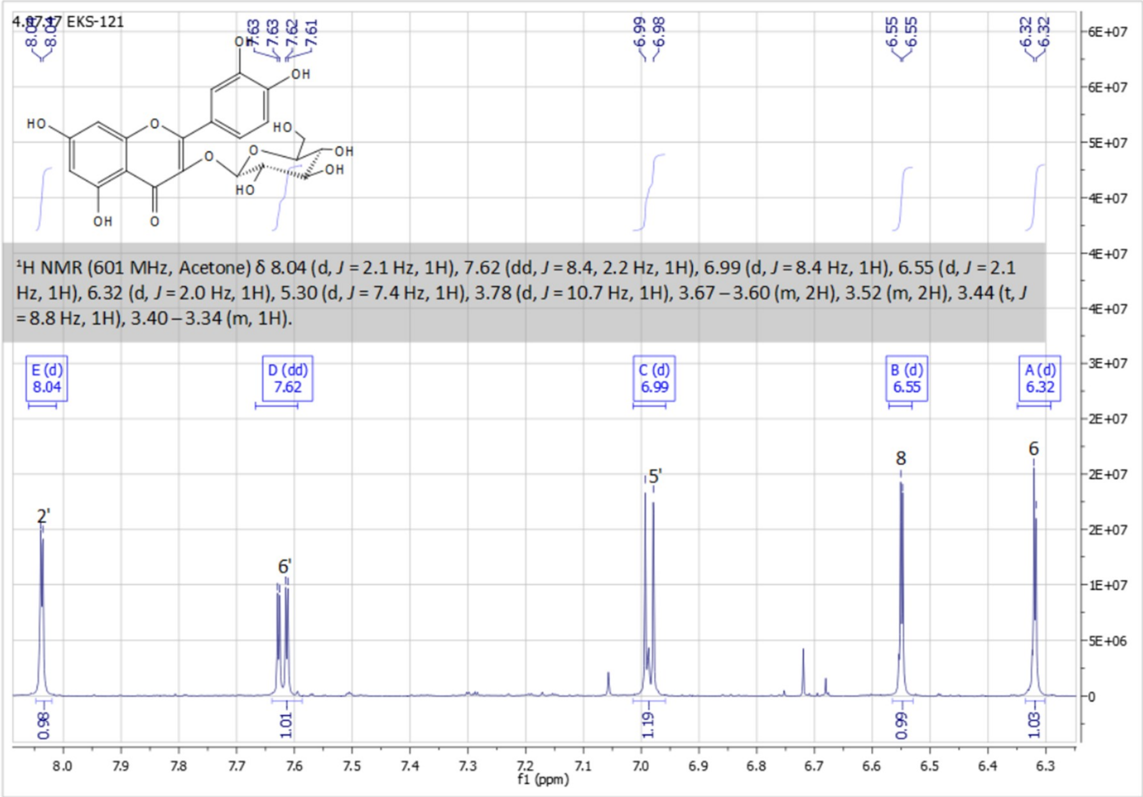

Figure S39. ¹H NMR spectrum of 3′,4′,5,7-tetrahydroxyflavone 3-*O*-*β*-D-glucopyranoside (isoquercetin) (3b) (Acetone-d6, 600 MHz)

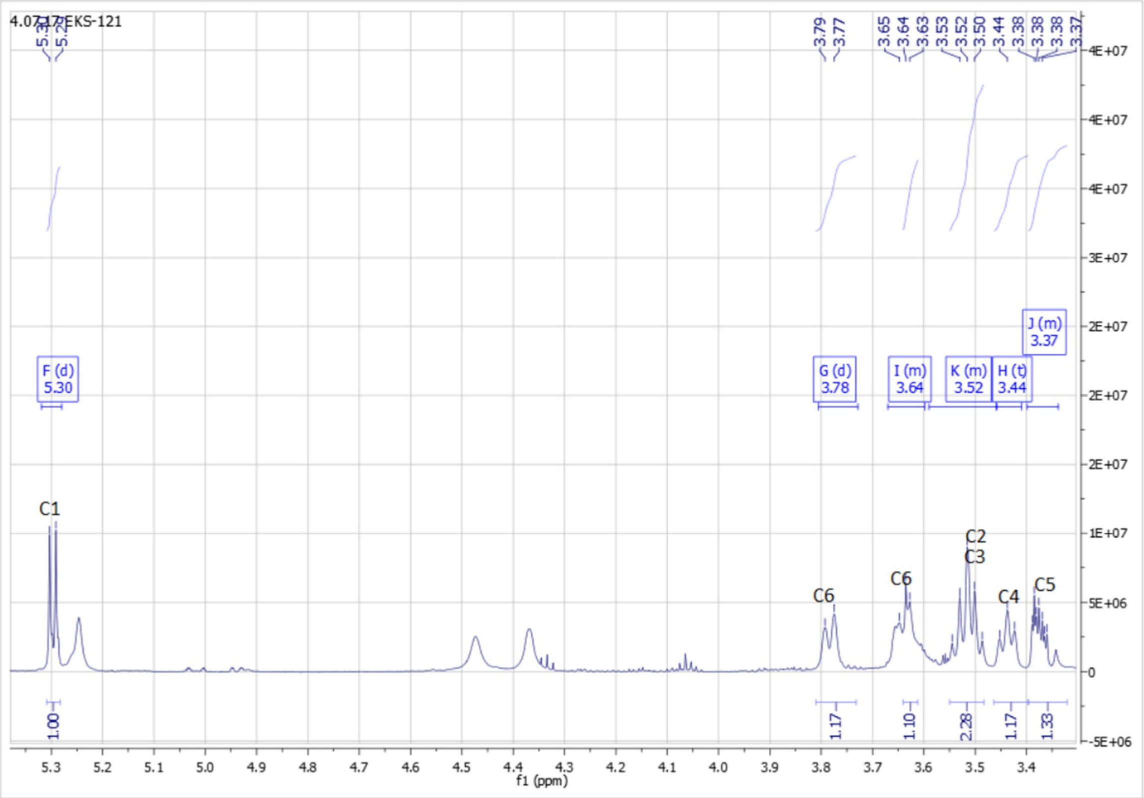

Figure S40. ¹H NMR spectrum of 3′,4′,5,7-tetrahydroxyflavone 3-*O*-*β*-D-glucopyranoside (isoquercetin) (3b) (Acetone-d6, 600 MHz)

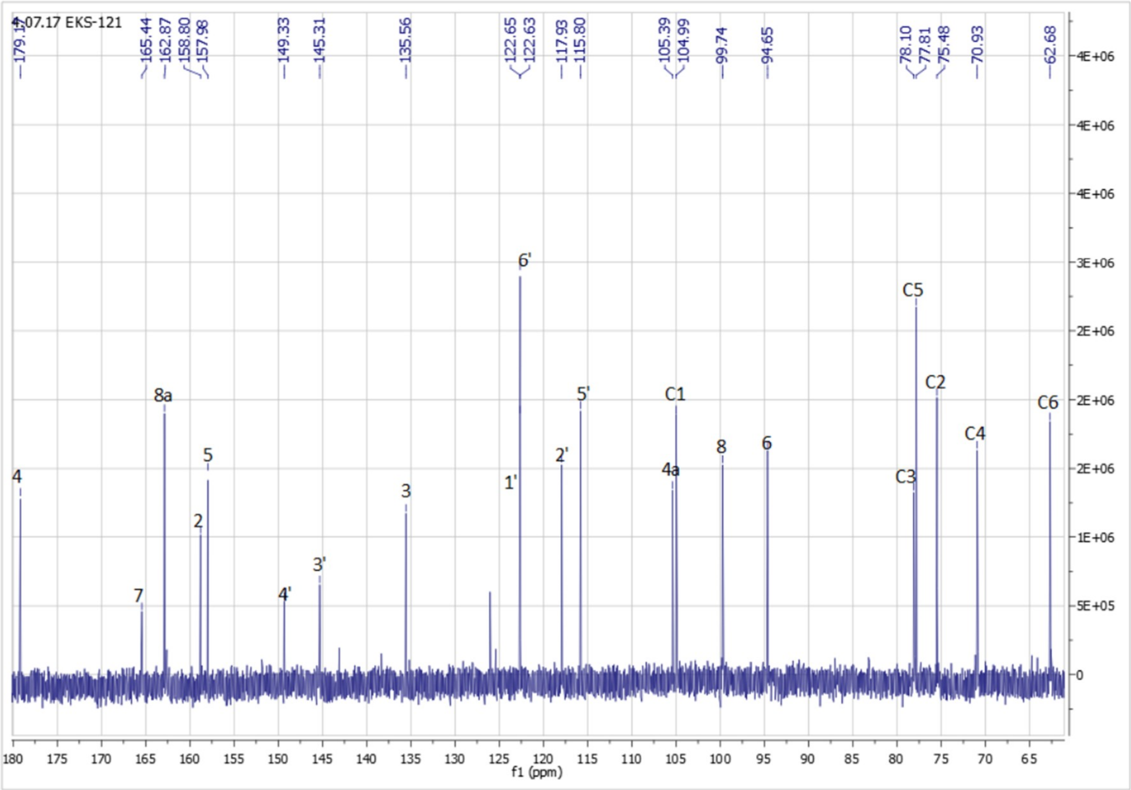

Figure S41. ^{13}C NMR spectrum of 3′,4′,5,7-tetrahydroxyflavone 3-*O*-*β*-D-glucopyranoside (isoquercetin) (3b) (Acetone-d_6, 151 MHz)

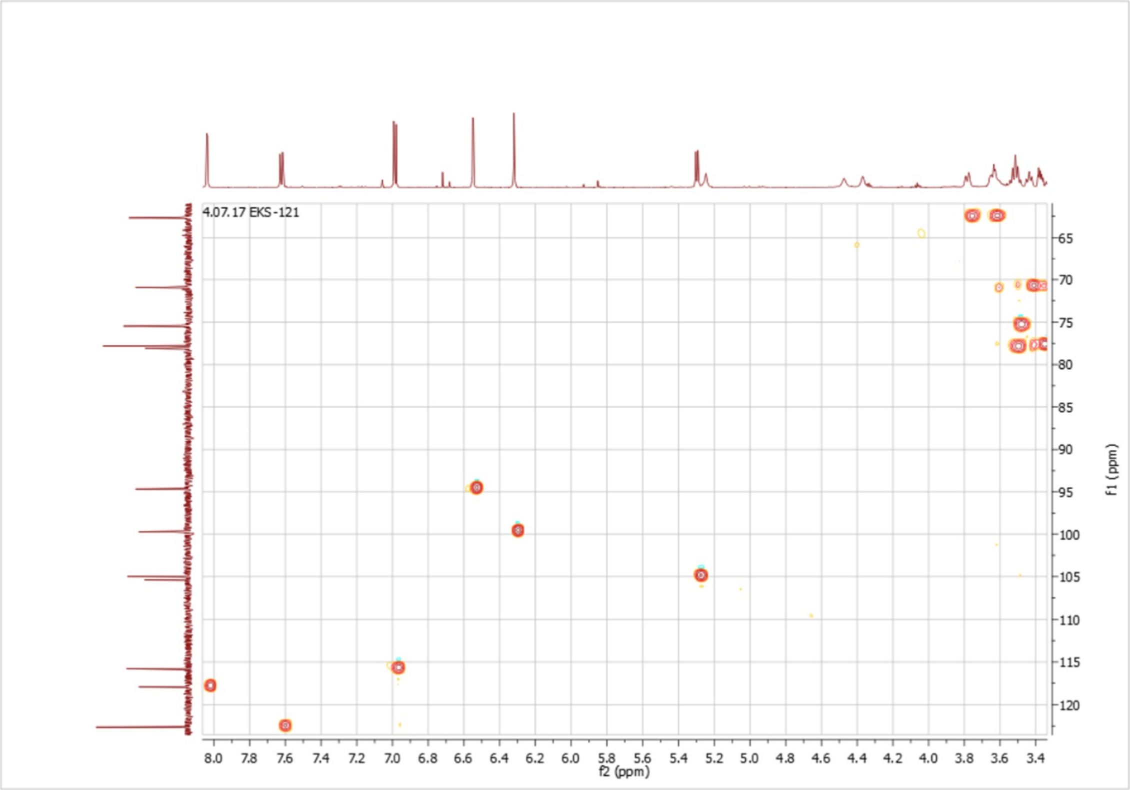

Figure S42. HSQC NMR spectrum of 3′,4′,5,7-tetrahydroxyflavone 3-*O*-*β*-D-glucopyranoside (isoquercetin) (3b) (Acetone-d_6, 151 MHz)

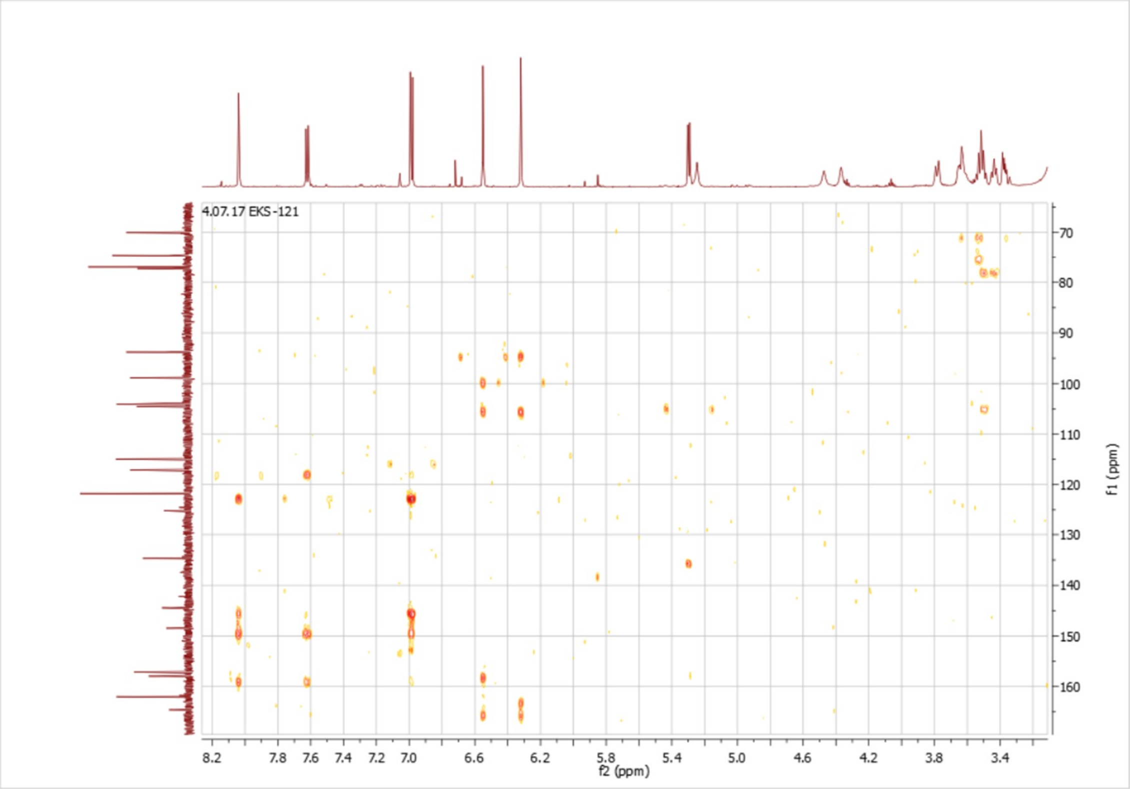

Figure S43. HMBC NMR spectrum of 3′,4′,5,7-tetrahydroxyflavone 3-*O*-*β*-D-glucopyranoside (isoquercetin) (3b) (Acetone-d6, 151 MHz)

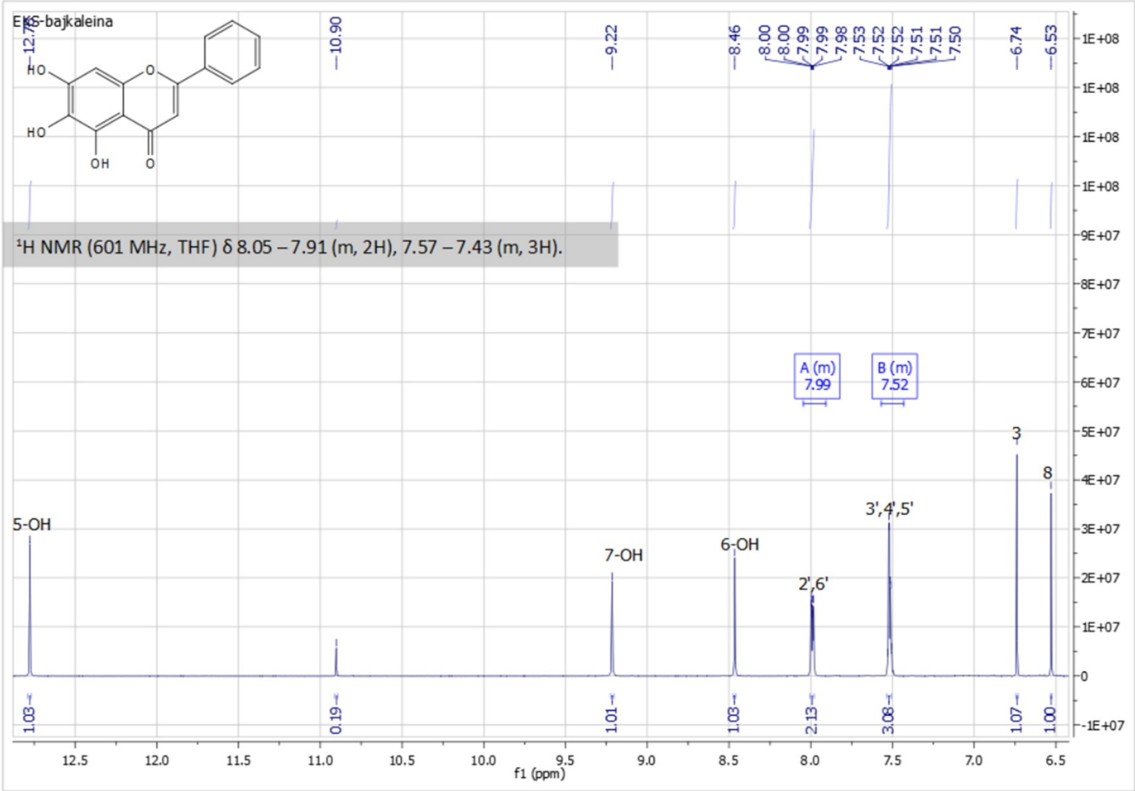

Figure S44. ¹H NMR spectrum of 5,6,7-Trihydroxyflavone (Baicalein) (4) (Tetrahydrofuran-d8, 600 MHz)

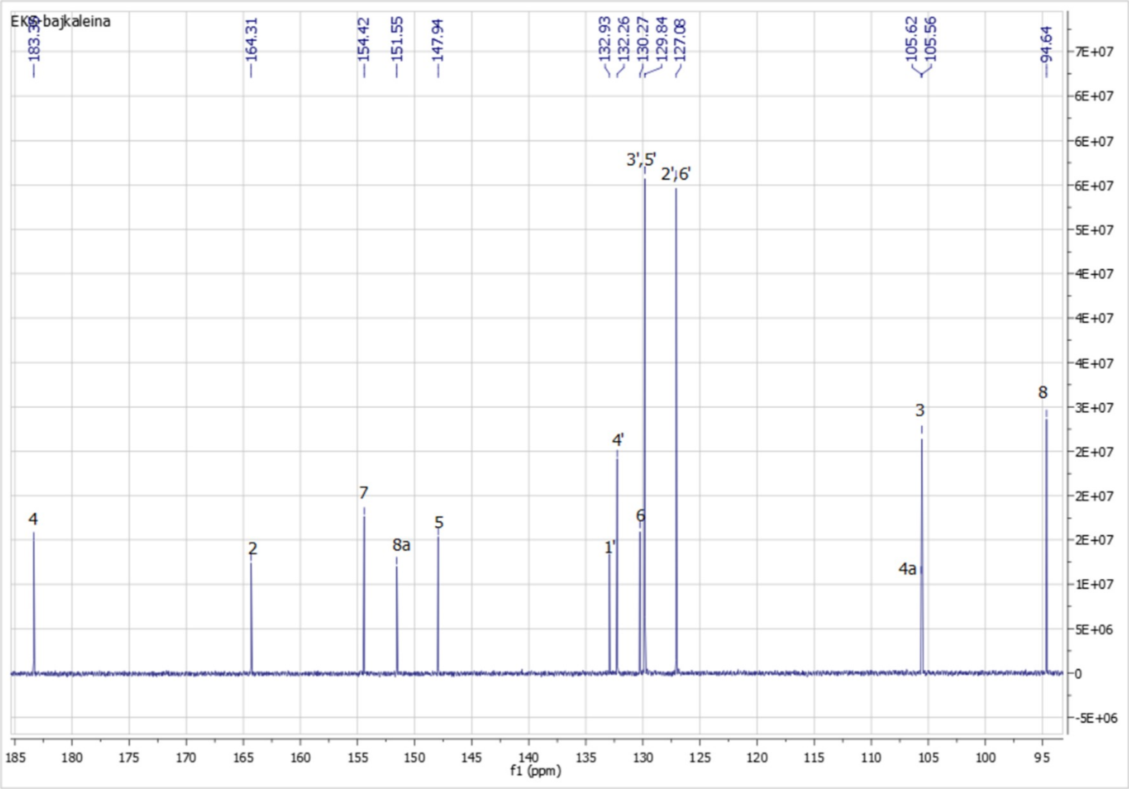

Figure S45. ^{13}C NMR spectrum of 5,6,7-Trihydroxyflavone (Baicalein) (4) (Tetrahydrofuran-d_8, 151 MHz)

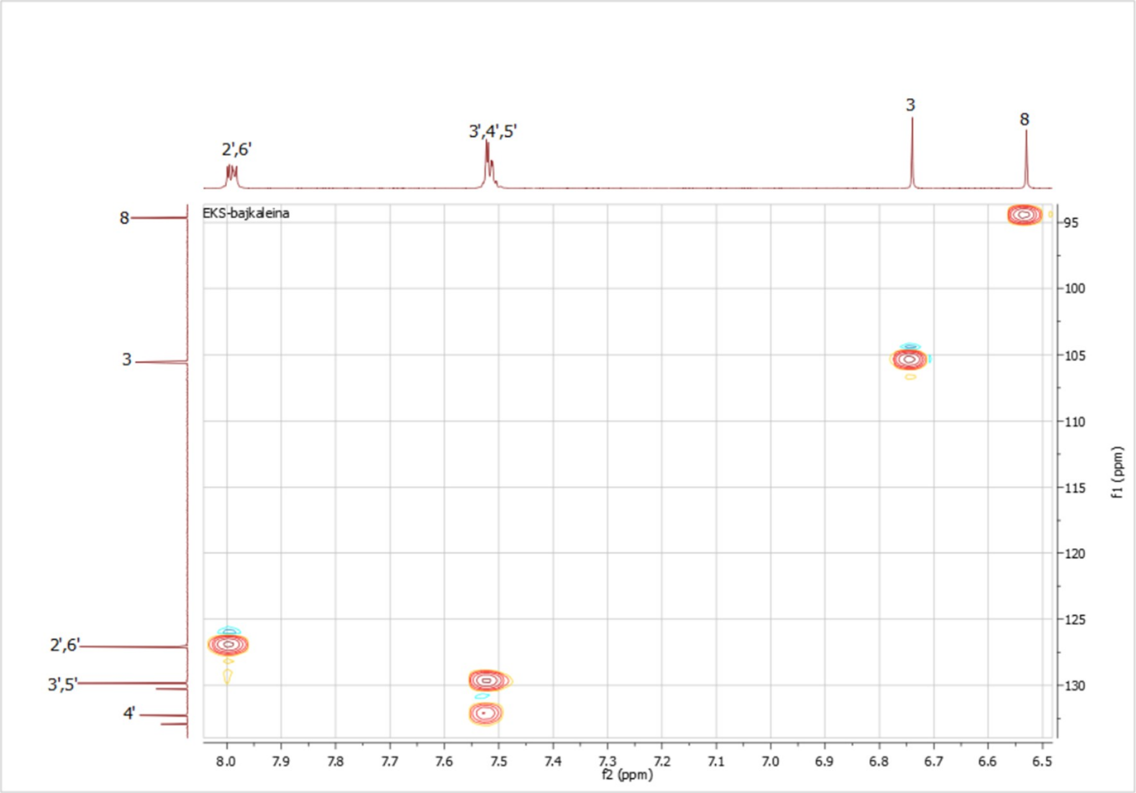

Figure S46. HSQC NMR spectrum of 5,6,7-Trihydroxyflavone (Baicalein) (4) (Tetrahydrofuran-d_8, 151 MHz)

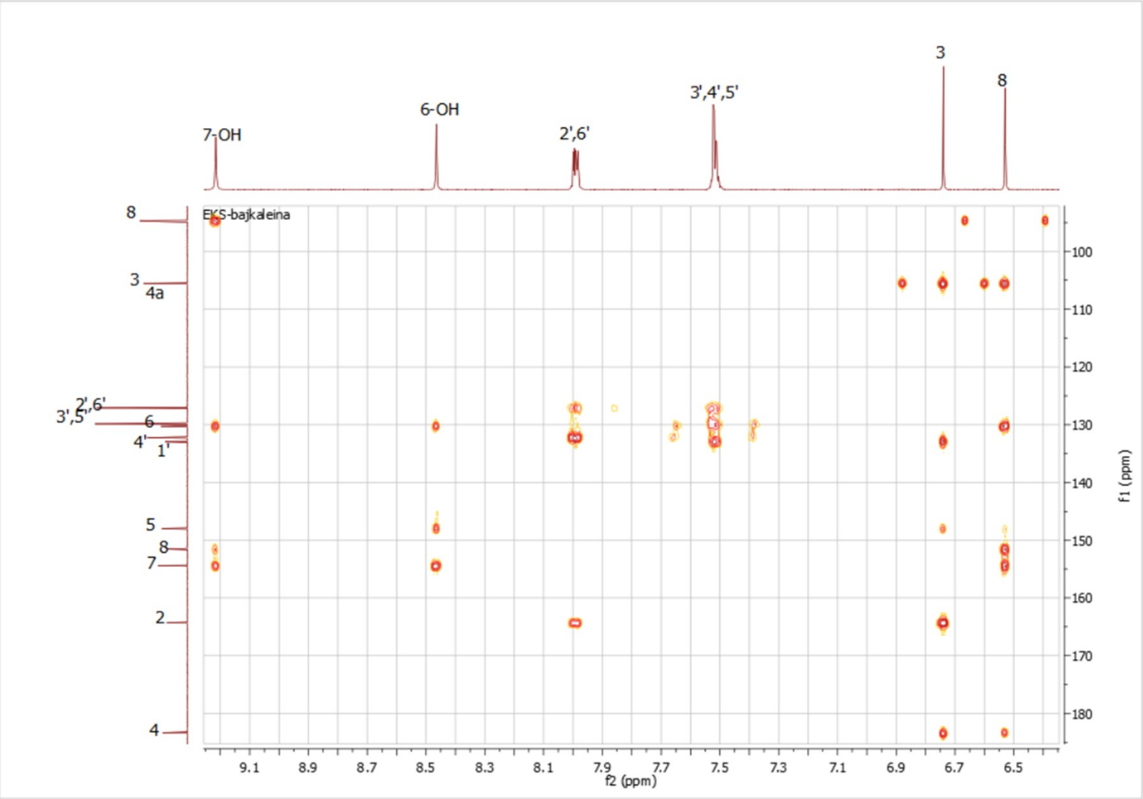

Figure S47. HMBC NMR spectrum of 5,6,7-Trihydroxyflavone (Baicalein) (4) (Tetrahydrofuran-d8, 151 MHz)

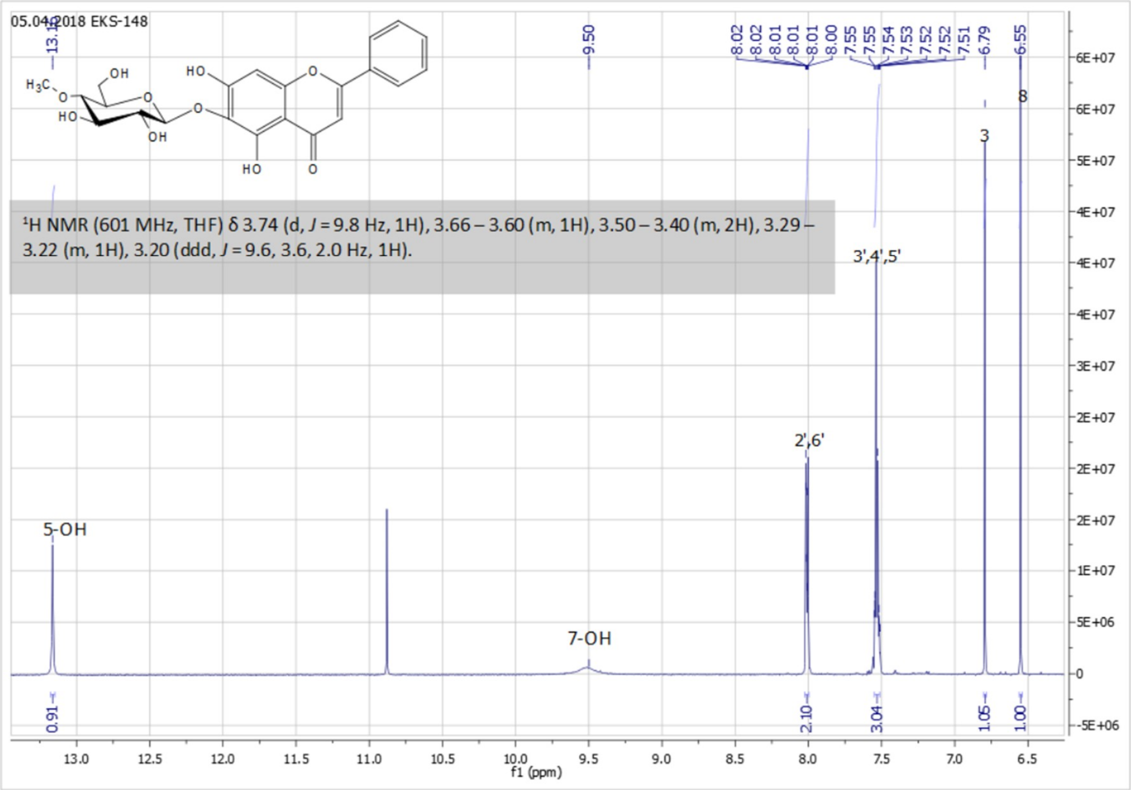

Figure S48. ^{1}H NMR spectrum of 5,7-dihydroxyflavone 6-*O*- β -D-(4''-*O*-methyl)-glucopyranoside (4a) (Tetrahydrofuran-d8, 600 MHz)

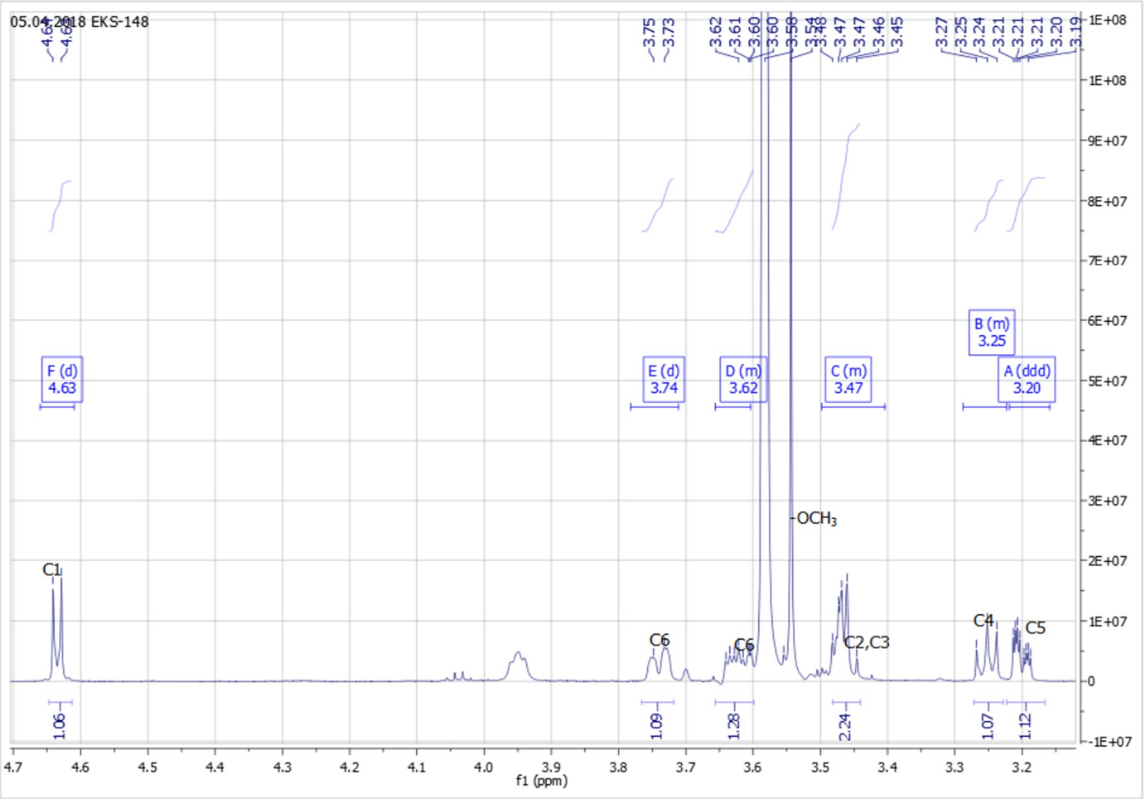

Figure S49. ^{1}H NMR spectrum of 5,7-dihydroxyflavone 6-*O*- β -D-(4″-*O*-methyl)-glucopyranoside (4a) (Tetrahydrofuran-d$_8$, 600 MHz)

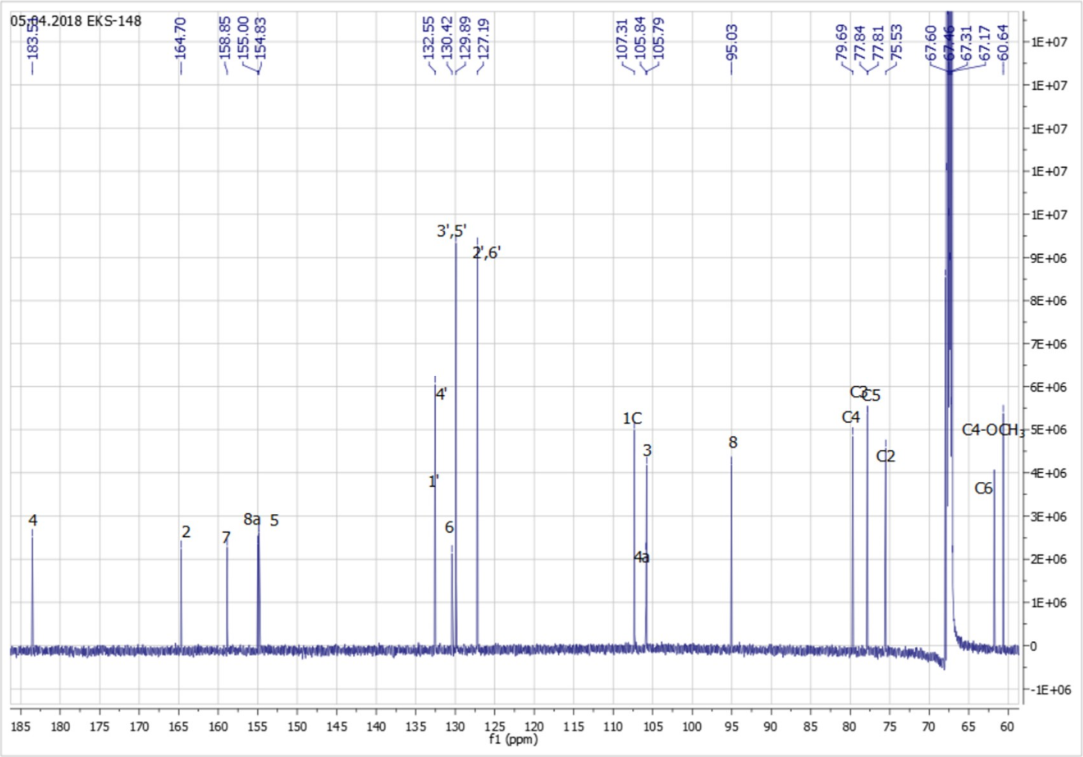

Figure S50. ^{13}C NMR spectrum of 5,7-dihydroxyflavone 6-*O*- β -D-(4″-*O*-methyl)-glucopyranoside (4a) (Tetrahydrofuran-d$_8$, 151 MHz)

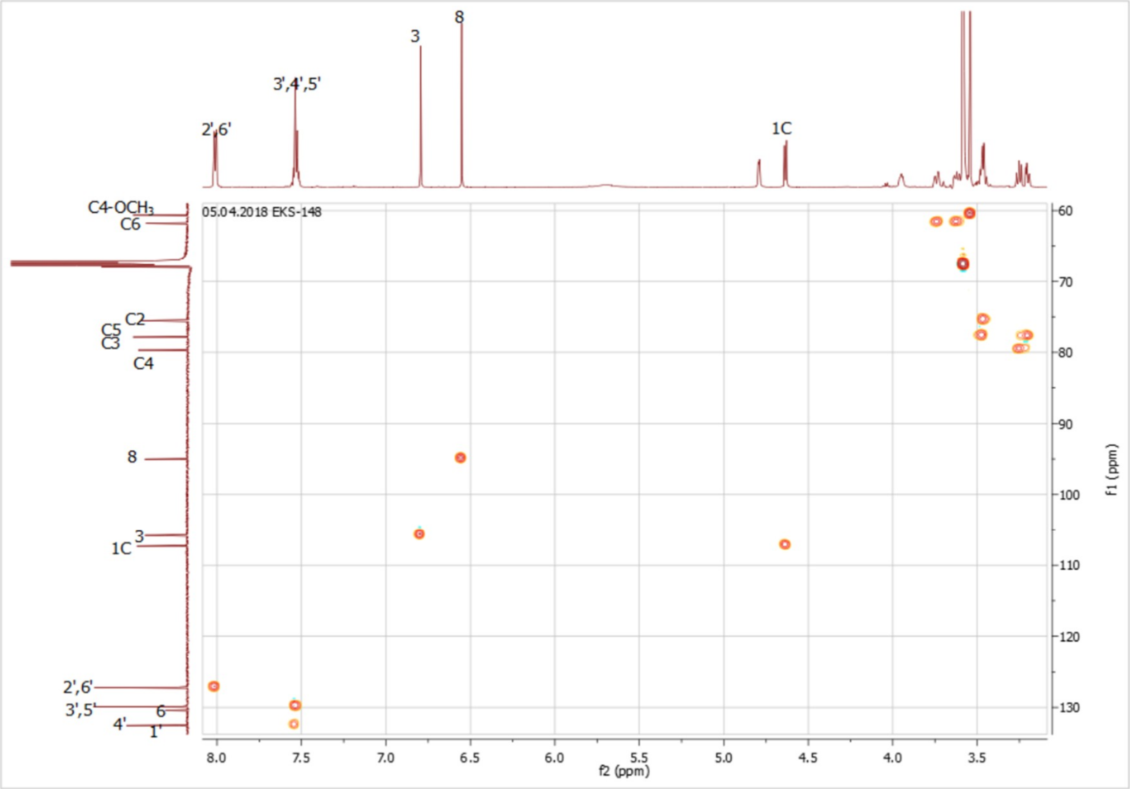

Figure S51. HSQC NMR spectrum of 5,7-dihydroxyflavone 6-*O*- β -D-(4″-*O*-methyl)-glucopyranoside (4a) (Tetrahydrofuran-d_8, 151 MHz)

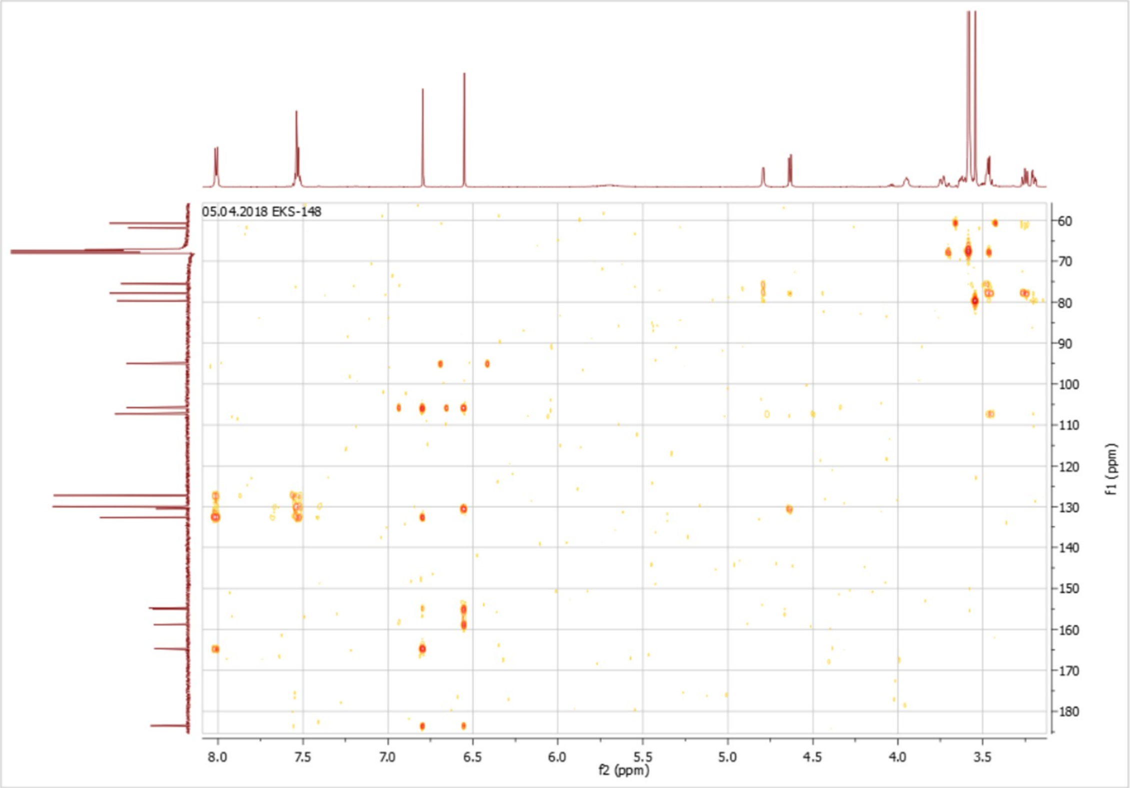

Figure S52. HMBC NMR spectrum of 5,7-dihydroxyflavone 6-*O*- β -D-(4″-*O*-methyl)-glucopyranoside (4a) (Tetrahydrofuran-d_8, 151 MHz)
